# Phenotype-driven identification of modules in a hierarchical map of multifluid metabolic correlations

**DOI:** 10.1038/s41540-017-0029-9

**Published:** 2017-09-21

**Authors:** Kieu Trinh Do, Maik Pietzner, Livia Dahlia Rasp, Nele Friedrich, Matthias Nauck, Thomas Kocher, Karsten Suhre, Dennis O. Mook-Kanamori, Gabi Kastenmüller, Jan Krumsiek

**Affiliations:** 1 Institute of Computational Biology, Helmholtz Zentrum München, Neuherberg, Germany; 2 Institute of Clinical Chemistry and Laboratory Medicine, University Medicine Greifswald, Greifswald, Germany; 3 DZHK (German Center for Cardiovascular Research), Partner Site Greifswald, Greifswald, Germany; 4 Department of Restorative Dentistry, Periodontology, Endodontology, Preventive and Pediatric Dentistry, Unit of Periodontology, University Medicine Greifswald, Greifswald, Germany; 5 Institute of Bioinformatics and Systems Biology, Helmholtz-Zentrum München, Neuherberg, Germany; 6 Department of Physiology and Biophysics, Weill Cornell Medical College in Qatar, Education City, Doha, Qatar; 7 Department of Clinical Epidemiology, Leiden University Medical Center, Leiden, The Netherlands; 8 Department of Public Health and Primary Care, Leiden University Medical Center, Leiden, The Netherlands; 9 German Center for Diabetes Research (DZD), Neuherberg, Germany

**Keywords:** Biochemical networks, Molecular biology, Complexity

## Abstract

The identification of phenotype-driven network modules in complex, multifluid metabolomics data poses a considerable challenge for statistical analysis and result interpretation. This is the case for phenotypes with only few associations ('sparse' effects), but, in particular, for phenotypes with a large number of metabolite associations ('dense' effects). Herein, we postulate that examining the data at different layers of resolution, from metabolites to pathways, will facilitate the interpretation of modules for both the sparse and the dense cases. We propose an approach for the phenotype-driven identification of modules on multifluid networks based on untargeted metabolomics data of plasma, urine, and saliva samples from the German Study of Health in Pomerania (SHIP-TREND) study. We generated a hierarchical, multifluid map of metabolism covering both metabolite and pathway associations using Gaussian graphical models. First, this map facilitates a fundamental understanding of metabolism within and across fluids for our study, and can serve as a valuable and downloadable resource. Second, based on this map, we then present an algorithm to identify regulated modules that associate with factors such as gender and insulin-like growth factor I (IGF-I) as examples of traits with dense and sparse associations, respectively. We found IGF-I to associate at the rather fine-grained metabolite level, while gender shows well-interpretable associations at pathway level. Our results confirm that a holistic and interpretable view of metabolic changes associated with a phenotype can only be obtained if different layers of metabolic resolution from multiple body fluids are considered.

## Metabolomics: phenotype-driven modules in a multifluid metabolic map

Metabolism consists of complex interactions across various organs and body fluids, which poses a substantial challenge for the analysis of metabolic data. To address this problem, Jan Krumsiek from Helmholtz Zentrum München and colleagues used metabolomics measurements of plasma, urine, and saliva from 1000 people to statistically reconstruct a map of interactions in human metabolism. Based on this map, a novel approach that identifies highly correlated biochemical modules that are associated with a given phenotype, was tested for gender and insulin-like growth factor I (IGF-I). The identified modules provided insights into the interaction between metabolome and phenotype that reach beyond what can be found by commonly used statistical approaches for metabolomics. The approach is generic and can be readily applied to new datasets by other colleagues from the field.

## Introduction

Metabolomics is the study of metabolic profiles at a global level. The metabolome is a readout of the biochemical transformations that involve small molecules in a body fluid or organ, and it reflects a snapshot of the state of a biological system.^1,2^ Therefore, metabolomics has frequently been used to identify patterns associated with various pathophysiological states in humans, such as diabetes mellitus,^3,4^ cardiovascular disease,^5,6^ and Alzheimer’s disease.^7–9^

Most published metabolomics studies focused on only one body fluid, usually blood or urine; however, phenotypes usually have links to metabolism in multiple fluids simultaneously. For example, we reported multifluid associations for type 2 diabetes in two recent studies.^10,11^ With continuous technical advancements and decreasing costs, datasets with simultaneous metabolomics measurement should become available rapidly, as can be seen by the increasing research in this field.^12–16^

**Fig. 1:**
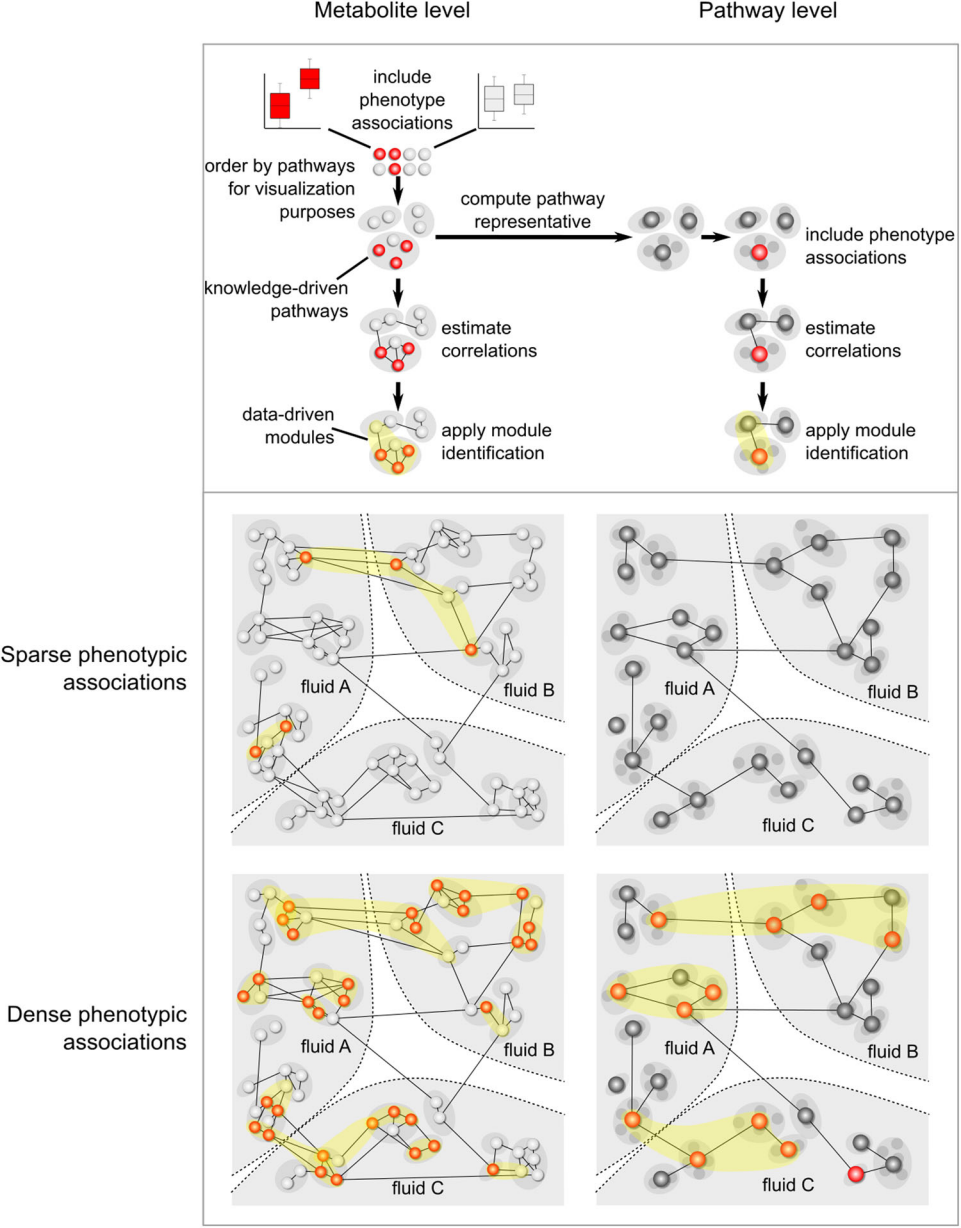
Concepts of sparse and dense phenotype associations in metabolic networks. The figure depicts the concepts of sparse (top) and dense (bottom) phenotypic associations in metabolite (left) and pathway (right) networks. Metabolites, represented as nodes, can be grouped by knowledge-driven pathway information for visualization purposes. In addition, the nodes can be colored according to their phenotype associations (e.g. determined by a *t*-test). Network inference is performed to create a network, where an edge between two nodes represents their statistical correlation. Based on this network a module identification approach is applied to search for groups of correlating entities that are related to a phenotype of interest. For the pathway analysis, metabolites of the same pathway are aggregated to generate a pathway representative, which again can be colored according to phenotype associations. A pathway network is generated by connecting two pathway representations that are statistically correlated. Finally, a module identification approach is applied on this pathway network

Phenotype associations in such large-scale, heterogeneous metabolomics datasets can be expected to be substantially complex, spanning functional modules, possibly across multiple fluids (Fig. 1). Functional modules are commonly defined as groups of correlating entities that are functionally coordinated, coregulated, or generally driven by a common biological process.^17^ Systematic module identification algorithms are well established for omics data,^17–22^ but have rarely been applied to high-throughput metabolomics data. A few metabolomics studies proceeded toward this objective by finding clusters in metabolite correlation networks, and by subsequently performing enrichment analyses with respect to a certain phenotype;^23–26^ however, none of these studies performed a systematic phenotype-driven module search. Moreover, these analyses were performed in only one single fluid.

The identification and interpretation of modules for phenotypes that show rather few ('sparse') associations with metabolomics data are usually straightforward; however, phenotypes such as gender or BMI have been described to associate with more than a third to half of the blood metabolome.^26–28^ A module search would lead to numerous results covering the majority of the metabolic network ('dense' associations), thereby impeding interpretation by their sheer quantity (Fig. 1). To solve this, we suggest performing association analysis and module identification at a coarser level, by grouping metabolites into their common pathways (defined as groups of metabolites with common biochemical and biological properties based on prior knowledge). The general idea is that while sparse phenotypic associations can only be detectable at the metabolite level, modules of dense phenotypic associations might be easier to interpret at the pathway levels.

In this study, we present a method for the systematic phenotype-driven identification of modules from multifluid metabolomics data, operating both at the single metabolite and at the pathway level. Specifically, we created a hierarchical map of multifluid metabolomics correlations as a template for the underlying metabolic network. Based on this network, we automatically extracted modules associating with two example phenotypes.

To create this hierarchical map, we generated data-driven multifluid networks from blood, urine, and saliva metabolomics data of the German Study of Health in Pomerania (SHIP-TREND) cohort.^29^ Specifically, we estimated Gaussian graphical models (GGMs) based on partial correlations at the metabolite level and at two pathway levels: 'super-pathways*'* representing metabolite classes such as 'Lipid' or general metabolic processes such as 'Energy' and *'*sub-pathways' representing biochemical subclasses or processes within a super-pathway such as 'Lysolipid' or 'TCA cycle,' respectively. The three networks (metabolite, sub-pathway, and super-pathway) together depict the hierarchical map.

Moreover, we developed a module search algorithm inspired by Chuang et al.^20^ and applied it to serum measurements of insulin-like growth factor I (IGF-I) and gender. IGF-I is a growth hormone with high sequence homology to insulin. It participates in numerous biochemical processes, in particular in the stimulation of cell growth and proliferation, and has been found to be associated with various disorders such as diabetes, cardiovascular diseases, and cancer.^30–34^ Despite its key roles in various biochemical processes, mapping IGF-I−metabolite associations onto a metabolite network in a previous study on the same dataset from the SHIP cohort resulted in only a relatively small number of blood and urine metabolites.^34^ Thus, IGF-I here serves as a trait with sparse associations. For gender associations, on the other hand, we found associations with a major part of the metabolic network,^26^ thus representing a trait with dense associations.

## Results

Our analysis was based on data from the SHIP-TREND cohort. The dataset comprised 906 individuals, 512 females and 394 males, for which fasting plasma, urine, and saliva samples were available. Untargeted metabolomics measurements were performed by ultra-high liquid-phase chromatography coupled with tandem mass spectrometry (UPLC-MS/MS). Data preprocessing included run-day normalization, dilution factor normalization (for urine and saliva), log transformation, outlier handling, and handling of missing values. After preprocessing, 610 known metabolites and 387 metabolites, whose chemical structures had not been identified yet, were available for further analysis. For each metabolite, knowledge-based pathway annotations from the metabolomics platform (Metabolon Inc.) were used. Each known metabolite was annotated with one of 73 'sub-pathways', which represent metabolic pathways or biochemical subclasses of the compounds (e.g., 'Branched-chain amino acid', 'Lysolipid', 'Glycolysis'). In addition, each sub-pathway was assigned to one of eight broad 'super-pathways' ('Amino acid', 'Lipid', 'Carbohydrate', 'Nucleotide', 'Peptide', 'Energy', 'Cofactors and vitamins', and 'Xenobiotics'). These pathway annotations have been frequently used in previous studies that investigated data from the same platform (see e.g. refs. 35–37). Metabolites, their annotations, and a comparison of the measured metabolite pools between fluids can be found in Supplementary Information S1.

### Pathway representation and generation of the hierarchical map

**Fig. 2:**
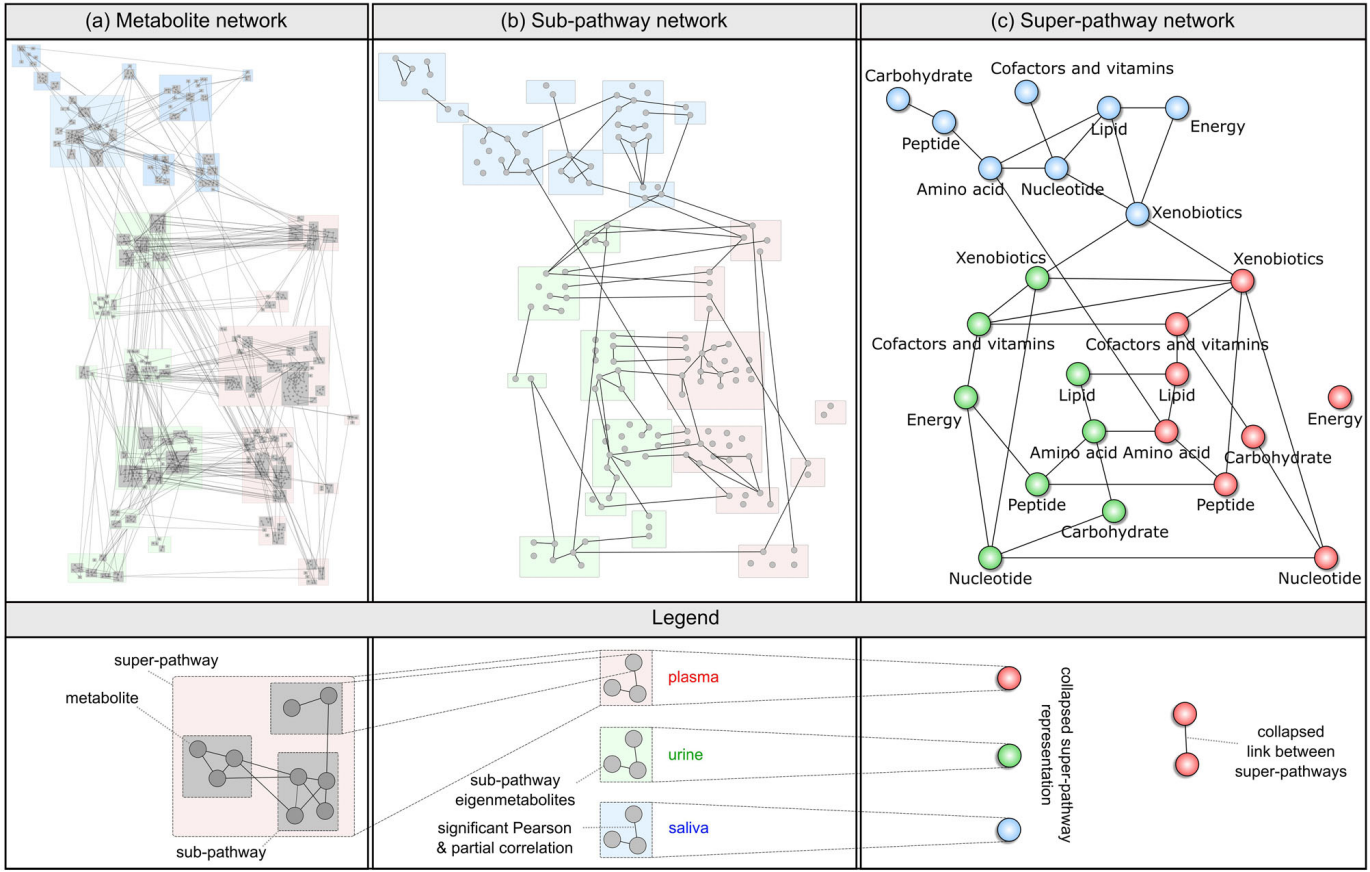
Hierarchical map of multifluid metabolic processes at **a** metabolite, **b** sub-pathway, and **c** super-pathway levels. In the metabolite and sub-pathway network, edges were drawn if both partial correlation and Pearson correlation were significant at *α* = 0.05 after Bonferroni correction for multiple testing. The super-pathway network **c** was generated by collapsing the sub-pathway GGM, i.e. drawing an edge between two super-pathways whenever at least one pair of their sub-pathways was connected. Note that all three networks share the same overall layout

We generated the hierarchical metabolic map by inferring three networks, representing the metabolic processes at three decreasing levels of granularity (Fig. 2): The first comprised multifluid correlations between single metabolites based on a GGM, a correlation-based network inference approach. Note that unknown metabolites were used to estimate the metabolite network, but were excluded from this view. To generate a sub-pathway network, a GGM was calculated based on sub-pathway *eigenmetabolites*. The majority of these *eigenmetabolites* showed a high degree of explained variance for their respective metabolites (Supplementary Information S2), and thus were reasonable statistical representatives of the pathways. To generate the super-pathway network, the sub-pathway GGM was *collapsed* by connecting any two super-pathways that showed at least one connection in the sub-pathway network. This procedure was chosen instead of calculating GGMs on the corresponding super-pathway *eigenmetabolites* due to the substantially high heterogeneity of most of the super-pathways (e.g., the very broadly defined ‘Lipid’ super-pathway). This is reflected by low-explained variances for super-pathway *eigenmetabolites* (Supplementary Information S2), which would not suffice as true pathway 'representatives'. Note that unknowns were excluded from the pathway analysis since these metabolites could not be assigned to a sub-pathway or super-pathway.

Interactive versions of the networks as yEd .*graphml* files, as well as corresponding correlation matrices, are available in Supplementary Information S3. Detailed lists of all correlation coefficients and the associated pathways can be found in Supplementary Information S4.

**Fig. 3:**
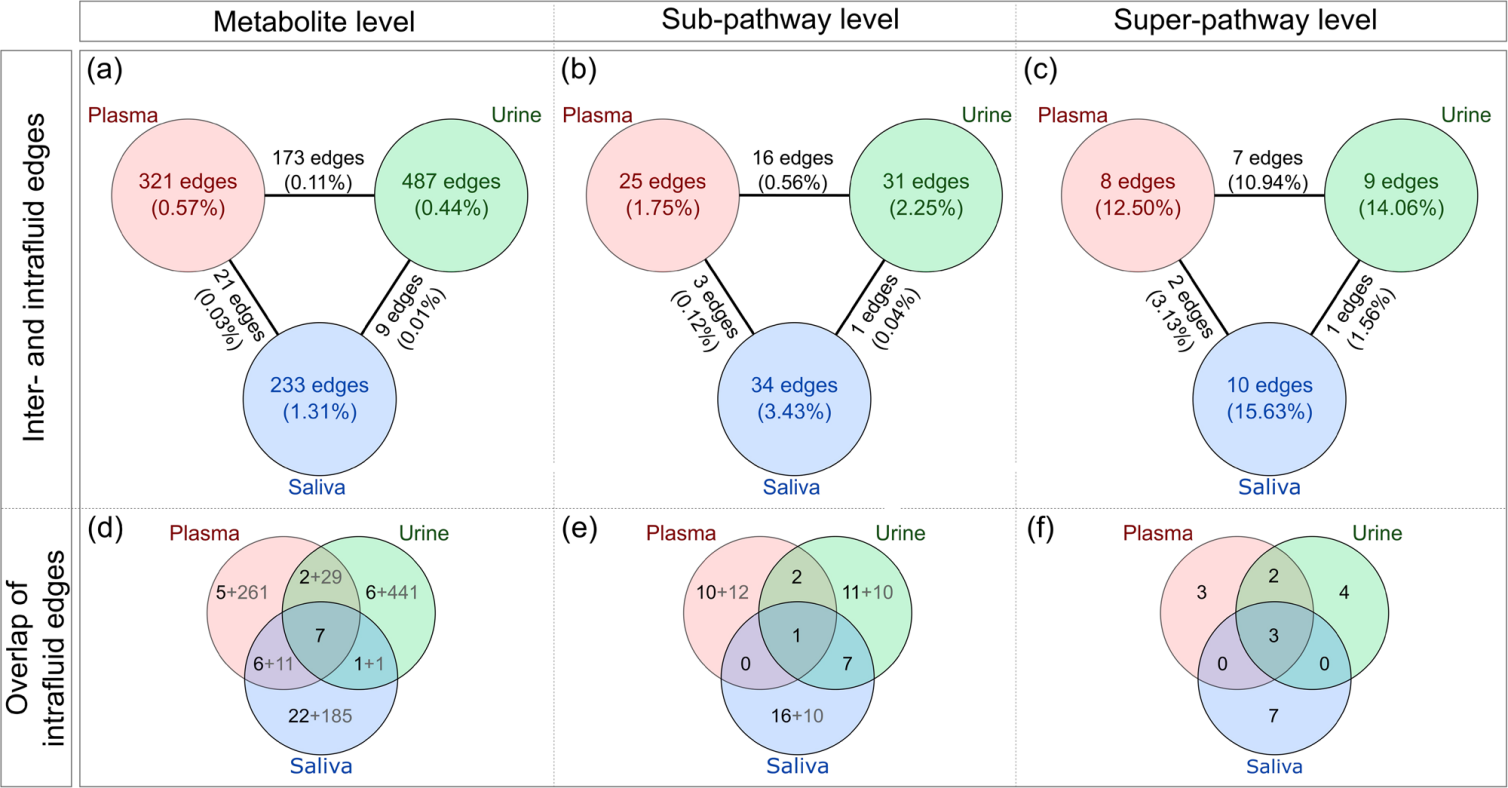
Global structure of the hierarchical map. **a**–**c** Absolute number and percentage of significant intra-fluid and interfluid edges. The percentage is calculated as the number of edges divided by all possible edges. **d**–**f** Number of intrafluid edges occurring in only one fluid or shared across two or all three fluids. Black numbers correspond to links between metabolites, sub-pathways, or super-pathways that were measured in all the three body fluids, while gray numbers represent metabolites and pathways that occur in at most two body fluids

At the most fine-grained level, the hierarchical map contained 335 plasma, 473 urine, and 189 saliva metabolites, with a total of 1244 edges between them (1041 intrafluid and 203 interfluid edges, Fig. 2a, Fig. 3a). The sub-pathway GGM comprised 54 plasma, 53 urine, and 45 saliva *eigenmetabolites*, and, in total, 110 edges out of which 90 were within fluids and 20 were across fluids (Fig. 2b, Fig. 3b). The coarsest level represented by the *collapsed* super-pathway network consisted of 24 nodes, and 27 intrafluid and 10 interfluid edges (Fig. 2c, Fig. 3c). In general, we observed most correlations to be intrafluid in all the three networks. Since, in particular, salivary metabolomics measurements can be dependent on the oral hygiene of the study participants, we investigated whether the hierarchical map was influenced by the participants’ teeth brushing behavior. Overall, we found only marginal differences in the correlation structures, which were mainly based on statistical variance rather than biologically driven by oral hygiene (Supplementary Information S5).

### Similarities and differences of correlation structures across body fluids

To obtain a general overview of the hierarchical map, we explored it at the highest level of body fluids considering two aspects: (i) How similar are the intrafluid correlation structures when comparing the three different fluids? (ii) How can the crosstalk between fluids be characterized?

#### Similarity of body fluids

For all the three fluids, we determined the fluid-specific correlations, i.e., those exclusively occurring in only one body fluid, and the correlations shared between at least two fluids (Figs. 3d–f). At all levels, the number of fluid-specific edges far exceeds the number of shared edges.

At the metabolite level, 266, 447, and 207 intrafluid edges were exclusively found in plasma, urine, and saliva, respectively. A pairwise comparison of the fluids yielded 57 edges that occurred in at least two body fluids, with the majority (31) shared between plasma and urine (Fig. 3d). Plasma and saliva shared 17 correlations, whereas urine and saliva shared only 2 correlations. Overall, 50 and 77% of the fluid-specific metabolite edges occurred within the same sub-pathway and super-pathway, respectively, while for correlations that can be found in at least two fluids 80% were observed within sub-pathways and 90% in super-pathways (Supplementary Information S4). This indicates that, if correlations are shared across fluids, the two correlating metabolites more often act in similar biochemical processes compared to exclusive correlations. Comparing all the three fluids simultaneously, an overlap can only be reasonably analyzed for metabolites that were also measured in all the three of them. Inspecting only edges between such metabolites (black numbers in Fig. 3d) left 49 intrafluid edges, of which seven occurred in all the three fluids.

At the sub-pathway level, we found 69 fluid-specific and 10 shared edges (Fig. 3e). Only one edge occurred in all fluids (‘Fatty Acid Metabolism (also BCAA Metabolism)’ with ‘Fatty Acid Metabolism (Acyl Carnitine)’). Two edges were observed in both plasma and urine, and interestingly, urine and saliva shared seven edges (Supplementary Information S4), all of which were within the same respective super-pathway. Overall, at the super-pathway level, more fluid-specific edges (14) were observed compared to the shared edges (5) (Fig. 3f).

#### Crosstalk between fluids

We investigated the crosstalk between the fluids by analyzing the interfluid correlations in the hierarchical map (Figs. 3a–c). In total, there were 203 crossfluid edges at the metabolite level (Fig. 3a). A vast majority of edges was observed between plasma and urine (173), while there were only 21 and 9 edges between plasma and saliva and urine and saliva, respectively. In total, 98 of these 203 edges (75 plasma-urine, 19 plasma-saliva, and 4 urine-saliva) were between the same metabolites measured in different fluids, for example, between plasma betaine and urine betaine (Supplementary Information S4). At the sub-pathway level, we found 20 crossfluid correlations, *collapsed* to 10 interfluid links between super-pathways (Fig. 3b). The majority of sub-pathway and super-pathway edges could again be observed between plasma and urine. Except one (‘Tocopherol Metabolism’ and ‘Food Component/Plant’), all crossfluid edges were between the same sub-pathways. Six out of eight plasma super-pathways were linked to the respective same super-pathway node in urine, reflecting the aforementioned strong connection between those two fluids (Fig. 3c). In contrast, plasma and saliva, as well as urine and saliva were connected by only a few links.

Summarizing the results from this section, we found the majority of intrafluid correlations to be fluid-specific at all levels, providing evidence for substantial discordance of correlation structures across the different fluids. All edges, in particular, the shared correlations, occurred mainly between entities of the same pathways. Our results also indicated that plasma and urine are both more similar and more strongly connected to each other than to saliva, while saliva has a higher similarity and more connections to plasma than to urine. In general, crossfluid correlations were mostly observed between the same pathways, pointing toward a substantial impact of transport and exchange processes on the metabolomes between the fluids.

### Phenotype-driven module identification procedure

We developed a procedure to identify modules associated with a phenotype at different levels of the hierarchical map. The algorithm is graphically outlined in Supplementary Information S6. Briefly, given a network, a phenotype variable, a scoring function, and a seed (=starting) node; a greedy search algorithm identifies an optimal module by score maximization. The optimal module is determined by extending candidate modules along its network edges, until no further score improvement can be achieved. Each candidate module is scored by the negative logarithmized *p*-value of a regression-based association of a representative value of all metabolites in the module with the phenotype (see Methods). Notably, a single metabolite is scored by its univariate association with the phenotype. In a final consolidation step, overlapping optimal modules, for instance, those obtained from neighboring seed nodes, are identified and combined into a maximal module.

We followed a conservative multiple testing correction approach: To be significant, the *p*-value of a module had to be lower than the significance level of 0.05 divided by the number of network nodes (Bonferroni correction at node level). In addition, we required each module’s score to be higher than the maximum score observed across all single components of the module.

The procedure was applied to two phenotypes at all the three levels (metabolite, sub-pathway, and super-pathway): IGF-I as a phenotype with sparse associations, and gender as a trait with dense associations.

### Phenotype-driven module identification for sparse associations: IGF-I

Associations of IGF-I with blood and urine metabolites in the SHIP-TREND dataset were investigated in a previous study by Knacke et al.^34^ Here, we additionally integrated metabolomics measurements from saliva. Notably, in the work by Knacke et al., IGF-I associations were analyzed for males and females separately. In our study, however, the results of a module search stratified by gender were mostly covered by modules from a joint gender analysis, which is why the latter analysis was chosen. Furthermore, Knacke et al. used a more relaxed multiple testing correction (FDR at 0.05), while in this study we applied the conservative Bonferroni correction, since we expected a substantially increased statistical power for the module-identification approach.

**Fig. 4:**
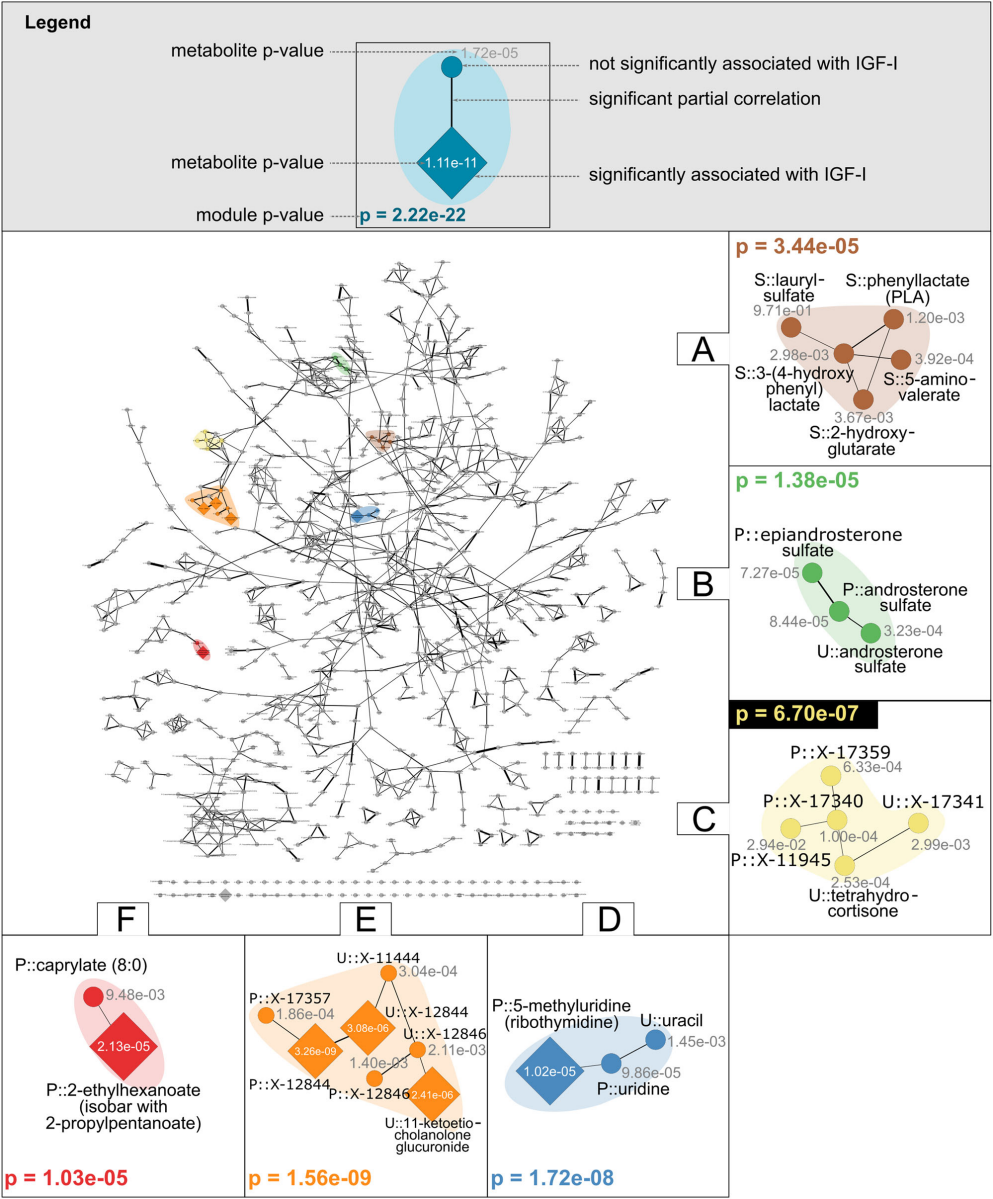
IGF-I modules. This metabolite network is a relayouted version of the metabolite GGM in Fig. 2a. Edge widths reflect absolute partial correlation values. Each colored region corresponds to an identified IGF-I module. For readability, *p*-values are given in e-notation (e.g., 1.5e−5=1.5·10^−5^). Node label prefixes P::, U::, and S:: indicate metabolites measured in plasma, urine, and saliva, respectively

At the metabolite level, our algorithm identified six modules associated with IGF-I (Fig. 4). For the sub-pathway network, we obtained only one module comprising plasma and urine ‘Steroid’ pathway metabolites (Supplementary Information S7). Furthermore, no modules were found at the coarsest level for super-pathways, confirming that IGF-I associations are rather sparse in the metabolic network. Therefore, we restricted the following analysis to the modules identified at the fine-grained metabolite level.

The six identified modules demonstrate that the module-identification algorithm enhances classical association analysis in several ways: It detected modules that (i) cover multiple pathways (see modules A and F) and (ii) span multiple body fluids (see modules B–E). (iii) Moreover, the algorithm was able to dissect apparently related but distinct processes. For example, modules C and E were in close proximity in the network and both contained a steroid amongst unidentified metabolites; however, the identification of two distinct modules suggested that they reflect two different processes that are independently associated with IGF-I levels. (iv) The algorithm increased the statistical power in several cases. For modules A–C, none of the single metabolites inside the modules was significant, whereas the entire module showed a significant *p*-value. This can be attributed to the reduction of statistical noise when aggregating concentrations of multiple metabolites.

Beyond the advantages of our approach compared to classical association analysis, we found a series of biologically interesting results. Initially, we were able to confirm previously identified IGF-I associations. For instance, it has been reported that there is a complex interplay between sex hormones and IGF-I.^38,39^ In our study, we identified a multifluid module containing a cluster of plasma and urine epiandrosterone and androsterone metabolites (module B). IGF-I has also been linked to the maintenance of physiological mitochondrial function via regulation of the expression of the mitochondrial pyrimidine nucleotide carrier PNC1.^34,40^ The association of single blood metabolites from the pyrimidine pathway with IGF-I has already been reported by Knacke et al. In the present study, we additionally observed that the aggregation of several blood and urine metabolites from this pathway (module D) yielded a considerably lower *p*-value than the single components, further supporting the link between pyrimidines and IGF-I. Both modules B and D contain metabolites from plasma and urine, indicating that not only the concentration levels of the respective metabolites in these fluids but also their crossfluid transport processes might be associated with IGF-I.

We also detected IGF-I associations, that to the best of our knowledge, have not been reported previously. We found a saliva module (A) comprising three amino acids, 2-hydroxyglutarate, a lipid, and laurylsulfate, a xenobiotic, each of which alone was not significantly associated with the phenotype. Associations of these metabolites with IGF-I have not previously been reported, in particular not in human saliva. Module F contained the xenobiotic 2-ethylhexanoate (EHA) and the fatty acid caprylate (8:0), neither of which has been reported with IGF-I to date; however, in this case, the module score seems to be mainly driven by EHA, while the fatty acid only contributes marginally to the score.

Finally, we investigated the effects of oral hygiene on the modules identified for IGF-I by correcting for the teeth brushing behavior of the study participants in the module identification process. Exactly the same modules were found, indicating that oral hygiene has no effects on metabolic changes related to IGF-I (Supplementary Information S5).

### Phenotype-driven module identification for dense associations: Gender

**Fig. 5:**
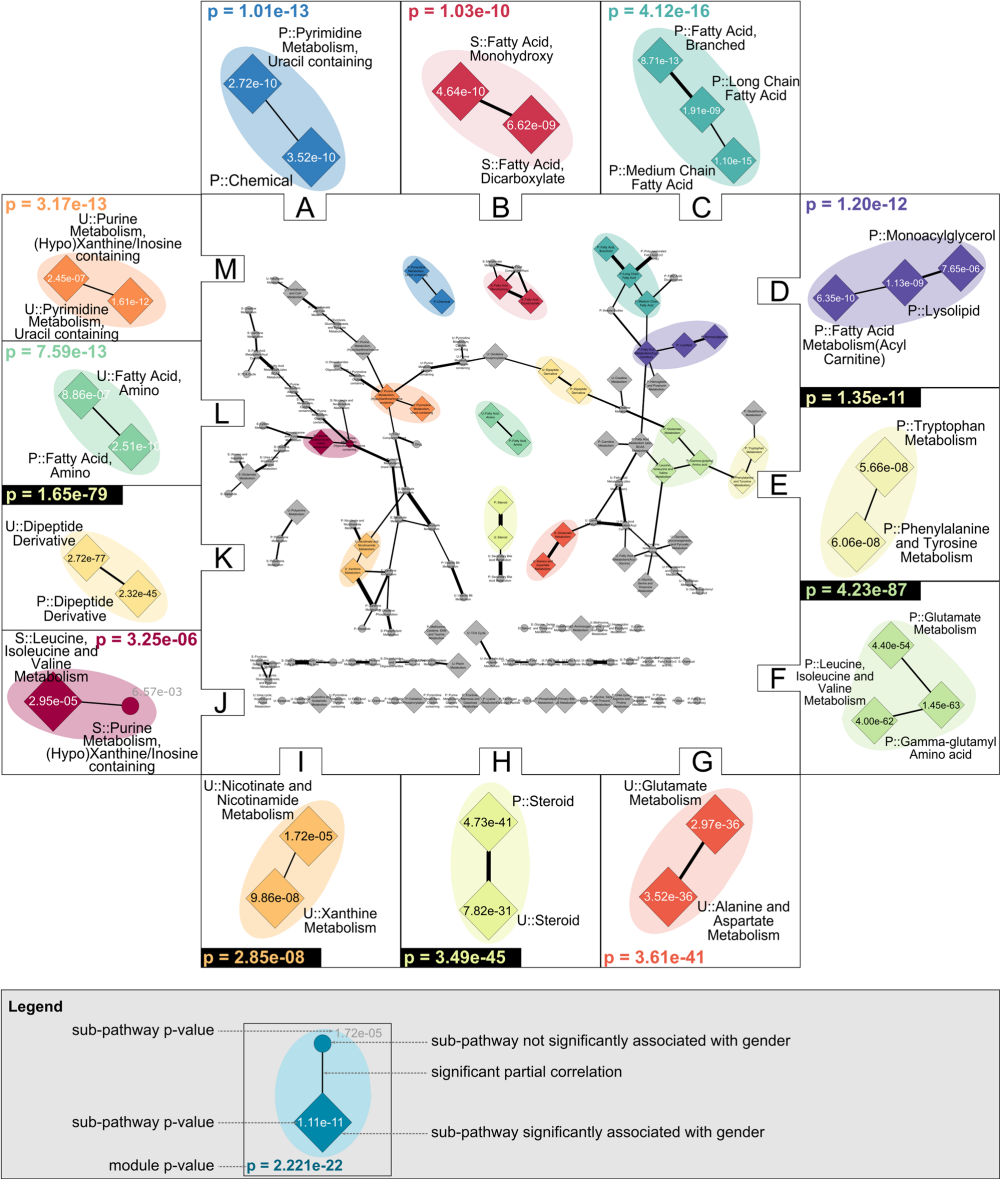
Gender modules. This sub-pathway network is a relayouted version of the sub-pathway GGM in Fig. 2b. Edge widths reflect absolute partial correlation values. Each colored region of this network corresponds to one identified module. For readability, *p*-values are in e-notation (e.g., 1.5e−5=1.5·10^−5^). Node label prefixes P::, U::, and S:: indicate metabolites measured in plasma, urine, and saliva, respectively

We next applied the module-identification algorithm with gender as phenotype, representing a trait with dense associations. As expected, for the metabolite network, we found a high number (73) of gender-associated modules (Supplementary Information S8). At the sub-pathway level, we identified 13 regulated modules (Fig. 5). Finally, at the super-pathway level, two modules indicating associations at a very global level were detected (Supplementary Information S8): the first module comprised plasma ‘Amino acid’ and ‘Peptide’, and the second module consisted of saliva ‘Carbohydrate’ and ‘Amino acid’. Herein, we focus on modules detected at the sub-pathway level, which seemed to be an appropriate compromise between the metabolite and super-pathway levels.

From the 13 sub-pathway modules, 9 were within one fluid only (Fig. 5). We observed three multifluid modules comprising both plasma and urine sub-pathways (H, K, L). These results again demonstrate the strength of our approach to find phenotype-associated processes that span multiple pathways and even multiple body fluids. We were also able to reveal more subtle, non-obvious phenotype associations as shown in module J, where the combination of a gender-associated and a non-associated pathway led to a lower *p*-value than each pathway alone. In addition, the results show that the proposed method was able to link processes that appeared to be unrelated, since they were assigned two different pathways. This is shown in module I, which consisted of the plasma sub-pathways ‘Nicotinate and Nicotinamide Metabolism’ and ‘Xanthine Metabolism’. Both pathways contain metabolites related to coffee metabolism. The former covers caffeine derivatives, while the latter consists of trigonelline, an alkaloid found in coffee. Interestingly, the module identification approach recognized the phenotype-driven relationship between these two pathways and grouped them into one gender module.

Similar to the IGF-I results, the method identified both previously reported and novel phenotype associations. A well-known metabolome–gender association, the steroid pathway, was thereby detected as a multifluid module, spanning the plasma and urine pathways (Module H). We also detected three intrafluid lipid modules (B–D), showing multiple processes within this pathway in which men and women differ. Modules C and D comprised pathways from blood, while module B consisted of salivary ‘Fatty acid, monohydroxy’ and ‘Fatty acid, Dicarboxylate’. Several metabolites of these pathways in blood and saliva were found to associate with gender in previous studies,^10,26,41,42^ but in addition, we were able to show that all pathways in saliva had a lower *p*-value when considered in combination. In module K, we found an association of histidyl peptides with gender, confirming previous findings in human muscle tissue that the female gender is associated with reduced levels of such peptides.^43,44^ Moreover, we here illustrated that this sexual dimorphism can be observed across human blood and urine. Besides confirming known gender associations, we found a module (L) comprising plasma and urinary lipids of ‘Fatty acid, Amino’, which to the best of our knowledge, have not been reported before.

We investigated whether our identified modules could be replicated in the Qatar Metabolomics Study on Diabetes (QMDiab^3^). Since the set of measured metabolites differ between SHIP-TREND and QMDiab due to different profiling platforms, we only considered metabolites measured in both cohorts for an appropriate comparison. We generated a new hierarchical map based on the reduced SHIP-TREND dataset comprising 752 metabolites in total (490 known and 262 unknown), which were grouped into 134 different sub-pathways. The module search algorithm was run at the sub-pathway level of this newly generated hierarchical map for both SHIP-TREND and QMDiab. One-third of the gender-associated modules identified in the reduced SHIP-TREND were replicated in QMDiab (Supplementary Information S9). One factor accounting for this observation was the differing number of samples, i.e., the power of the cohorts. SHIP-TREND included 906 individuals with metabolomics measurement of all three body fluids, whereas QMDiab comprised a total of 372 participants. Finally, SHIP-TREND and QMDiab also differed in the study design. In SHIP-TREND, samples of fasting individuals were collected; whereas in QMDiab, the participants were nonfasting. The SHIP-TREND was conducted in West Pomerania, Germany, whereas the individuals in the QMDiab cohort were mainly of Arab and Asian ethnicity. Moreover, in contrast to SHIP-TREND, which was designed as a healthy cohort, QMDiab was a case-control study for type 2 diabetes. Despite substantial differences in study design and metabolomics measurement, QMDiab is, to the best of our knowledge, the only available cohort comprising metabolomics measurements in plasma, urine, and saliva, and therefore the only cohort available for replication of our results. Moreover, the replication of one-third of the results despite the substantial differences between the cohorts indicated that these results are very robust and generalizable.

We investigated the effects of oral hygiene on the gender-related modules. However, this analysis might be statistically unfeasible, because teeth brushing frequency was significantly associated with gender. Nevertheless, only one module was omitted when we corrected for the effects of oral hygiene (Supplementary Information S5).

## Discussion

In this paper, we presented an approach for the phenotype-driven identification of modules associated with phenotypes at multiple scales. To this end, a hierarchical, multifluid view of metabolism at three levels of granularity was generated and analyzed for metabolomics data from plasma, urine, and saliva of 906 participants in the SHIP-TREND cohort. A hierarchical module-identification procedure was then applied to this map for IGF-I measurements and gender representing phenotypes with ‘sparse’ and ‘dense’ associations, respectively.

The hierarchical map serves as a template of human metabolism for the module identification approach. But in addition, it allows to obtain a fundamental understanding of biochemical processes captured within and across body fluids. At all levels and as expected, the majority of correlations occurred within the same fluid. Moreover, most network edges were fluid-specific, that is, solely occurred in only one fluid, suggesting diverse metabolic processes in the fluids. This can probably be attributed to the substantially different physiological roles of each fluid, capturing metabolism at various levels. Analyzing the crosstalk between fluids, correlations were mainly observed between plasma and urine, followed by plasma and saliva, while only a few edges were found between urine and saliva. The strong link between plasma and urine was expected and reflects their close relationship through the excretion and reabsorption processes in the kidneys. Blood and saliva are also physiologically connected through the salivary glands. Finally, the weak urine–saliva crossfluid correlation might reflect an indirect connection of these fluids through blood. Overall, nearly half of the crossfluid correlations were observed between the same metabolites (e.g., plasma betaine and urine betaine). In the sub-pathway network, crossfluid correlations mostly connected the same sub-pathways (e.g., plasma ‘Xanthine Metabolism’ and saliva ‘Xanthine Metabolism’). Such correlations between biochemically closely related molecules may arise due to transport and exchange processes across the fluids.

We then performed a module identification approach based on the hierarchical map. We found that IGF-I was associated with rather local parts of the metabolic network, while at the more global level (sub-pathways and super-pathways) fewer modules were detected. In contrast, for gender, we identified a large number of modules (73) in the fine-grained metabolite network. At the sub-pathway level, these numerous modules were fused into 13 sub-pathway modules, facilitating biological interpretation by providing a better overview over parts of metabolism affected by gender. Two modules were detected at the coarse super-pathway level, but did not promote biological interpretation in this case.

For both IGF-I and gender, we could confirm previously reported associations. In addition, our analyses extended these findings to multiple fluids. For example, we could extend the association between IGF-I and plasma pyrimidine metabolites to urine, which has already been reported by Knacke et al.^34^ Moreover, to the best of our knowledge, in this study, IGF-I associations were analyzed in saliva for the first time. For gender, for instance, we showed that the association with histidyl peptides appears across human blood and urine. For both the phenotypes, the identification of multifluid modules suggested that not only the concentration levels of the respective metabolites in the corresponding fluids, but also the transport and exchange processes between the fluids were associated with the phenotypes. These types of findings could only be leveraged through a module search on multifluid data.

Our results demonstrated an increase in statistical power for the phenotype-driven module identification approach compared to classical analysis. We found significant modules comprising components that were not significantly associated with the phenotype when the single components were considered alone (e.g., IGF-I modules A–C). Moreover, by combining metabolite groups from different body fluids, statistical power is also substantially increased compared to the results from just a single fluid (e.g., gender modules H, K, L). This increase is most likely due to the reduction in statistical noise while aggregating measurements of multiple metabolites. Another major advantage of the module approach lies in overcoming borders of pathway definitions, which are inherently arbitrary for any commonly available metabolite-centric or process-centric pathway annotations. Our algorithm recognizes the phenotype-driven interplay of pathways and merges them, if appropriate, as shown in gender module I. This module reflected the well-known gender association with metabolites from caffeine metabolism,^45^ which were originally assigned to two different pathways according to sub-pathway definitions in this study.

The present study could be extended in several directions. (i) We used pathway annotations provided by the metabolomics platform, which are analogous to KEGG pathways.^46^ For future studies, other pathway definitions, such as in the HMDB^47^ or MetaCyc^48^ could be used; however, since a large number of measured metabolites will not be covered in those databases,^49^ this approach would currently result in a substantial loss of information. (ii) Following the *eigengene* approach,^50^ we defined a pathway representative as the first principal component of its metabolites. To capture a higher degree of variance explained, multivariate association methods, such as canonical correlation analysis^51^ and O2-PLS^52^ could be adapted to model the relationships between the two pathways. (iii) Our module identification approach is only suitable for finding modules where all components show the same direction of association with the phenotype (all positively or all negatively associated), while opposing effects will cancel out. A possible solution to this restriction would be the use of the multivariate modeling approaches mentioned in the previous point. (iv) We used data from the Qatar Metabolomics Study on Diabetes (QMDiab, Supplementary Information S9) to replicate the gender results. It would be interesting to also replicate our IGF-I results in a suitable cohort. To the best of our knowledge, no dataset comprising human plasma, urine, and saliva metabolomics data, as well as measurements of IGF-I are currently available besides the SHIP study. Moreover, to the best of our knowledge, QMDiab is the only available cohort comprising metabolomics measurements of plasma, urine, and saliva from the same individuals. (v) We applied our module identification approach to a multifluid metabolomics dataset. Owing to the rapid progress in high-throughput technologies, other omics data types have become readily available. It would be particularly interesting to include SNPs or transcripts, for instance, into the network.

In conclusion, we introduced a hierarchical map, that besides serving as a template of human metabolism for the module identification algorithm, can also be a valuable, downloadable resource for future studies, since it allows for a fundamental understanding of the complex correlation structure within and across multiple body fluids. Based on this map, we proposed an approach for the phenotype-driven identification of modules spanning multiple pathways and multiple body fluids. These modules provide deeper insights into mechanistic aspects of phenotype associations. Importantly, our module approach is generic, and therefore widely applicable. An R implementation of the algorithm is freely available as supplementary material for this paper. It can be used directly for any other dataset, given the presence of a data matrix, annotations of the respective variables, and a phenotype.

## Materials and methods

### Study cohort, metabolomics, and IGF-I measurement

Metabolomics data were obtained from the Study of Health in Pomerania (SHIP-TREND), conducted between 2008 and 2011 in West Pomerania, Germany, with 4420 participants. The study was approved by the local ethics committee and conformed to the principles of the Declaration of Helsinki. Written informed consent was obtained from all participants. Details about sample acquisition and experimental procedures can be found elsewhere.^29,34^ Briefly, metabolomics measurements were performed for a subset of 1000 participants without self-reported diabetes. The dataset included 561 females and 439 males with an age distribution of 50.14 ± 13.17 (mean ± SD) and 50.08 ± 14.24, and a BMI distribution of 26.99 ± 5.12 and 27.85 ± 3.7, respectively. Fasting (≥8 h) plasma and urine samples were collected between 07:00 and 12:00 am. Blood was sampled from the cubital vein of subjects in a supine position. Samples were stored at −80 °C. Stimulated saliva was collected with a commercially available collection system (Salivette®). The subjects chewed a plain cotton roll for exactly 1 min to stimulate salivation. The rolls with the absorbed saliva were placed into the Salivette® and immediately centrifuged at 1000×*g* for 20 min at 4 °C to remove food remnants, insoluble material, and cell debris. The resulting supernatant was stored at −80 °C. Samples were analyzed on an untargeted metabolomics platform established by Metabolon Inc. (Durham, USA) with ultra-high liquid-phase chromatography coupled with tandem mass spectrometry (UPLC-MS/MS) in both positive and negative modes. The measurements were performed at the Genome Analysis Center, Helmholtz Zentrum, Munich, yielding a total of 1665 metabolites across all fluids, of which 1190 represented unique metabolites. Blood IGF-I concentrations were determined by automated two-site chemiluminescent immunoassays on the IDS-iSYS kit (Immunodiagnostic Systems, Boldon, UK).

### Preprocessing and quality control

To correct for daily variations of the metabolomics platform, raw ion counts of each metabolite were rescaled by their respective median value on the run day. To ensure valid medians, metabolites with fewer than three measured values for more than the half of the run days were filtered out. This procedure resulted in 1317 total (475, 558, and 284 metabolites for plasma, urine, and saliva, respectively) and 991 unique metabolites from all three body fluids. Probabilistic quotient normalization (PQN) was then applied to urine samples to account for diurnal variation. PQN has previously been shown to be superior to common creatinine scaling.^53^ PQN was, moreover, used to normalize saliva measurements for dilution variations. For the PQN procedure, first a ‘pseudo-sample’ (reference) was calculated as the mean of all metabolites with no missing entries for all participants (131 urine and 37 saliva metabolites). Subsequently, a dilution factor was estimated as the median quotient between the reference and each sample. Finally, all measurements were divided by the respective dilution value. Of note, urine creatinine and the estimated urinary dilution factor were substantially correlated (*r* = 0.91, *p* < 0.001) within the SHIP-TREND data (Supplementary Information S1).

All metabolite levels and serum IGF-I measurements were log_2_-transformed. Multivariate outlier detection (using only metabolites with no missing values across all samples) was performed separately for all fluids using an algorithm proposed by Filzmoser et al. (2008),^54^ implemented in the *pcout* function within the R package *mvoutlier*. Briefly, this algorithm calculates an outlier score for each sample using principal component analysis and the Mahalanobis distance on a robustly scaled data matrix. Default parameters were used for the identification process, and the exclusion criterion was set to 4 SD. As a result, 13, 8, and 16 samples from plasma, urine, and saliva, respectively, were excluded from further analyses. After these preprocessing steps, the dataset comprised 906 individuals for which fasting plasma, urine, and saliva samples, as well as IGF-I measurements were available.

Since for the network inference procedure a fully observed data matrix is required, missing values were imputed by the following procedure: All metabolites with more than 20% missing values (320) were excluded from the dataset to avoid false positive results and to preserve statistical power. The first step of imputation was performed per run day on the log-transformed raw data (before normalization). Following the assumptions that missing values occur due to a detection threshold and that metabolites are log-normally distributed, each missing value was replaced by a random value drawn from the censored part of a normal distribution reconstructed by maximum-likelihood estimation.^55,56^ To ensure robust parameter estimation for the truncated normal distributions, this procedure was only applied to metabolites for which the respective run day contained more than 10 nonmissing concentration values. Remaining missing values were imputed with the *mice* R package (version 2.22) with predictive mean matching as an elementary imputation model. Note that we also used the stringent threshold of 20% to exclude variables with missing values, because estimating (partial) correlations based on too many imputed values that in turn were generated by mice using the covariance structure of the data might introduce unwanted bias. The final imputed dataset consisted of 906 samples and 997 metabolites.

### Metabolite pathway representation

Each metabolite with known chemical structure (610 metabolites) was annotated with one of the 73 sub-pathways (such as ‘Lysolipid‘, ‘TCA Cycle‘, ‘Glycolysis‘, ‘Branched-chain amino acid‘), and one of eight more general super-pathways (‘Amino acid’, ‘Lipid’, ‘Carbohydrate’, ‘Nucleotide’, ‘Peptide’, ‘Energy’, ‘Cofactors and vitamins’, and ‘Xenobiotics’). The remaining 387 metabolites have unknown chemical structure (*unknowns*), and thus, cannot be assigned to any pathway for which reason they were excluded from the pathway analyses. A detailed list of metabolites and their annotated pathways is provided in Supplementary Information S1.

For each sub-pathway, a principal component analysis was performed after scaling all variables to a mean of 0 and a variance of 1. The first principal component was used as a representative value for the entire set of metabolites in the pathway. These *eigenmetabolites*^23,50,57^ were then subjected to the network inference procedure below.

### Network inference

Two networks were inferred using GGMs, one for metabolite concentrations (all metabolites) and one for the sub-pathway *eigenmetabolites* (unknowns excluded) using the *GeneNet* R package, version 1.2.12. GGMs are based on partial correlations, which represent the linear associations between two variables corrected for all remaining variables in multivariate Gaussian distributions. We included age, gender, and BMI as standard covariates into the model. Edges between metabolites or sub-pathways were assigned if both their Pearson correlations and their partial correlations were statistically significant with *α* = 0.05 after the Bonferroni correction for $\left( {{p}\atop{2}} \right)$ tests, where *p* is the number of metabolites or sub-pathways, respectively.

To obtain a global view of connections between the super-pathways, the sub-pathway GGM was *collapsed* into a super-pathway network. To this end, a link between two nodes was drawn if there was at least one connection between any two sub-pathways assigned to the two respective super-pathways in the underlying sub-pathway GGM.

### Module identification algorithm

#### Module representatives

For a *candidate module M*, a representative value *R*_*M*_ is defined as the average of scaled intensities (average *z*-score) of all metabolites in *M*. If *M* consists of sub-pathways, then the representative is calculated as the mean *z*-score of all metabolites in the set union of all sub-pathways. Notably, for pathway network estimation, a pathway representative was defined as the sub-pathway *eigenmetabolite* based on the assumption that pathway components share common chemical and biological properties. In contrast, we chose to use mean *z*-scores as module representatives, since modules are considerably more heterogeneous.

#### Scoring function

The score of each candidate module *M* is obtained from the multivariable linear regression model

1 $${R_M} \sim{\beta _{M,0}} + {\beta _{M,1}} \cdot P + {\beta _{M,2}} \cdot {\rm{gender}} + {\beta _{M,3}} \cdot {\rm{age}} + {\beta _{M,4}} \cdot {\rm{BMI}} + {{\it{ \in }}_M}$$

where *R*_*M*_ is the aforementioned representative value, *β*_*M*,__0_ is the intercept, *β*_*M*,__1,_…,*β*_*M*,__4_ are the regression coefficients for each independent variable, *P* is the phenotype of interest, and *ϵ*_*M*_ is a normally distributed error term. The module score is then defined as the negative logarithmized *p*-value of the coefficient *β*_*M*,__1_, which represents the magnitude of phenotype association. Notably, the score of a single component equals its negative logarithmized *p*-value from a univariate analysis. Furthermore, the scoring function for gender does not contain gender as a covariate.

#### Module identification

Given the scoring function and an initial node (seed node), a greedy search procedure is performed to identify an *optimal module*. In every iteration, each neighboring node of the *candidate module* is added and the score of the extended module is calculated. The neighbor leading to the highest score improvement is then added to the module. Furthermore, a neighbor is only added if the score of the new module is higher than the scores of all single components. The algorithm terminates if no further improvements can be made. In a final step, overlapping *optimal modules* from different seed nodes are combined into a single module (*maximal module*), which is rescored by the scoring function.

For the identification of IGF-I-associated and gender-associated modules, the procedure was applied to all three networks, namely, the metabolite, the sub-pathway, and the super-pathway networks. To assess the significance of the modules, a conservative multiple testing correction procedure was used with a significance level of *α* = 0.05 after the Bonferroni correction for the total number of nodes in the underlying network. The proposed algorithm is visually described in Supplementary Information S6 and available as R code in Supplementary Information S10. An example of how to execute the R scripts in S10 is explained in S11.

## Data availability

The data that support findings of this study are available from the Ernst-Moritz-Arndt-Universität Greifswald but restrictions apply to the availability of these data (the informed consent given by the study participants does not cover data posting in public databases), which were used under license for the current study, and so are not publicly available. Data are however available from the authors upon reasonable request and with permission of the Ernst-Moritz-Arndt-Universität Greifswald or can be directly applied for via www.fvcm.med.uni-greifswald.de/dd_service/data_use_intro.php?lang=ger.

### Code availability

An implementation of the generic approach and an example script is freely available as supplementary material (Supplementary Information S10 and S11).

## Electronic supplementary material

Supplementary Material

## Electronic supplementary material

**Supplementary information** accompanies the paper on the *npj Systems Biology and Applications* website (10.1038/s41540-017-0029-9).
