# Supplementary material for: Phenotype-driven identification of modules in a hierarchical map of multifluid metabolic correlations
Source: NPJ Syst Biol Appl. 2017 Sep 21;3:28. doi: 10.1038/s41540-017-0029-9 (PMC5608949; doi:10.1038/s41540-017-0029-9)
Supplement: Supplementary file 1 — Supplementary Material [file 41540_2017_29_MOESM1_ESM.zip › Supplement_onlineVersion/SupportingInformation_S11_Module identification example code.docx]

Phenotype-driven identification of modules in a hierarchical map of multifluid metabolic correlations

*Kieu Trinh Do, Maik Pietzner, David Rasp, Nele Friedrich, Matthias Nauck, Thomas Kocher, Karsten Suhre, Dennis O. Mook-Kanamori, Gabi Kastenmüller, Jan Krumsiek*

**Supporting Information S11: Module identification example code**

Our proposed module identification approach, composed of the generation of the hierarchical map and the module search algorithm, is available as R implementation in Supporting Information S11. In the following, the execution of the provided R scripts will be demonstrated.

Importantly, since the informed consent given by SHIP study participants does not cover public data posting, the procedure will be applied on simulated data at metabolite and sub-pathway level. This dummy dataset was generated from the covariance matrices of the real data.

# Prerequisites

The following R-packages and corresponding dependencies are required:

- *data.table* (version 1.9.6 or newer)
- *magrittr*
- *GeneNet*
- *Hmisc*
- *graph*
- *igraph*
- *RCytoscape*

For automatic visualization, Cytoscape 2.8 with Plugin CytoscapeRPC (activated) is required to run in the background.

###### Source R scripts

source(file="Module_Identification.R")

silent=FALSE # set to TRUE to prevent any text output

# Metabolite level

###### Data import

The following variables will be imported:

- annotation_DT: A data.table containing annotations for the different metabolites with the columns ‘name’ containing the unique names of the variables, ‘label’ containing the labels of the variables for visualization, and ‘Sub.pathway’ and ‘Super.pathway’ containing the annotations of the metabolites (or any other groupings).
- metabolites_mat: A matrix (samples x variables) containing the (preprocessed) concentration levels of the metabolites for the different samples. Columns must be metabolites and rows must correspond to the samples.
- phenotype_DT: A data.table containing values for the phenotype for each sample. At metabolite level, we simulated the dummy phenotype such that it shows sparse associations (see main manuscript).
- covars_DT: A data.table containing the covariates for each sample, e.g., gender, age, and BMI.

Code for data import:

annotation_DT <- fread(input="simulated_data/annotations.txt")

metabolites_mat <- as.matrix(read.delim(file="simulated_data/datamat.txt", header=TRUE, sep='\t', check.names=FALSE, quote=""))

covars_DT <- fread(input="simulated_data/covars.txt")

phenotype_DT <- fread(input="simulated_data/phenotype_sparse.txt")

###### Network generation

A precalculated network can be imported from a file in edge-list format, and converted to an igraph object.

net_DT <- fread(input="simulated_data/network.txt")

net_graph <- graph_from_data_frame(d=net_DT, directed=FALSE, vertices=annotation_DT)

Alternatively, a Gaussian graphical model (GGM) can be directly estimated from the imported data. We recommend to correct the GGM for the covariates and the phenotype. For multiple testing correction, the significance level (alpha) and the method (correction.method) to be used must be specified. correction.method can be set to "holm", "hochberg", "hommel", "bonferroni", "BH", "BY", "fdr", or "none", see also p.adjust.

net_graph <- **createGGM**(metabolites=metabolites_mat, covars= **cbind**(covars_DT[,2:4], phenotype_DT$value), annotations=annotation_DT, alpha=0.05, correction.method="bonferroni")

###### Module identification

The phenotype-driven module identification is performed by the function identifyModule, for which the following parameters are required.

- graph: An igraph object, which was imported or generated in the previous step. The ID of the nodes must correspond to the ‘name’ column in annotations_DT.
- metabolites: This can be a data.table, where the column ‘name’ contains the IDs of the metabolites, the column ‘sampleID’ contains the ID of the observations, and ‘value’ contains the measurement of the metabolites. Alternatively, a matrix can be provided with columns corresponding to metabolites and rows corresponding to samples.
- phenotype: A vector with the values for a phenotype of interest. The entries must be in the same order as the rows in the metabolite matrix (same sample order).
- covars: A data.table with the covariates (see Data import above). Again, rows must be in the same order as for the metabolites.
- significance.level: The significance level (type 1 error) for the modules, default is 0.05.
- better.than.components: If TRUE, a module will only be extended and accepted if its score is higher than the scores of its components (even if it is not significant).
- merge.overlapping: If TRUE, in a consolidation step overlapping modules will be merged.
- for.sub.pathway: Must be set TRUE if modules at the sub-pathway level should be identified. In that case, metabolites data.table also needs a column named "Sub.pathway", which contains the corresponding sub-pathway annotations (see second example below).

mods <- identifyModules(graph=net_graph, metabolites=metabolites_mat,
 phenotype=phenotype_DT$value, covars=covars_DT[,2:4],
 alpha=0.05, better.than.components=TRUE, merge.overlapping=TRUE, for.sub.pathway=FALSE, silent=silent)

###### Save modules to file

writeModules(mods=mods, file.name="Results/Simulated_modules_levelMet.txt")

###### Network and module visualization via Cytoscape

Note that Cytoscape 2.8 with activated Plugin CytoscapeRPC is required to run in the background for the automatic visualization of the network and the modules.

drawModules(graph=net_graph, title=" Simulated_modules_levelMet", module_DT= mods$modules, node_DT=mods$vertices, silent=silent)

Results are in folder Results/.

## Sub-pathway level

###### Network generation

As described in the main manuscript, the sub-pathway network is generated by estimating a Gaussian graphical model on the pathway eigenmetabolites. This procedure is performed by the function createSubGGM.

subnet_graph <- createSubGGM(metabolites=metabolites_mat, covars=
 cbind(phenotype_DT$value, covars_DT[,2:4]), annotations=annotation_DT)

###### Module identification

For the identification of modules at sub-pathway level, we simulated a phenotype with dense associations.

phenotype_DT <- **fread**(input="simulated_data/phenotype_dense.txt")

The module identification for the phenotype with dense associations is again performed with function identifyModules.

submods <- **identifyModules**(graph=subnet_graph, metabolites= metabolites_mat, phenotype=phenotype_DT$value, annotations=annotation_DT, covars=covars_DT[,3:4], alpha=0.05, better.than.components=TRUE, merge.overlapping=TRUE, for.sub.pathway=TRUE, silent=silent)

###### Save and visualize network and modules via Cytoscape

Note that Cytoscape 2.8 with activated Plugin CytoscapeRPC is required to run in the background for the automatic visualization of the network and the modules.

writeModules(mods=submods, file.name="Results/Simulated_modules_levelSub.txt")

drawModules(graph=subnet_graph, title="Simulated_modules_levelSub", module_DT= submods$modules, node_DT= submods$vertices, silent=silent)

Results are in folder Results/.
