# Supplementary material for: Phenotype-driven identification of modules in a hierarchical map of multifluid metabolic correlations
Source: NPJ Syst Biol Appl. 2017 Sep 21;3:28. doi: 10.1038/s41540-017-0029-9 (PMC5608949; doi:10.1038/s41540-017-0029-9)

Phenotype-driven identification of modules in a hierarchical map of multifluid metabolic correlations

*Kieu Trinh Do, Maik Pietzner, David Rasp, Nele Friedrich, Matthias Nauck, Thomas Kocher, Karsten Suhre, Dennis O. Mook-Kanamori, Gabi Kastenmüller, Jan Krumsiek*

**Supporting Information S2: Explained variance of SHIP-TREND sub- and super-pathways**

To enable the construction of a pathway-based correlation network, a single representative value per sample was defined as the first principal component of the metabolite concentrations in each sub-pathway (termed sub-pathway *eigenmetabolite*). 22% of the plasma, 34% of the urine, and 33% of the saliva sub-pathways comprise only one metabolite, and thus their representative value is equivalent to the concentration of that one metabolite. For the remaining sub-pathways, the *eigenmetabolite* explains more than 40% of the total sub-pathway variance in the majority of cases (69%, 57%, and 80% of plasma, urine, and saliva sub-pathways, respectively, Figure S2)**.** This rather high degree of explained variance indicates that the predefined pathways mostly represent homogeneous groups of metabolites, and thus *eigenmetabolites* are reasonable statistical representatives of the pathways. Urine showed a lower number of *eigenmetabolites* explaining more than 40% variance than the other two fluids, suggesting a higher fraction of heterogeneous sub-pathways.

For super-pathways, we refrained from estimating a super-pathway GGM based on the super-pathway *eigenmetabolites* due to very high heterogeneity in some super-pathways. Only ‘Carbohydrate [plasma]’, ‘Carbohydrate [saliva]’, ‘Energy [saliva]’, and ‘Energy [urine]’ showed an explained variance of >40% in the first PC (Figure S2).


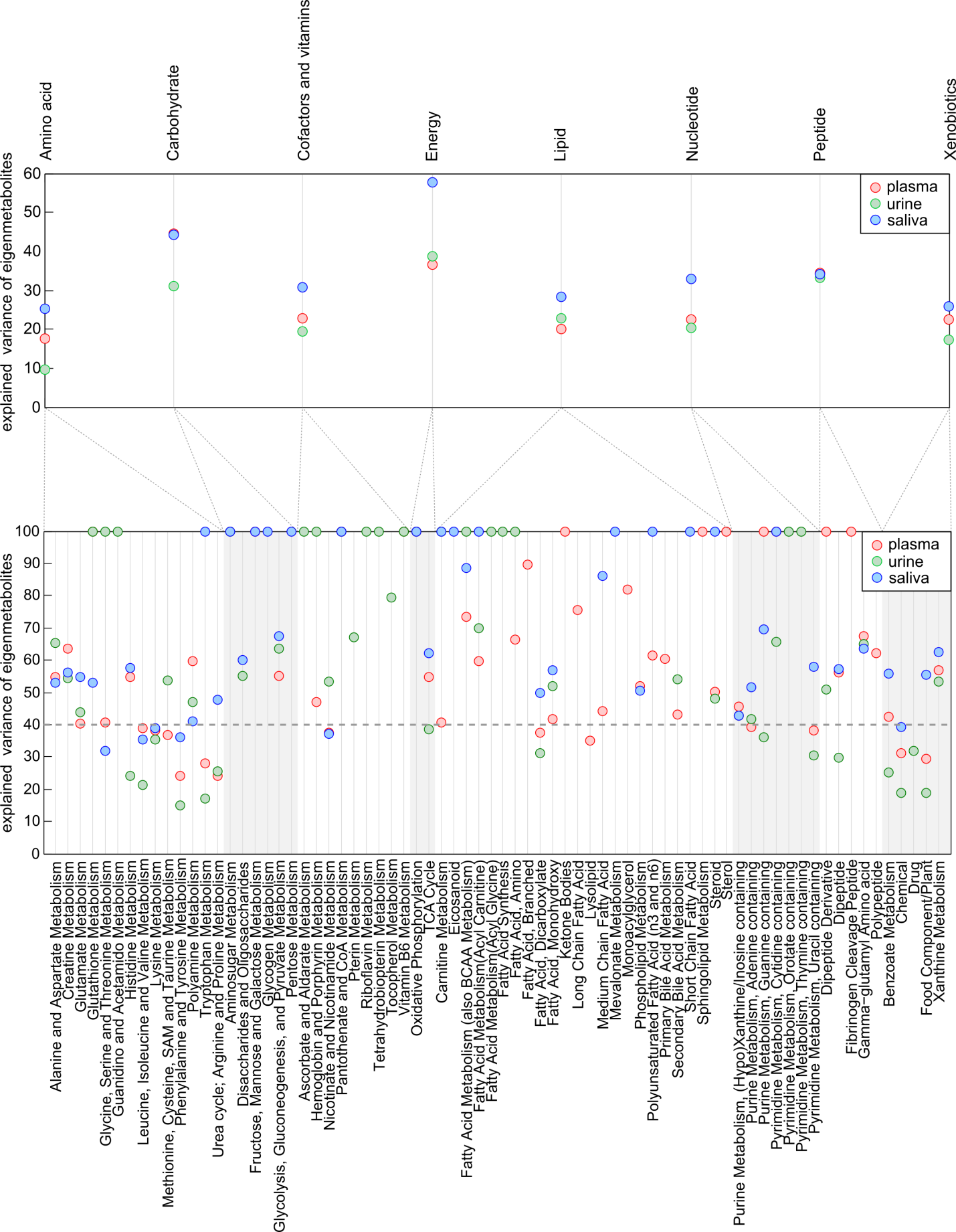


Figure S2. Explained variance of super- (top) and sub-pathway (bottom) *eigenmetabolites*.

The following figures depict the cumulative explained variances of all principal components for each sub- and super-pathway in plasma (red), urine (green), and saliva (blue).

Sub-pathways


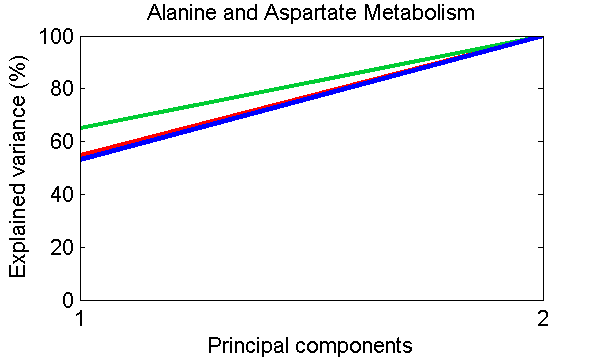

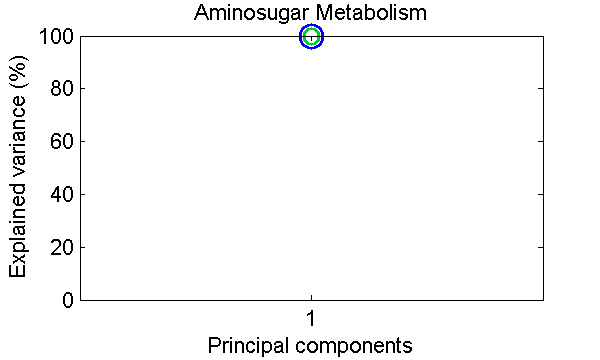

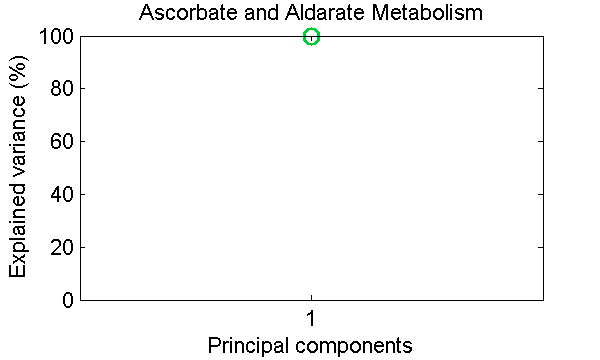

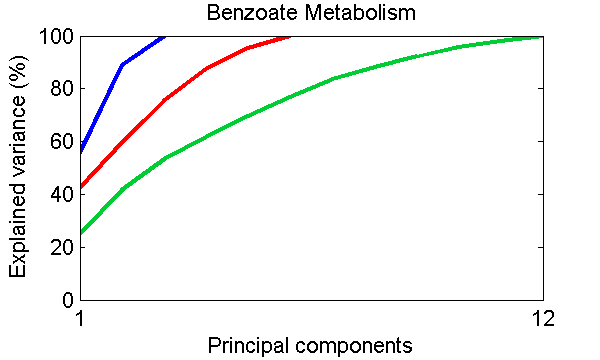

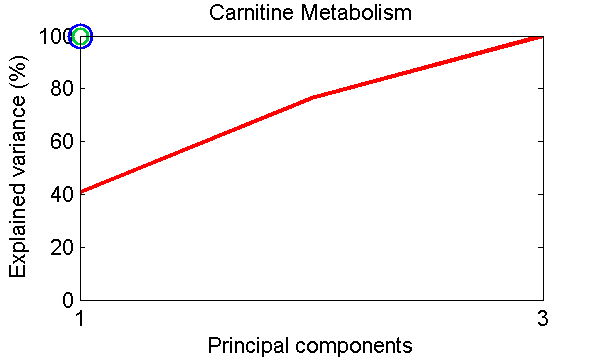

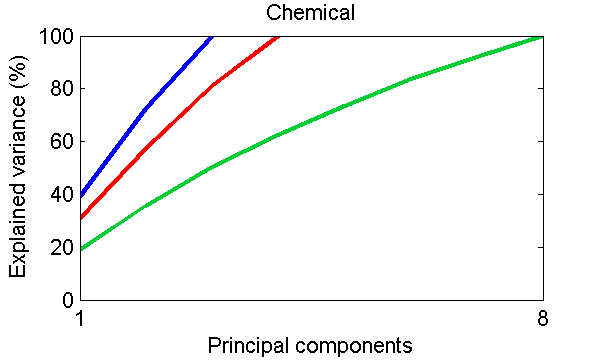

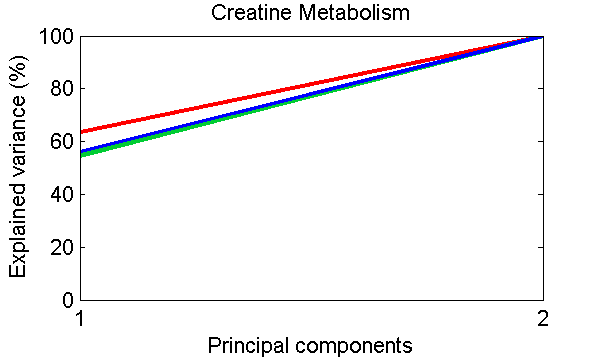

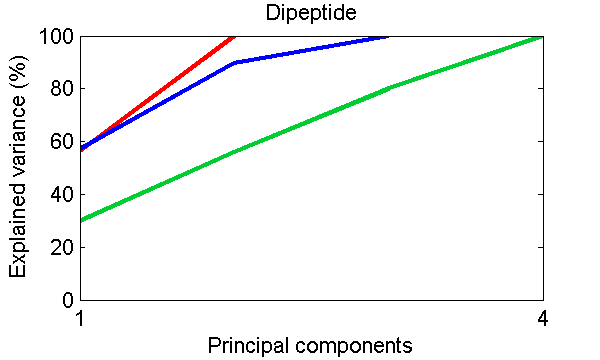

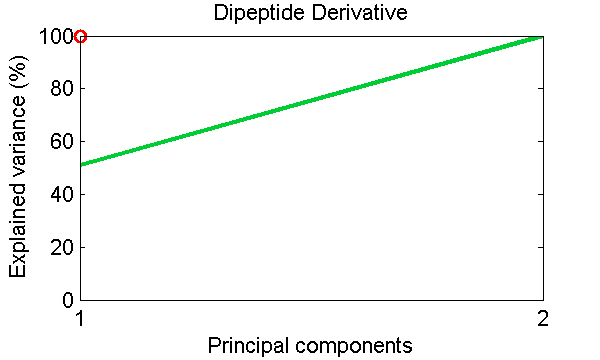

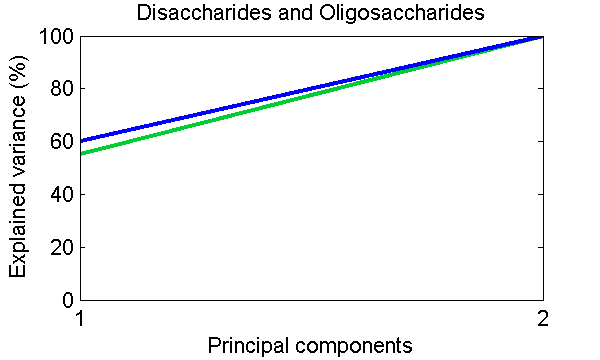

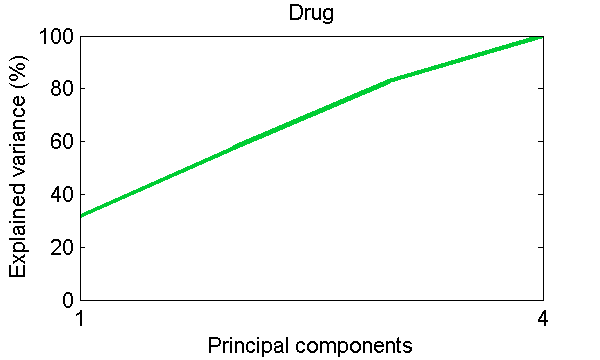

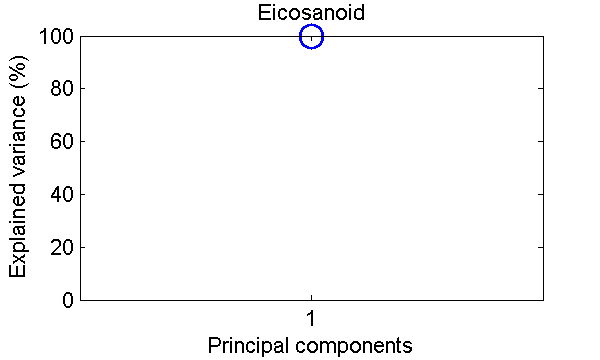

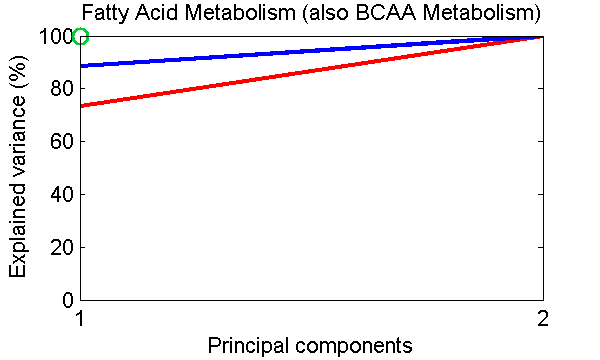

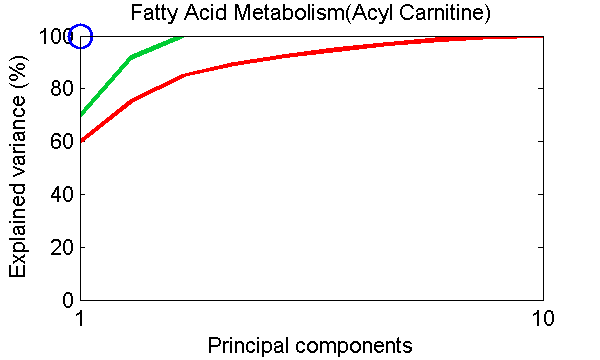

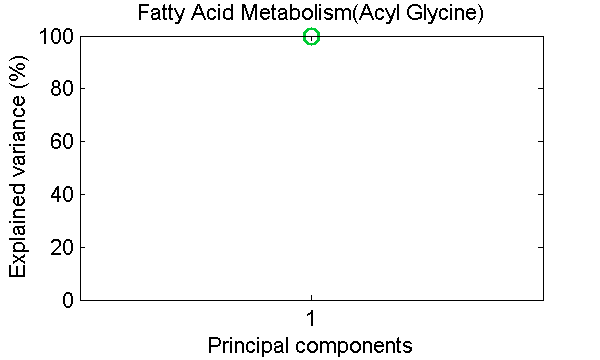

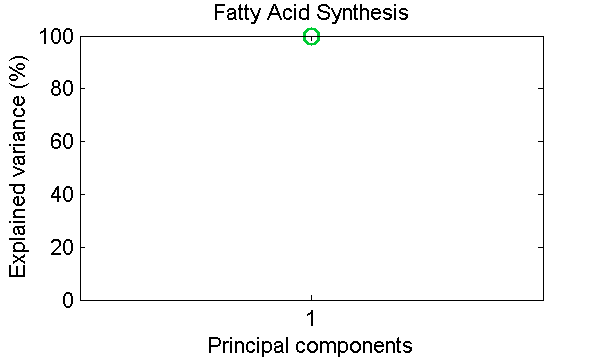

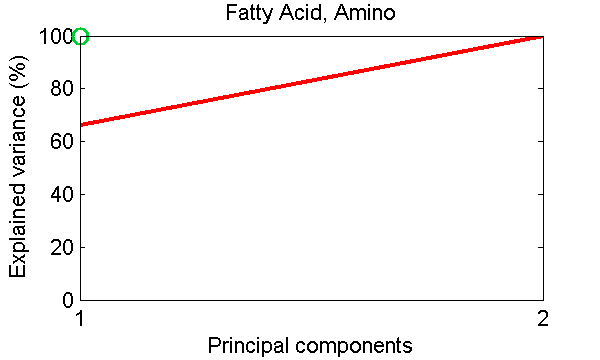

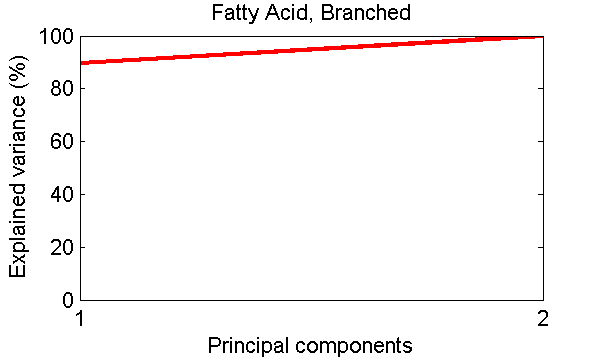

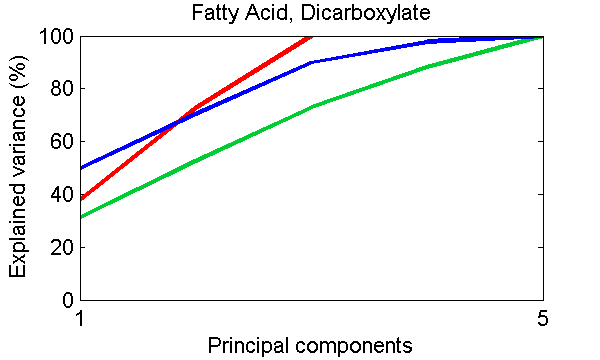

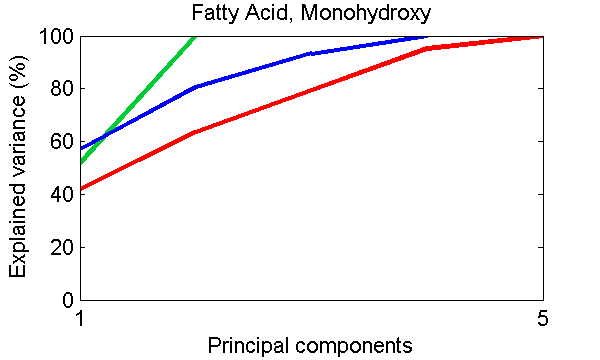

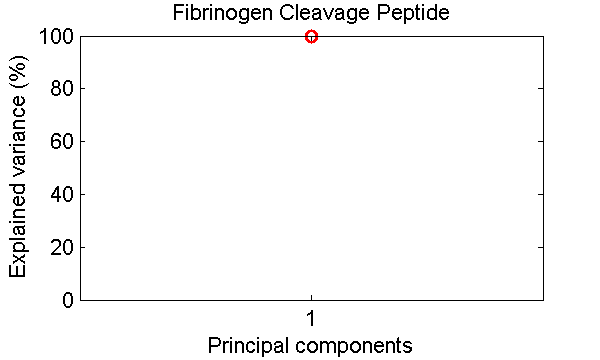

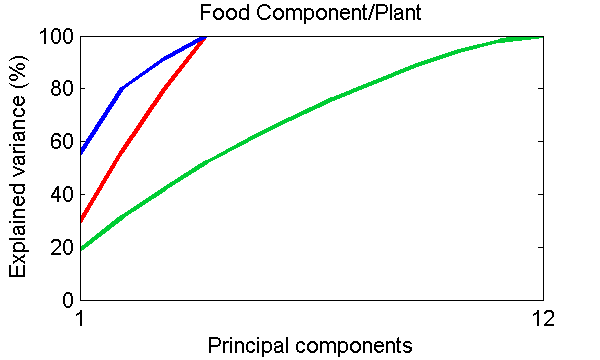

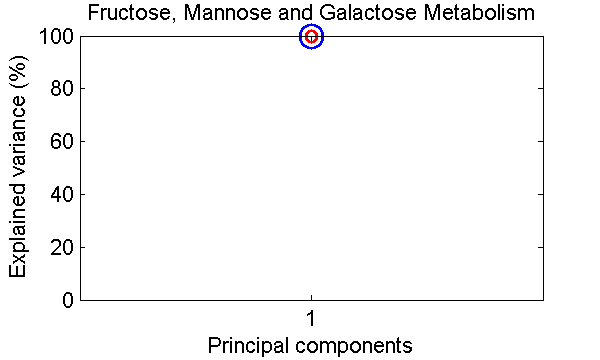

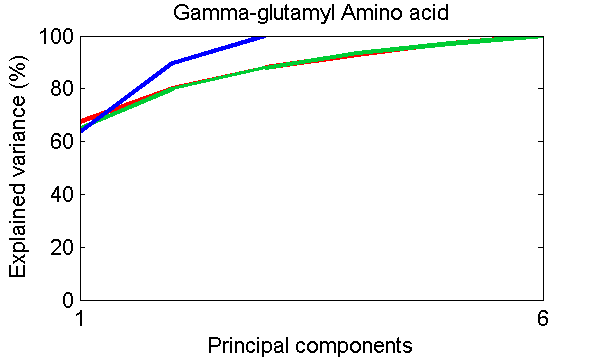

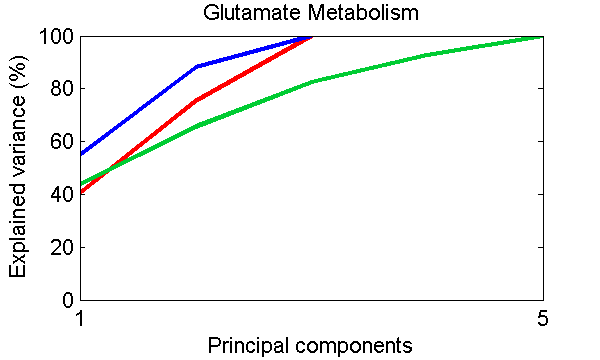

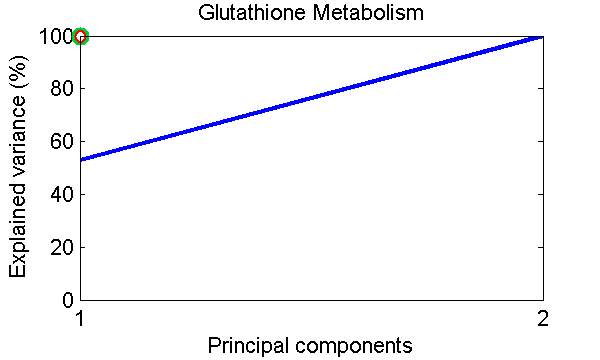

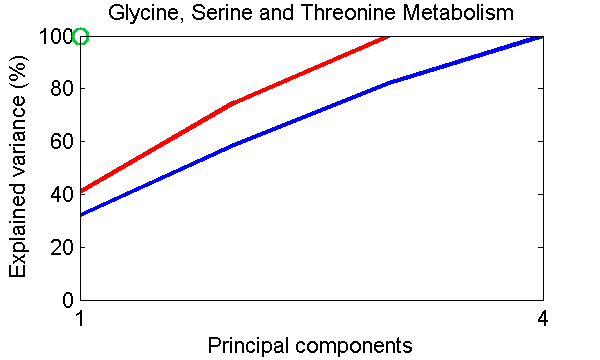

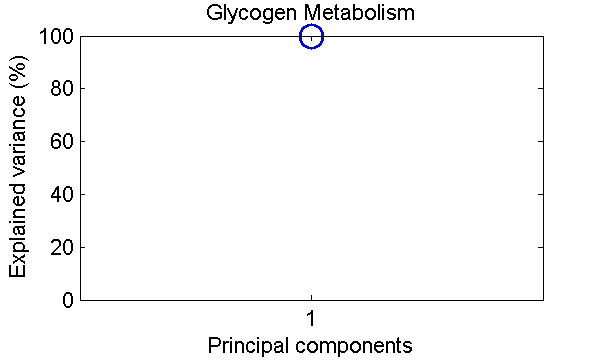

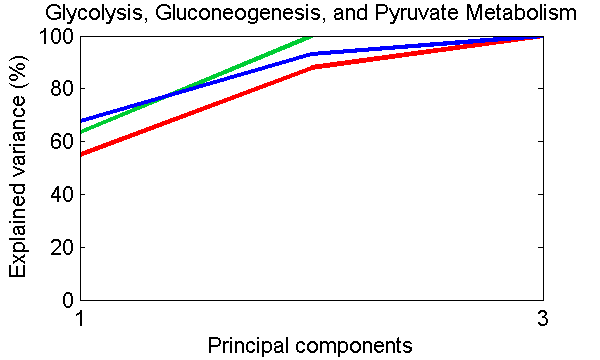

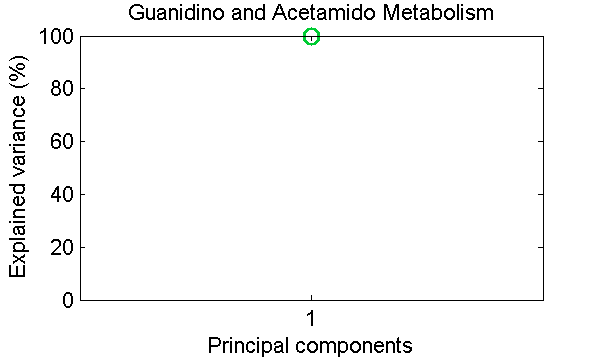

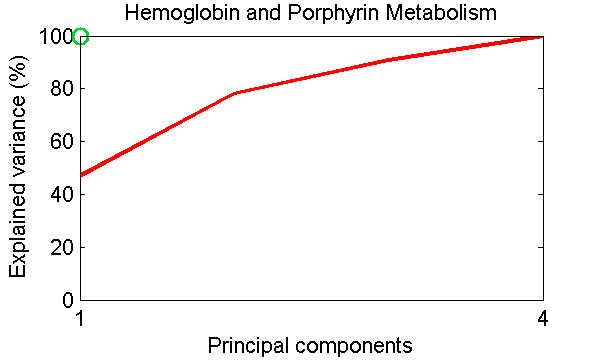

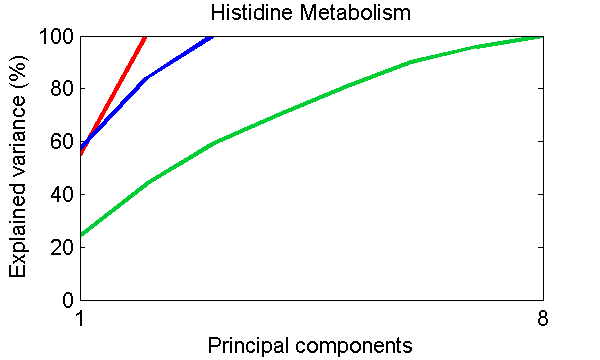

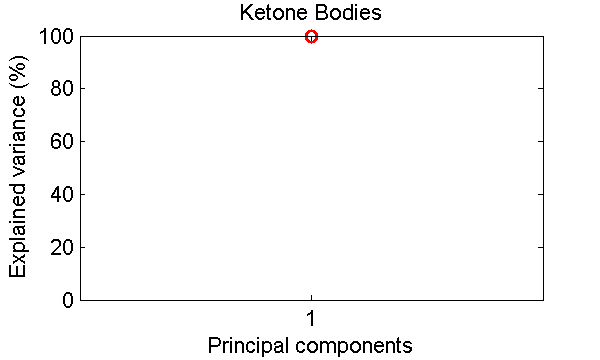

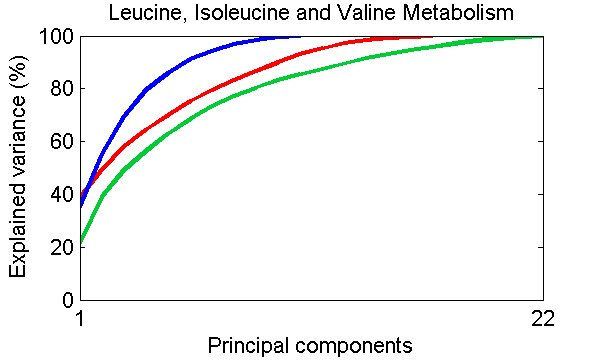

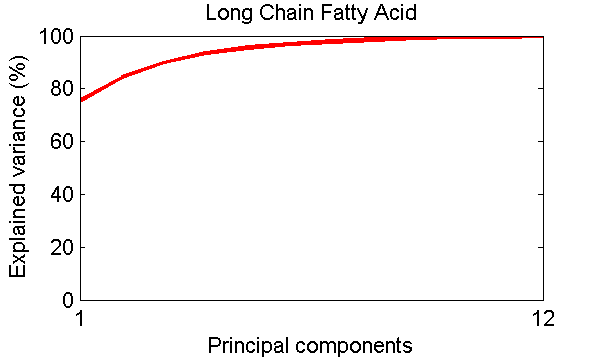

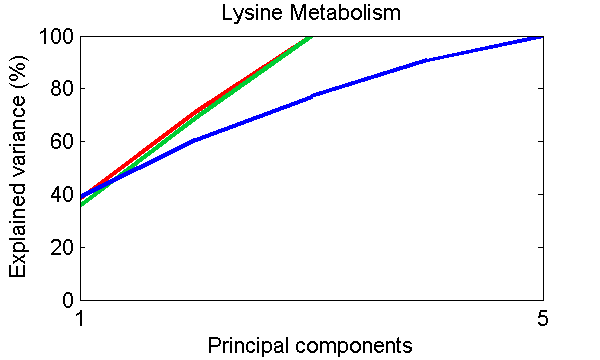

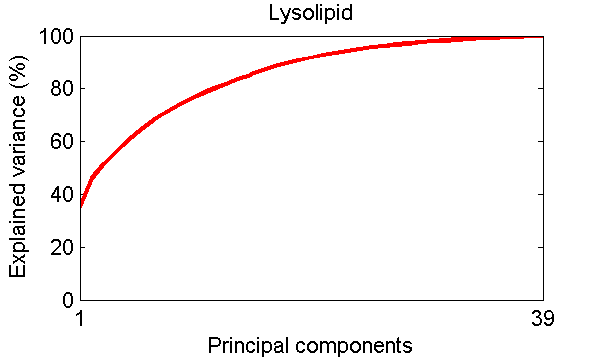

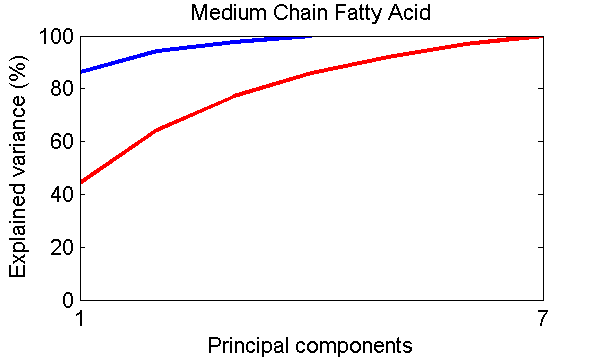

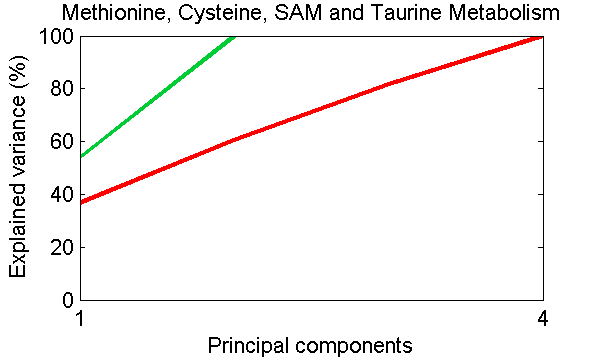

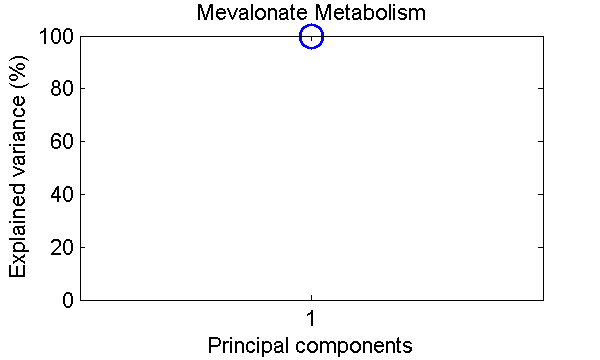

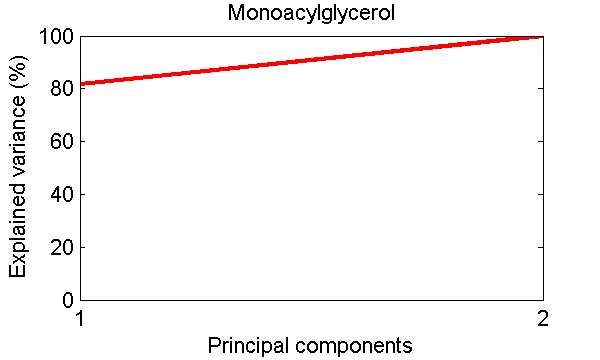

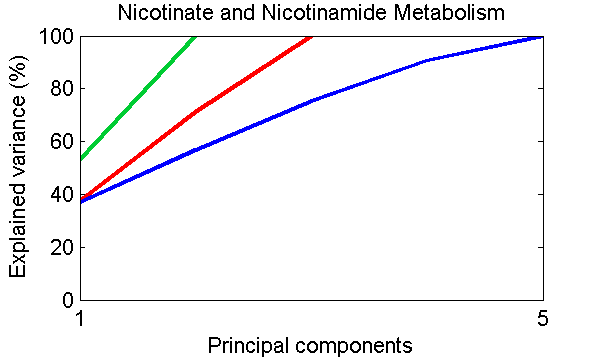

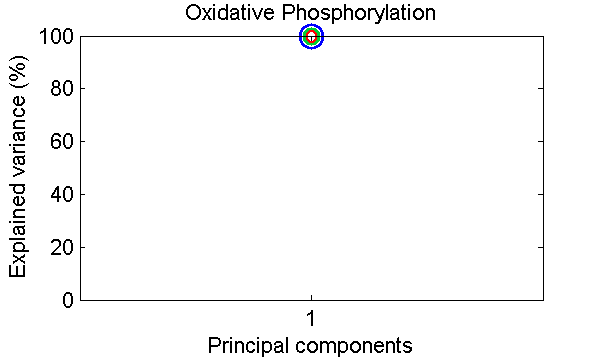

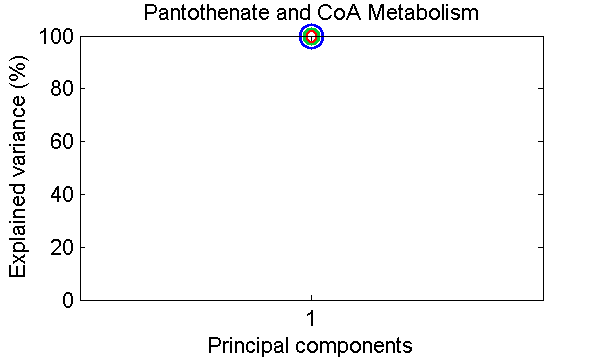

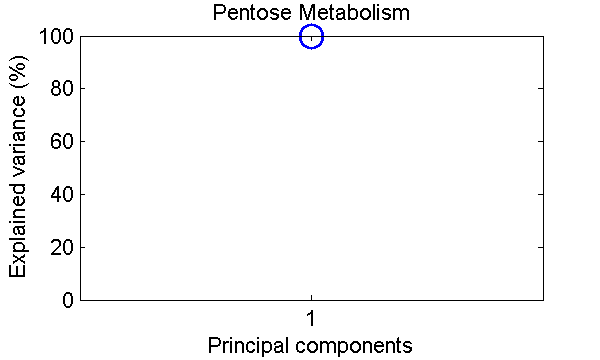

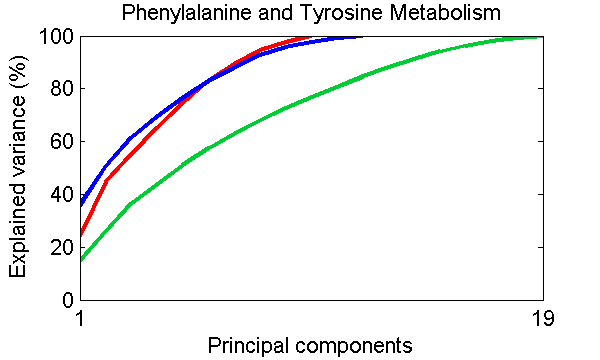

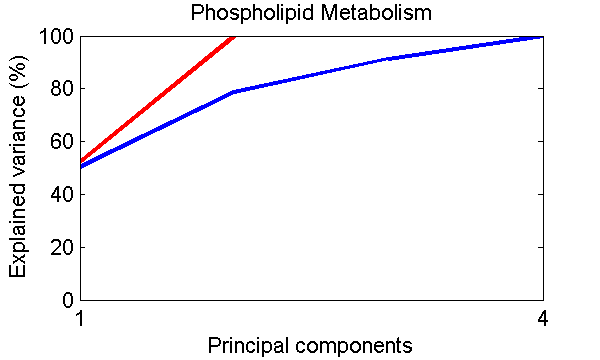

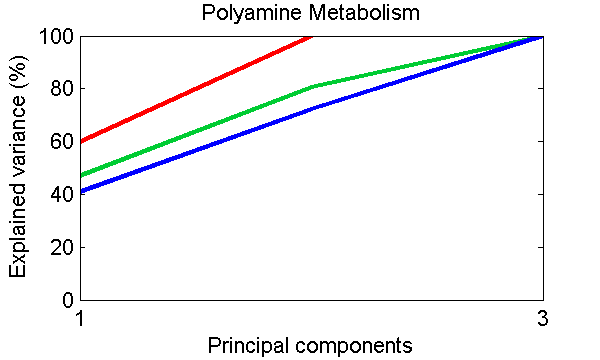

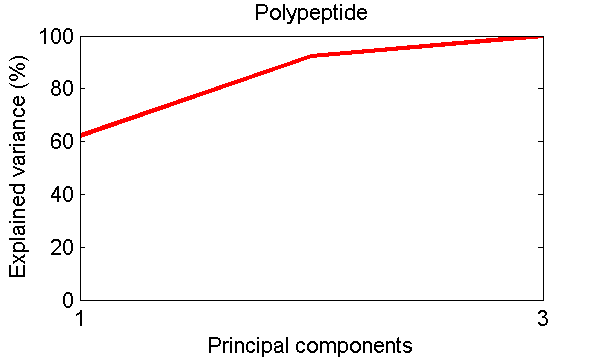

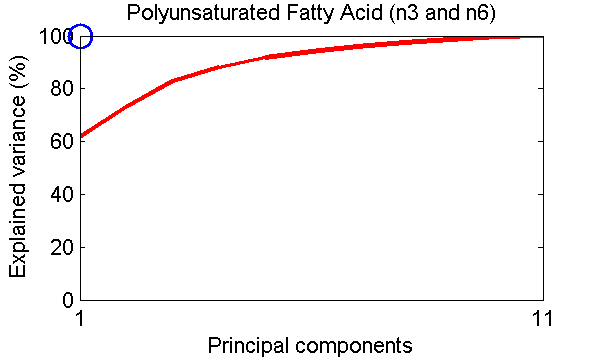

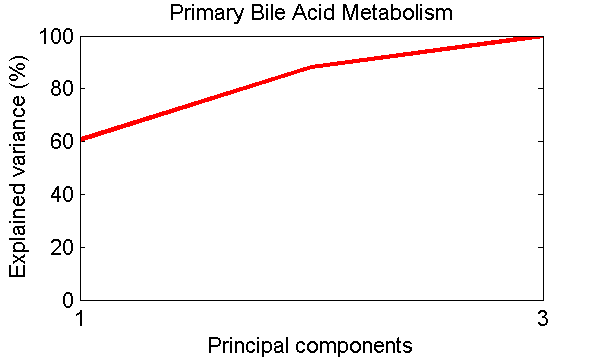

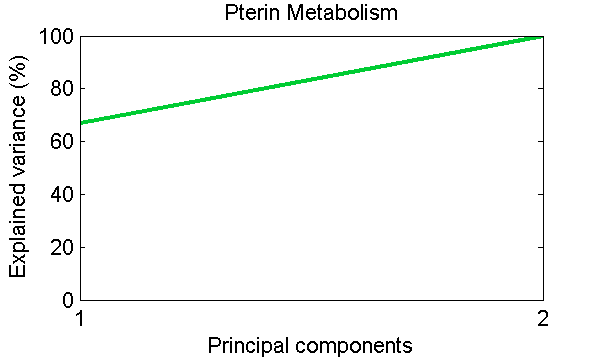

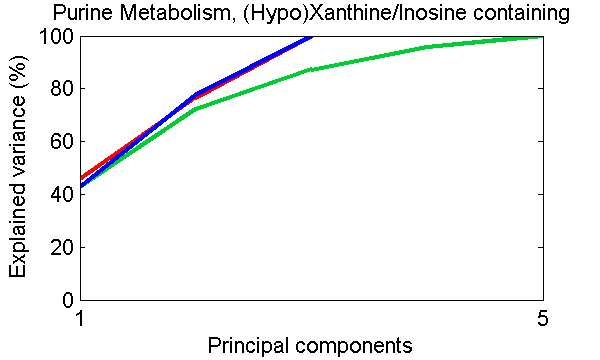

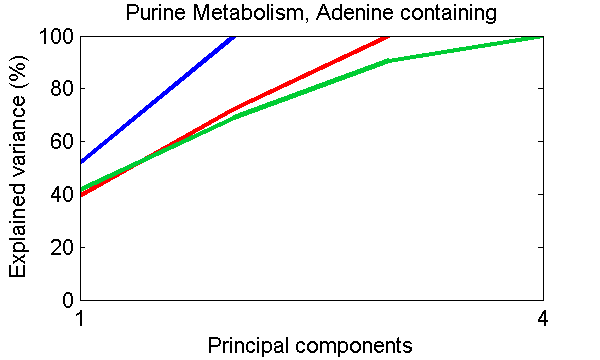

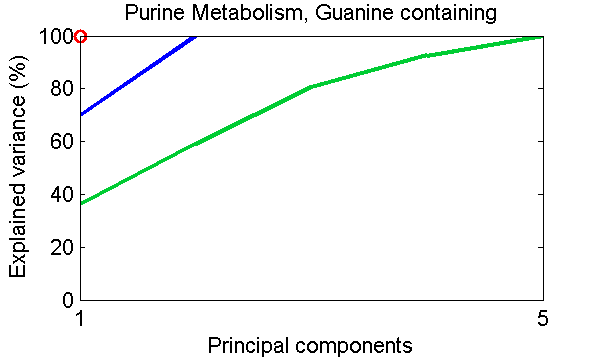

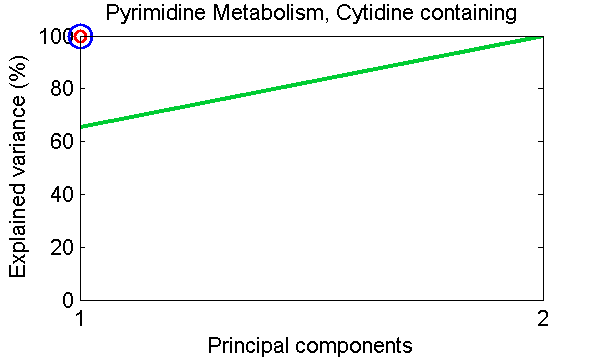

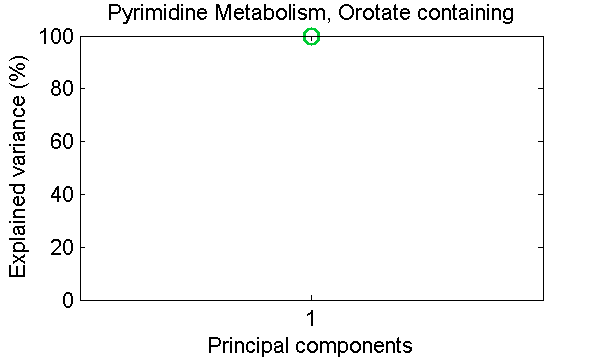

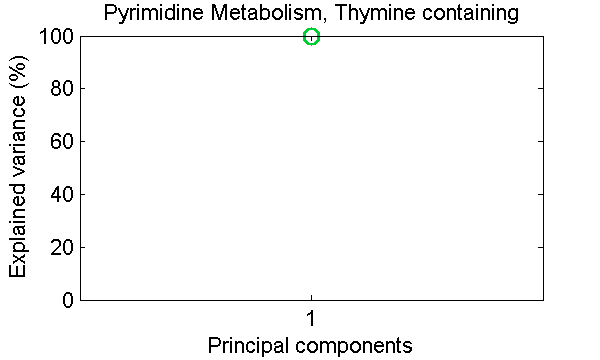

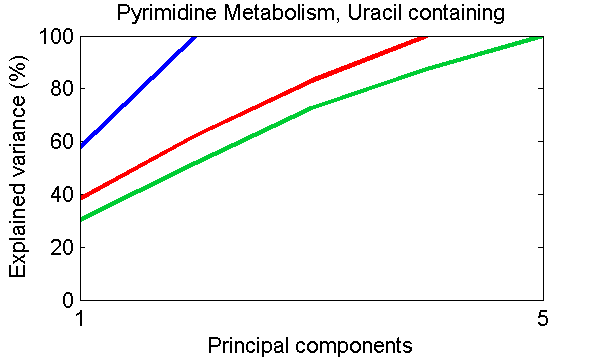

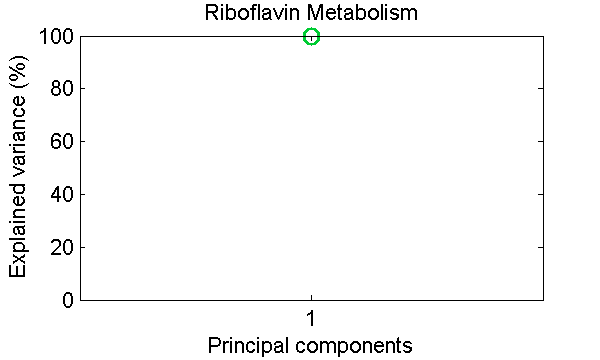

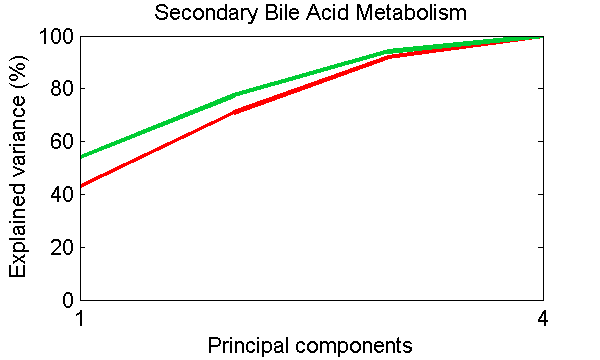

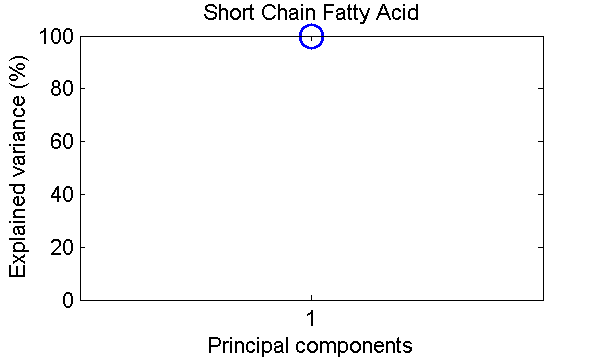

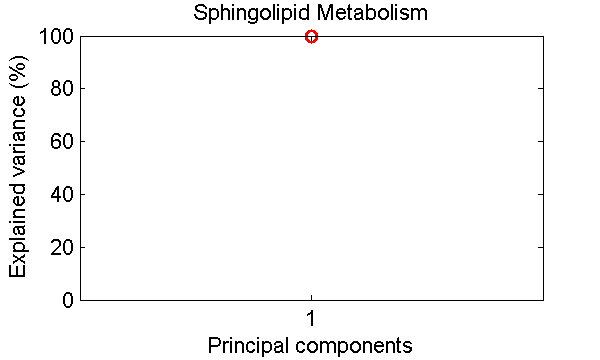

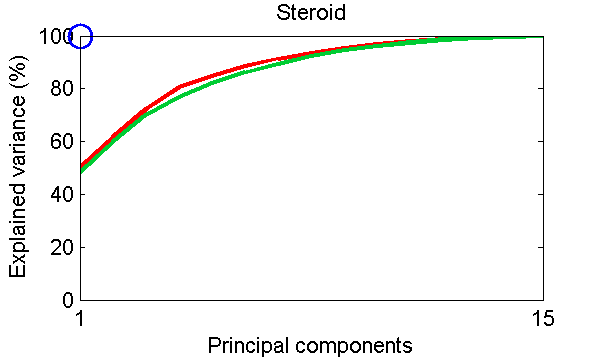

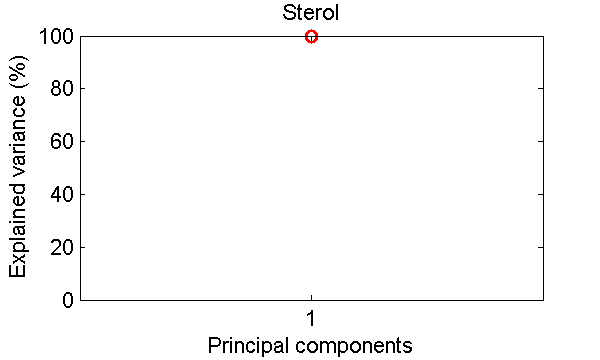

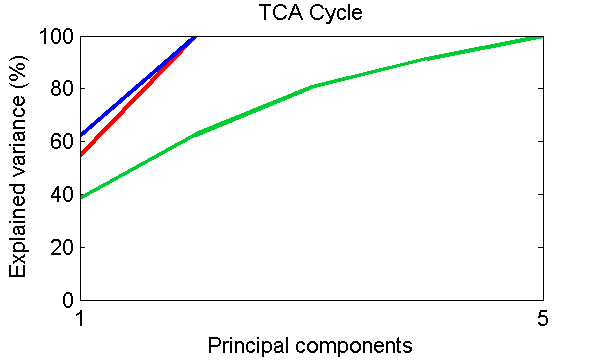

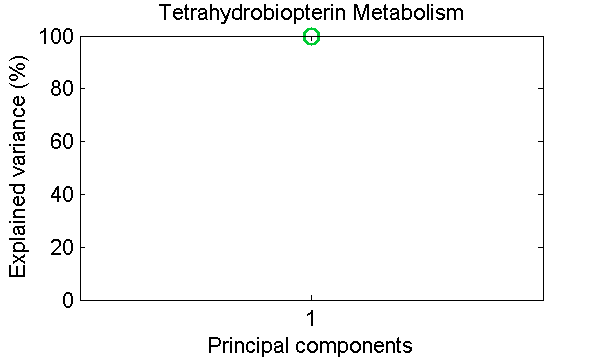

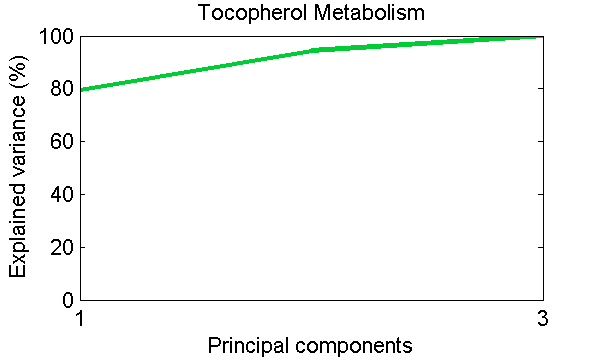

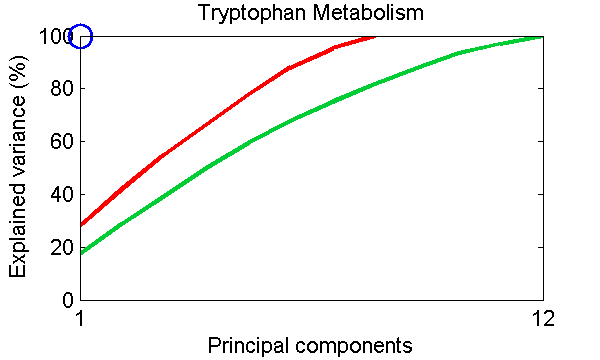

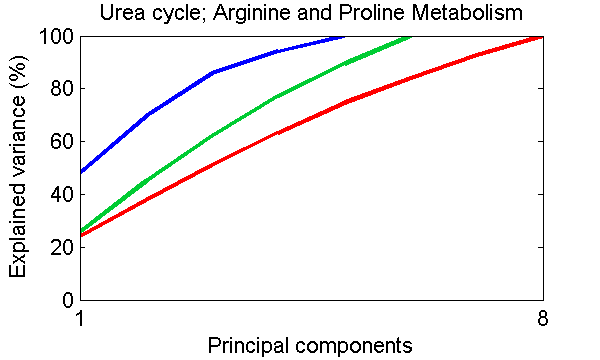

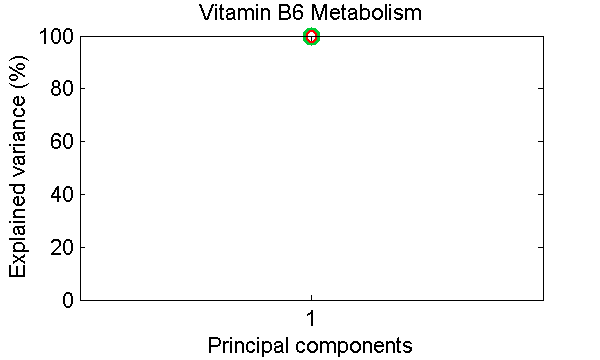

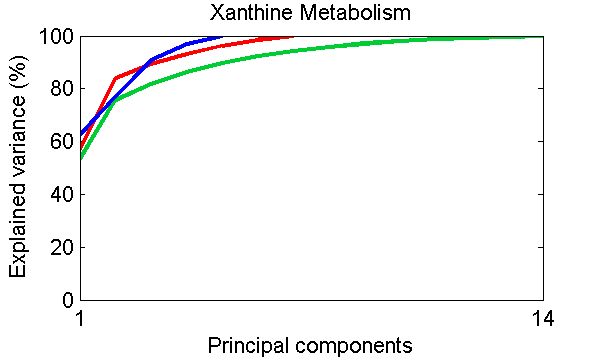


Super-pathways


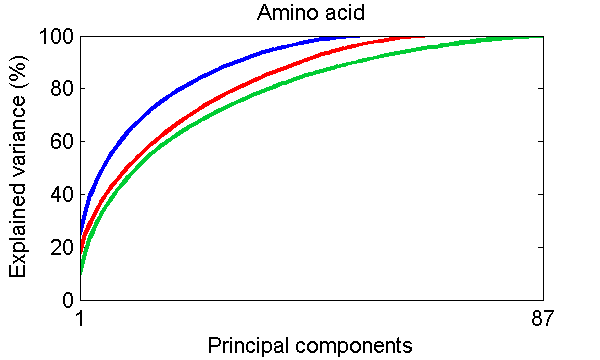

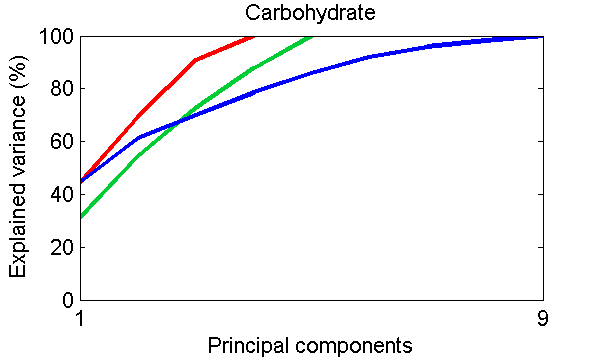

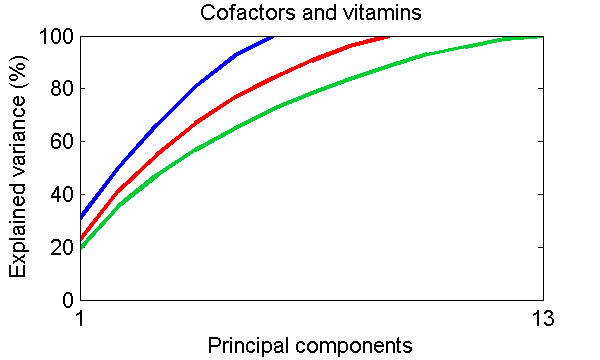


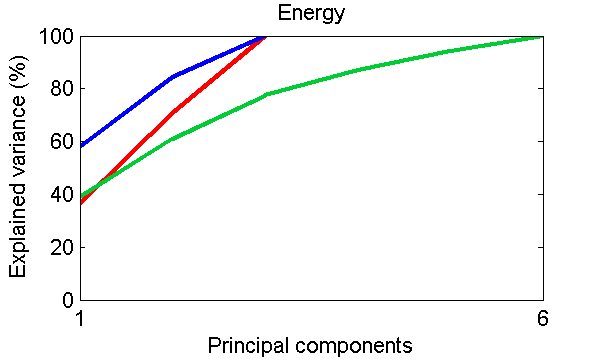

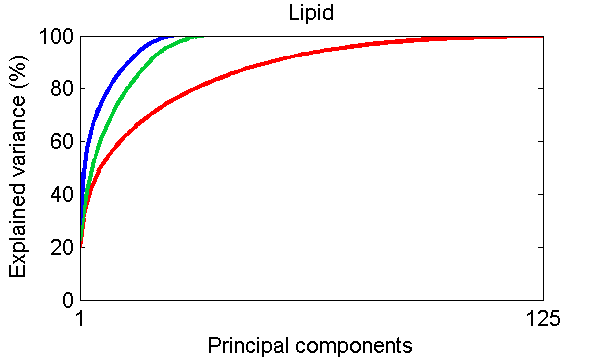

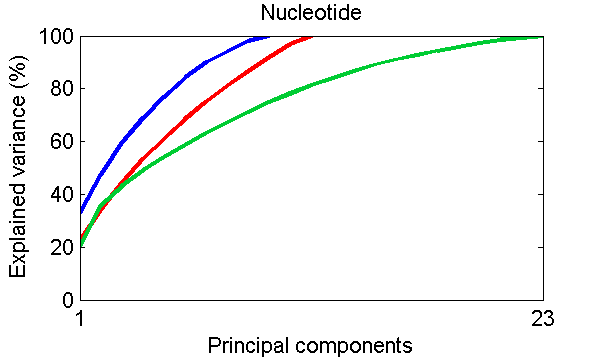

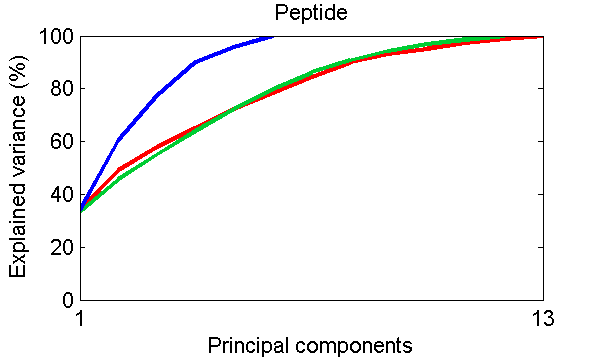

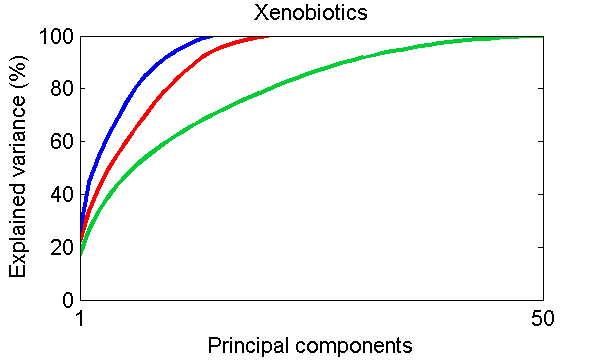

Supplement: Supplementary file 1 — Supplementary Material [file 41540_2017_29_MOESM1_ESM.zip › Supplement_onlineVersion/SupportingInformation_S2_Explained variance of pathways.docx]
