# Supplementary material for: Phenotype-driven identification of modules in a hierarchical map of multifluid metabolic correlations
Source: NPJ Syst Biol Appl. 2017 Sep 21;3:28. doi: 10.1038/s41540-017-0029-9 (PMC5608949; doi:10.1038/s41540-017-0029-9)
Supplement: Supplementary file 1 — Supplementary Material [file 41540_2017_29_MOESM1_ESM.zip › Supplement_onlineVersion/SupportingInformation_S2_Explained variance of pathways.pdf]

Phenotype-driven identification of modules in a hierarchical map of multifluid metabolic correlations

Kieu Trinh Do, Maik Pietzner, David Rasp, Nele Friedrich, Matthias Nauck, Thomas Kocher, Karsten Suhre, Dennis O. Mook-Kanamori, Gabi Kastenmüller, Jan Krumsiek

Supporting Information S2: Explained variance of SHIP-TREND sub- and super-pathways

To enable the construction of a pathway-based correlation network, a single representative value per sample was defined as the first principal component of the metabolite concentrations in each sub-pathway (termed sub-pathway *eigenmetabolite*). 22% of the plasma, 34% of the urine, and 33% of the saliva sub-pathways comprise only one metabolite, and thus their representative value is equivalent to the concentration of that one metabolite. For the remaining sub-pathways, the *eigenmetabolite* explains more than 40% of the total sub-pathway variance in the majority of cases (69%, 57%, and 80% of plasma, urine, and saliva sub-pathways, respectively, Figure S2)**.** This rather high degree of explained variance indicates that the predefined pathways mostly represent homogeneous groups of metabolites, and thus *eigenmetabolites* are reasonable statistical representatives of the pathways. Urine showed a lower number of *eigenmetabolites* explaining more than 40% variance than the other two fluids, suggesting a higher fraction of heterogeneous sub-pathways.

For super-pathways, we refrained from estimating a super-pathway GGM based on the super-pathway *eigenmetabolites* due to very high heterogeneity in some super-pathways. Only 'Carbohydrate [plasma]', 'Carbohydrate [saliva]', 'Energy [saliva]', and 'Energy [urine]' showed an explained variance of >40% in the first PC (Figure S2).

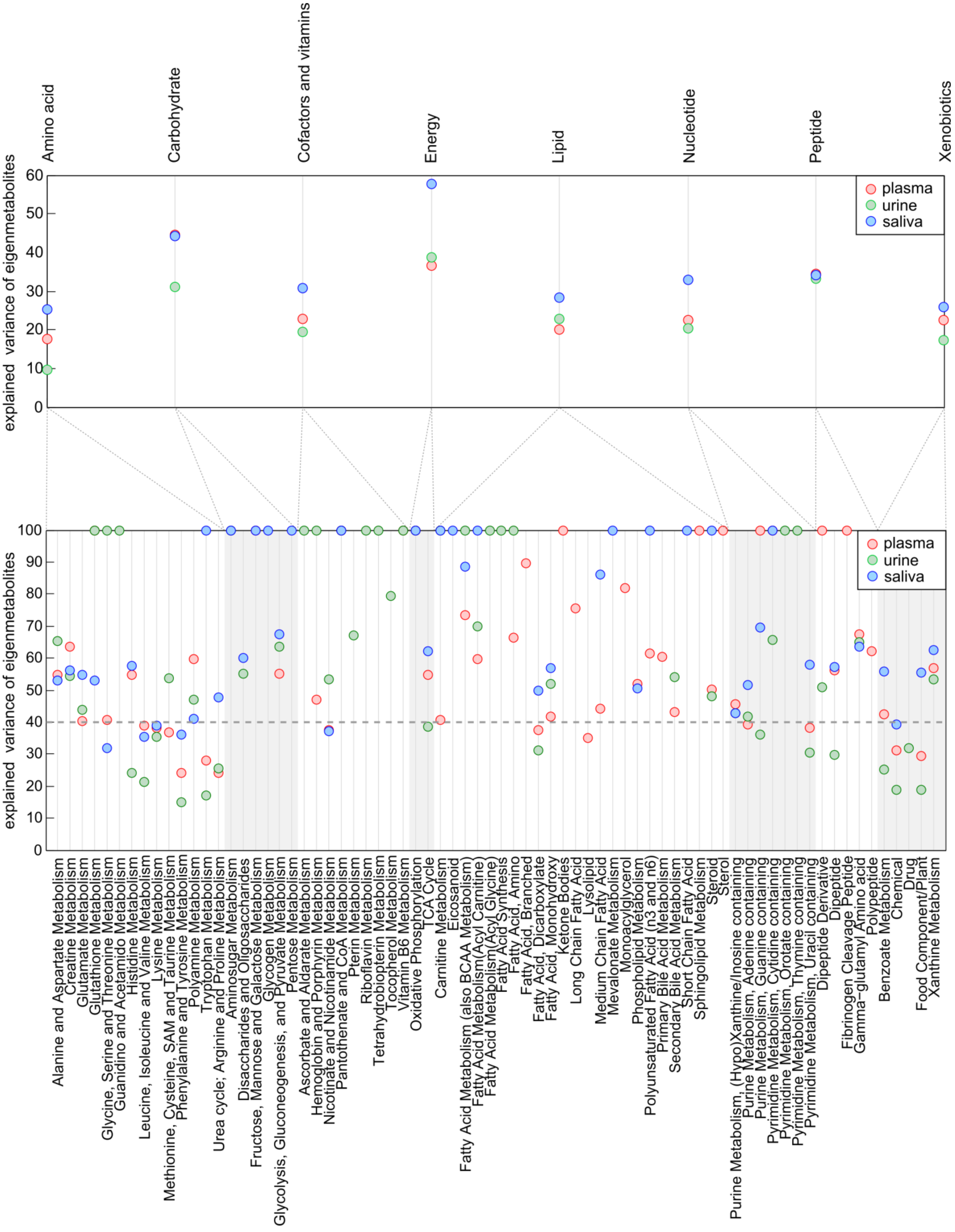

Figure S2. Explained variance of super- (top) and sub-pathway (bottom) *eigenmetabolites*.

The following figures depict the cumulative explained variances of all principal components for each sub- and super-pathway in plasma (red), urine (green), and saliva (blue).

Sub-pathways

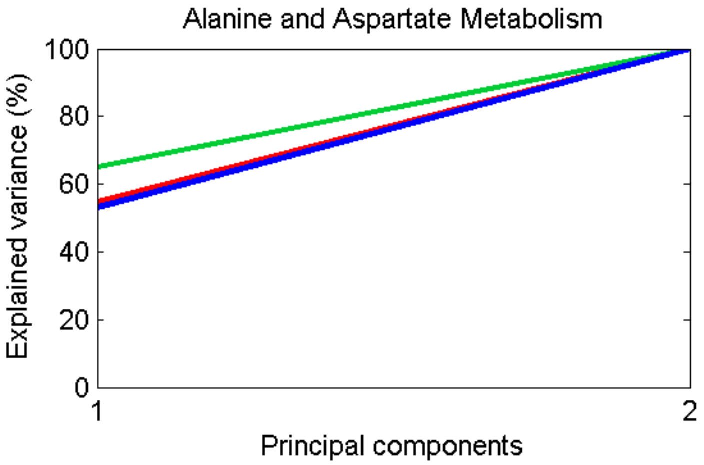

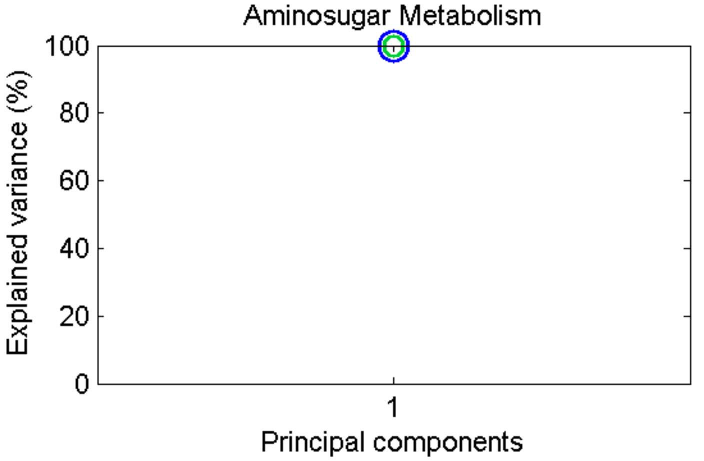

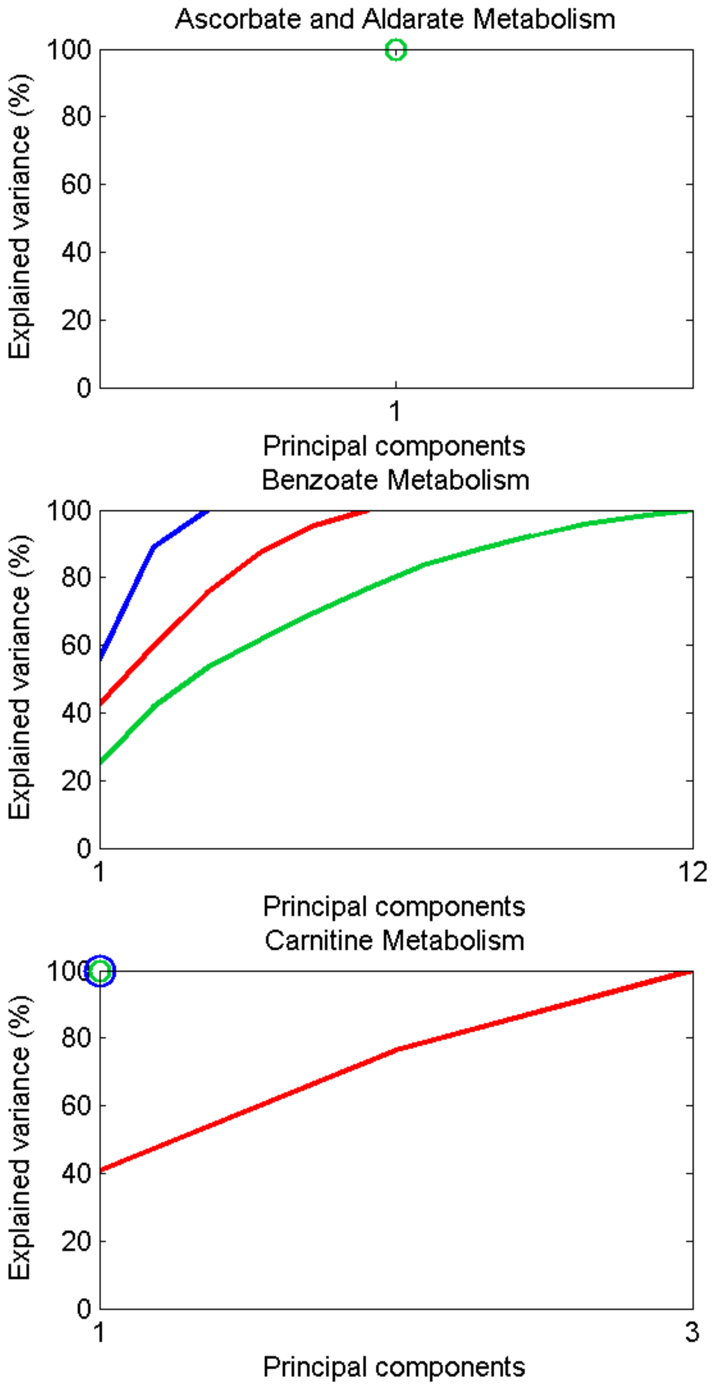
Ascorbate and Aldarate Metabolism
Explained variance (%)
100
80
60
40
20
0
1
Principal components
Benzoate Metabolism
Explained variance (%)
100
80
60
40
20
0
1
12
Principal components
Carnitine Metabolism
Explained variance (%)
100
80
60
40
20
0
1
3
Principal components

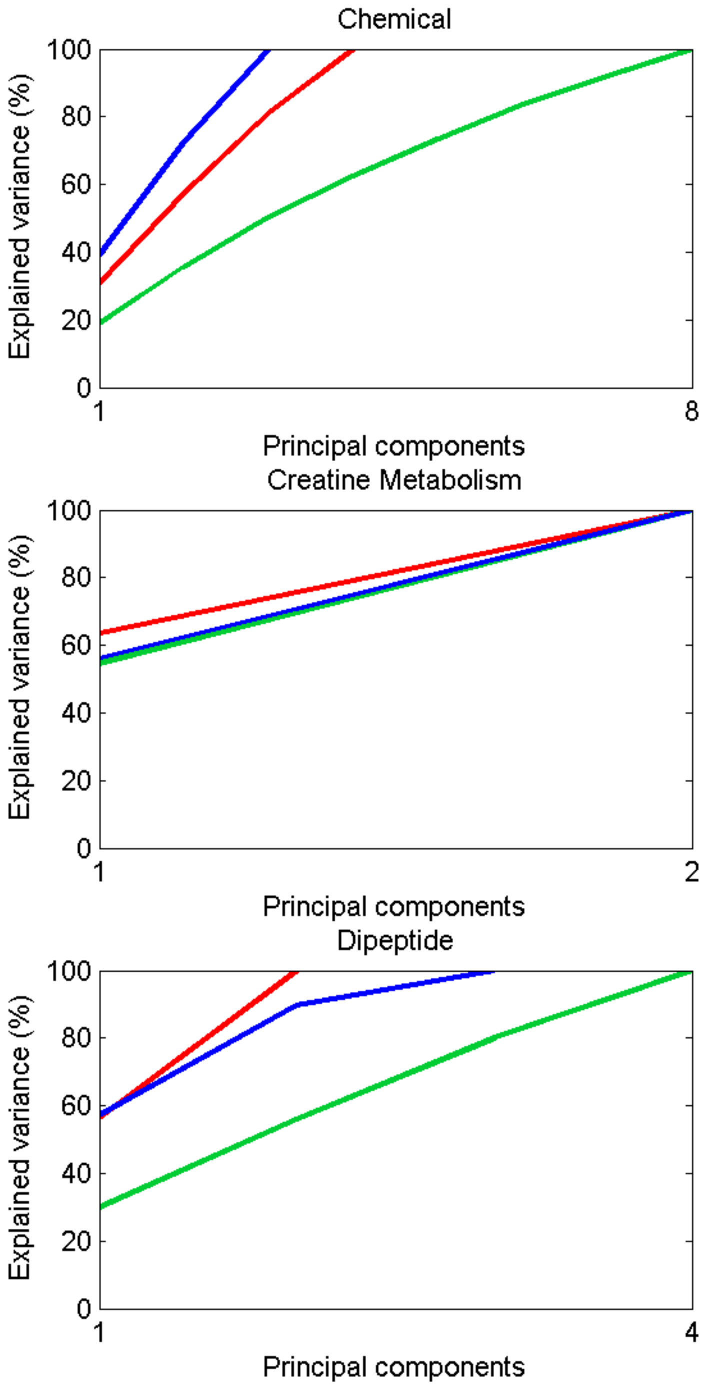
Chemical
Explained variance (%)
100
80
60
40
20
0
1
8
Principal components
Creatine Metabolism
Explained variance (%)
100
80
60
40
20
0
1
2
Principal components
Dipeptide
Explained variance (%)
100
80
60
40
20
0
1
4
Principal components

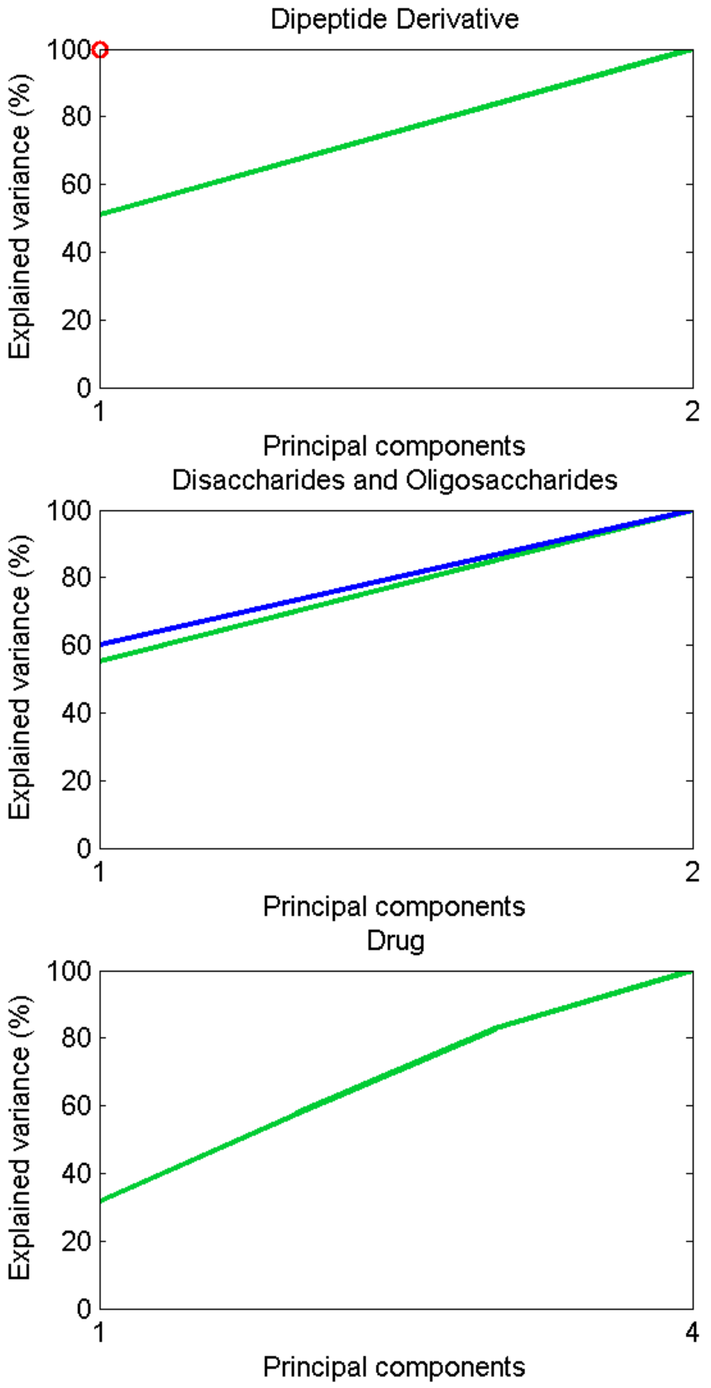
Dipeptide Derivative
Explained variance (%)
100
80
60
40
20
0
1
2
Principal components
Disaccharides and Oligosaccharides
Explained variance (%)
100
80
60
40
20
0
1
2
Principal components
Drug
Explained variance (%)
100
80
60
40
20
0
1
4
Principal components

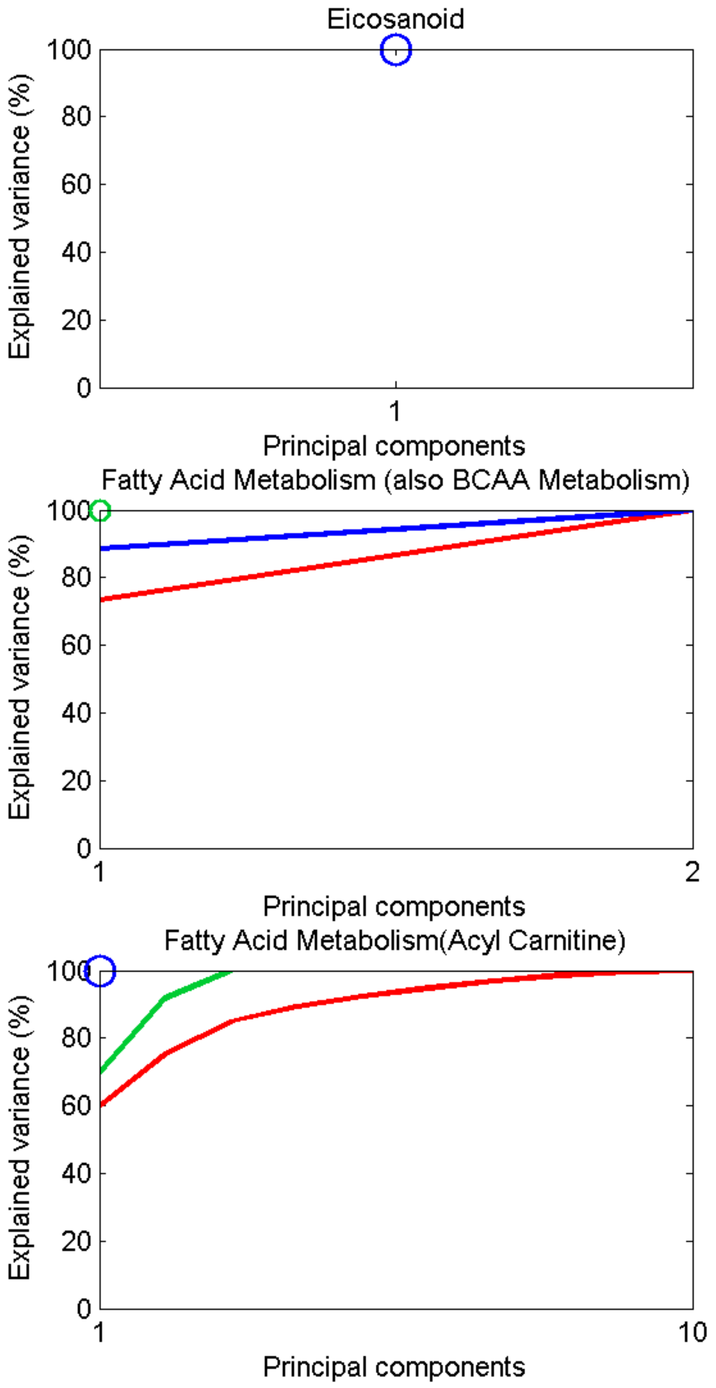
Eicosanoid
Explained variance (%)
100
80
60
40
20
0
1
Principal components
Fatty Acid Metabolism (also BCAA Metabolism)
Explained variance (%)
100
80
60
40
20
0
1
2
Principal components
Fatty Acid Metabolism(Acyl Carnitine)
Explained variance (%)
100
80
60
40
20
0
1
10
Principal components

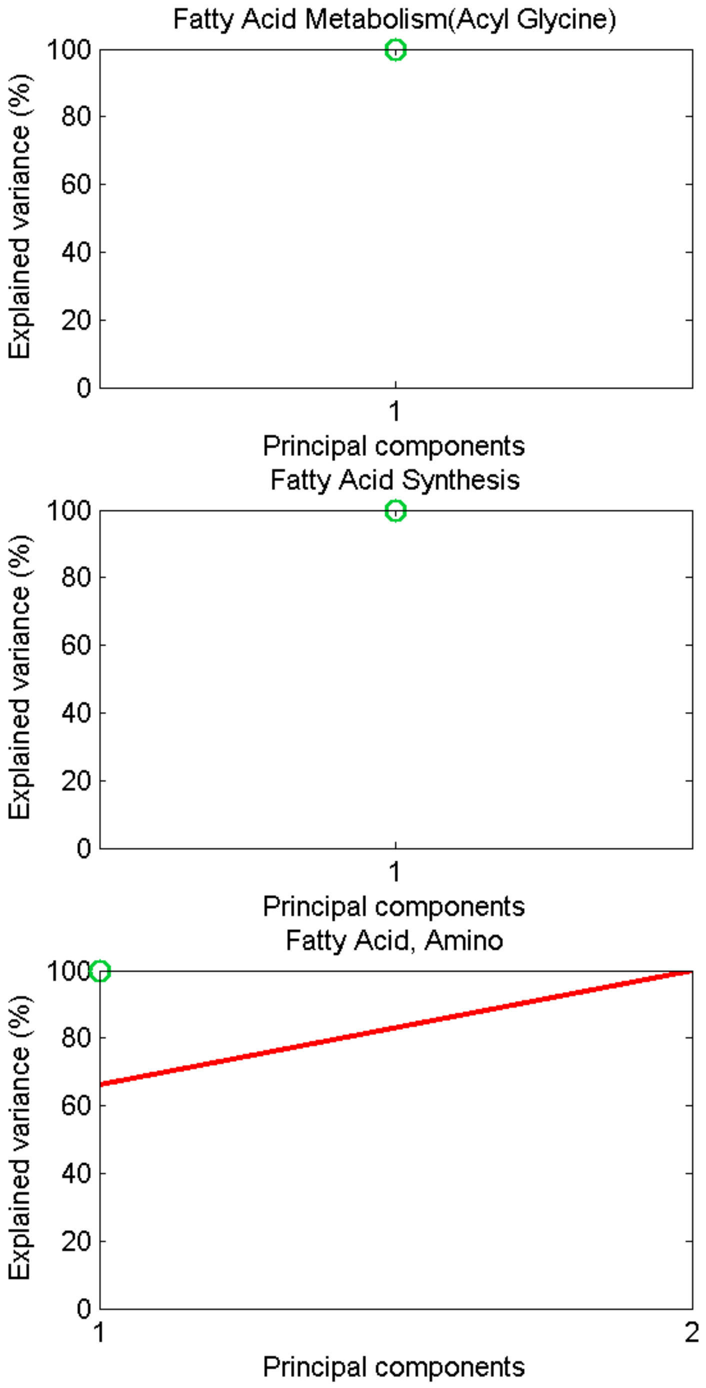

Fatty Acid Metabolism(Acyl Glycine)
Explained variance (%)
100
80
60
40
20
0
1
Principal components
Fatty Acid Synthesis
Explained variance (%)
100
80
60
40
20
0
1
Principal components
Fatty Acid, Amino
Explained variance (%)
100
80
60
40
20
0
1
2
Principal components

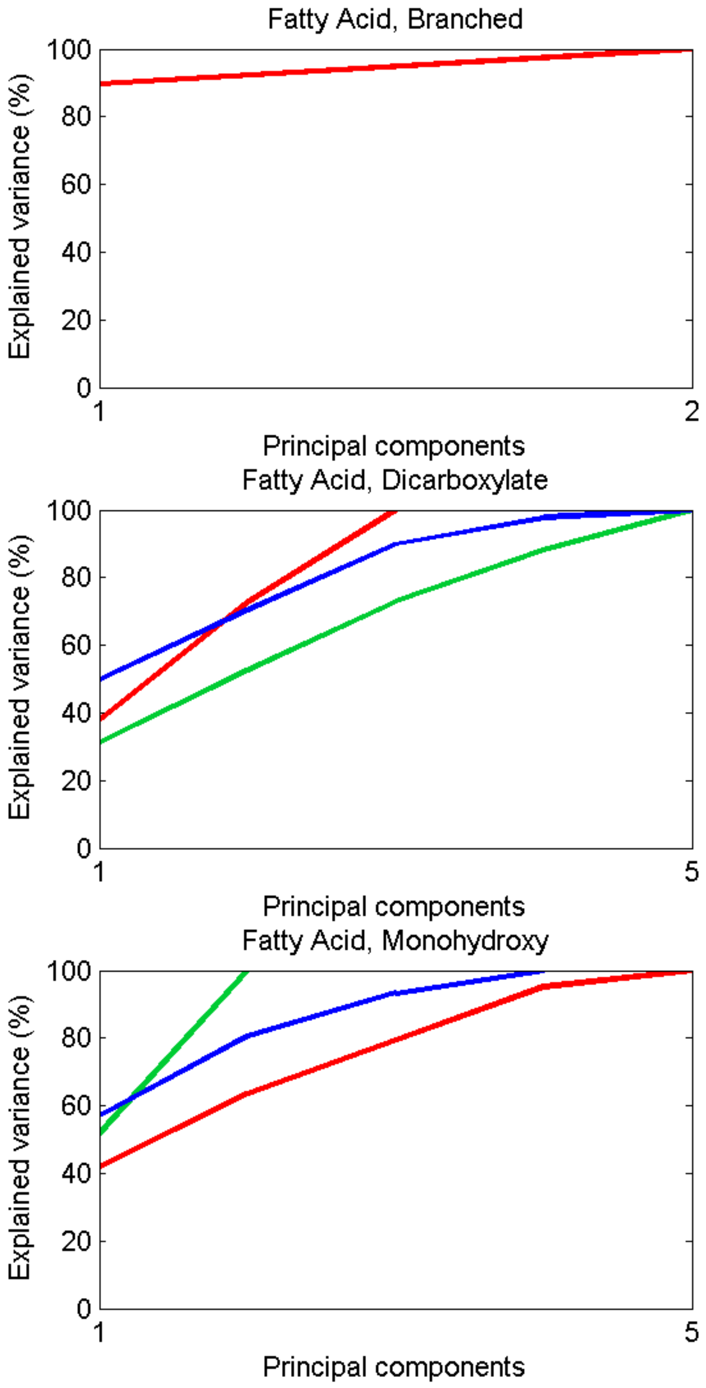

Fatty Acid, Branched
Explained variance (%)
100
80
60
40
20
0
1
2
Principal components
Fatty Acid, Dicarboxylate
Explained variance (%)
100
80
60
40
20
0
1
5
Principal components
Fatty Acid, Monohydroxy
Explained variance (%)
100
80
60
40
20
0
1
5
Principal components

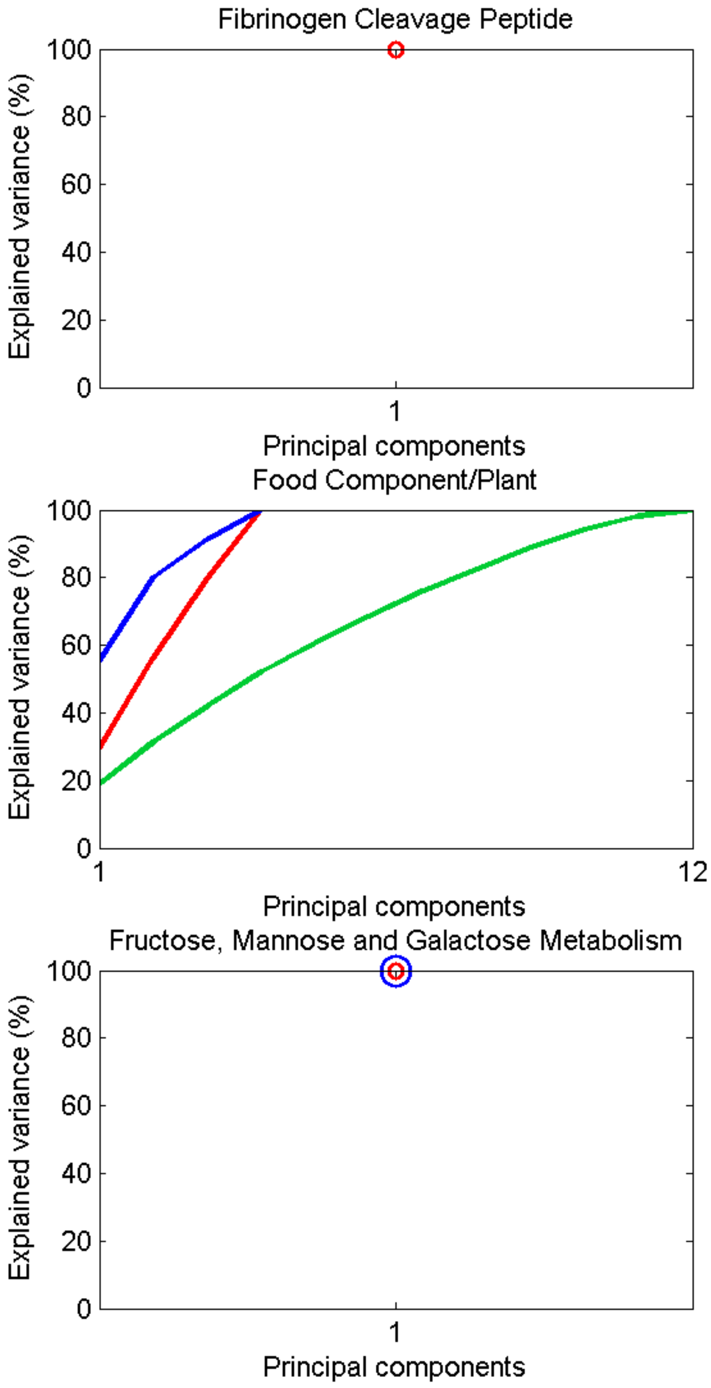

Fibrinogen Cleavage Peptide
Explained variance (%)
100
80
60
40
20
0
1
Principal components
Food Component/Plant
Explained variance (%)
100
80
60
40
20
0
1
12
Principal components
Fructose, Mannose and Galactose Metabolism
Explained variance (%)
100
80
60
40
20
0
1
Principal components

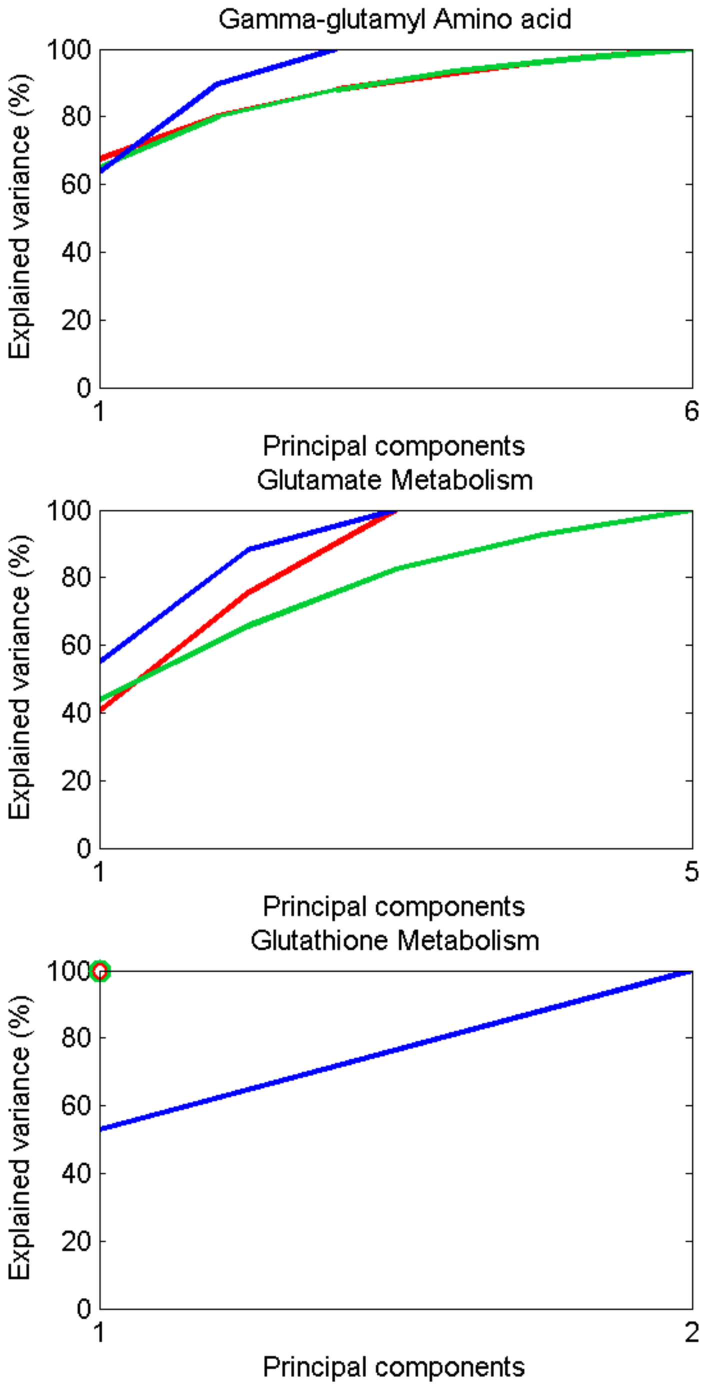

Gamma-glutamyl Amino acid
Explained variance (%)
0
20
40
60
80
100
1
6
Principal components
Glutamate Metabolism
Explained variance (%)
0
20
40
60
80
100
1
5
Principal components
Glutathione Metabolism
Explained variance (%)
0
20
40
60
80
100
1
2
Principal components

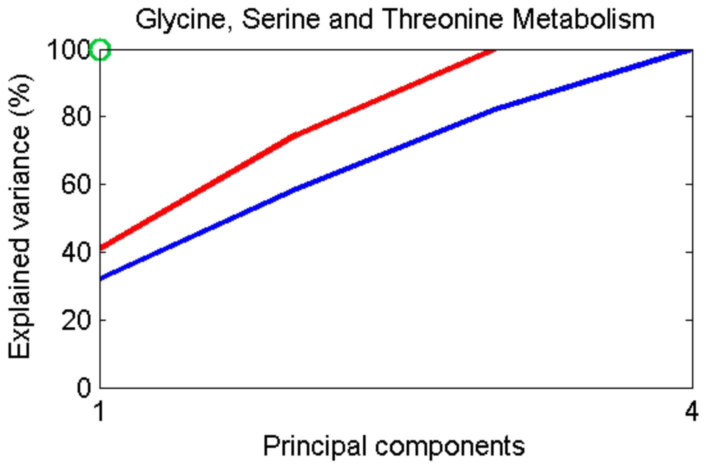
Glycine, Serine and Threonine Metabolism
Explained variance (%)
100
80
60
40
20
0
1
4
Principal components

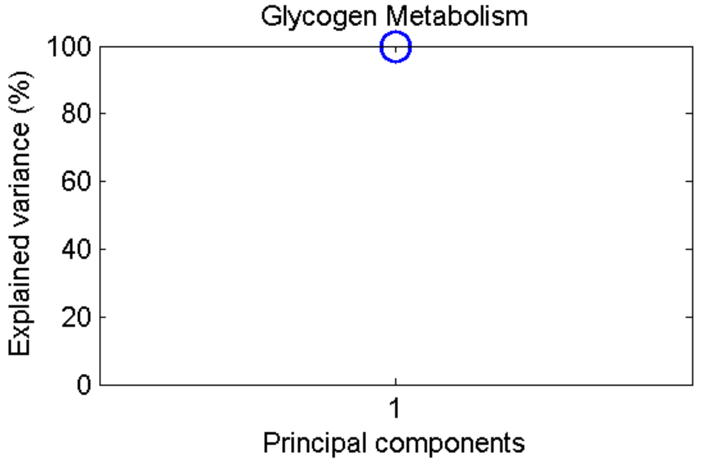
Glycogen Metabolism
Explained variance (%)
100
80
60
40
20
0
1
Principal components

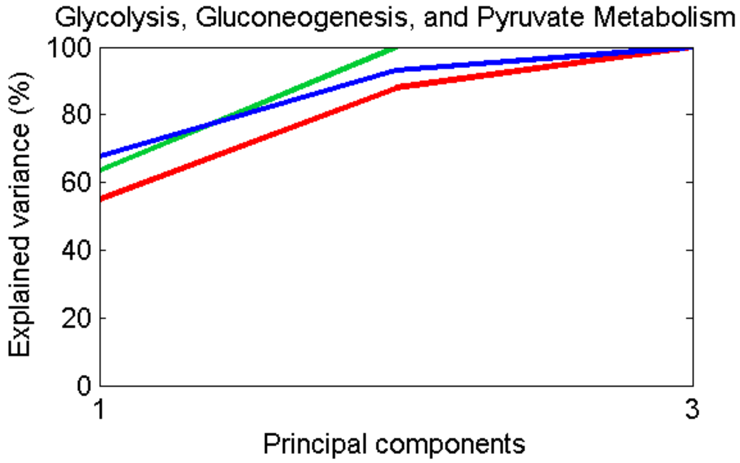
Glycolysis, Gluconeogenesis, and Pyruvate Metabolism
Explained variance (%)
100
80
60
40
20
0
1
3
Principal components

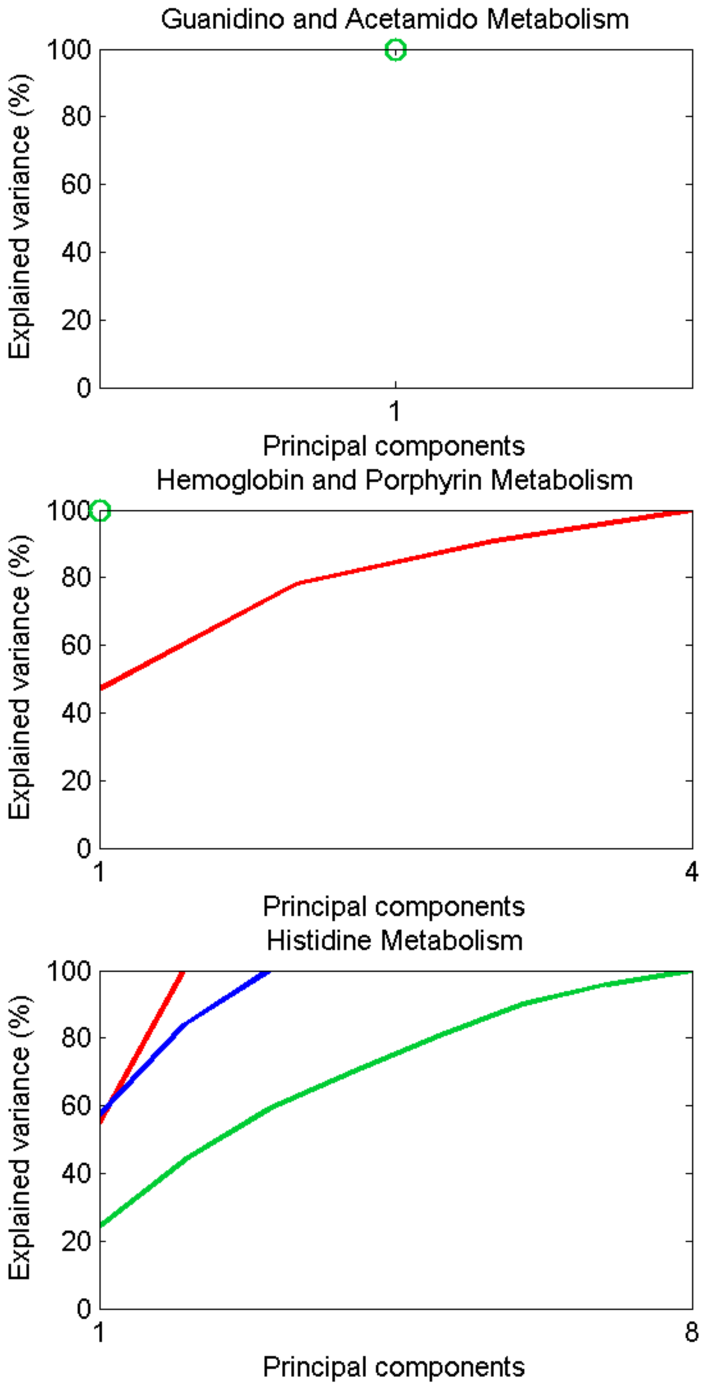
Guanidino and Acetamido Metabolism
100
80
60
40
20
0
Explained variance (%)
1
Principal components
Hemoglobin and Porphyrin Metabolism
100
80
60
40
20
0
Explained variance (%)
1
4
Principal components
Histidine Metabolism
100
80
60
40
20
0
Explained variance (%)
1
8
Principal components

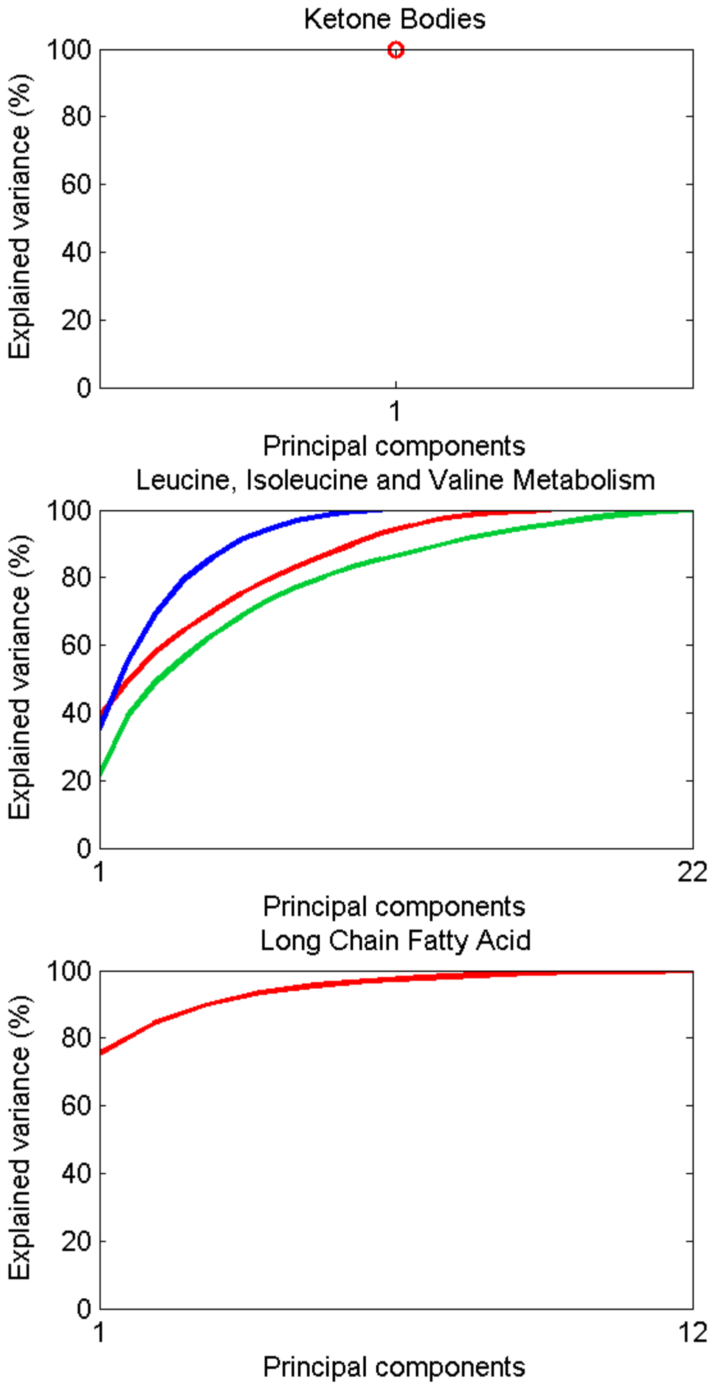

Ketone Bodies
Explained variance (%)
Principal components
Leucine, Isoleucine and Valine Metabolism
Explained variance (%)
Principal components
Long Chain Fatty Acid
Explained variance (%)
Principal components

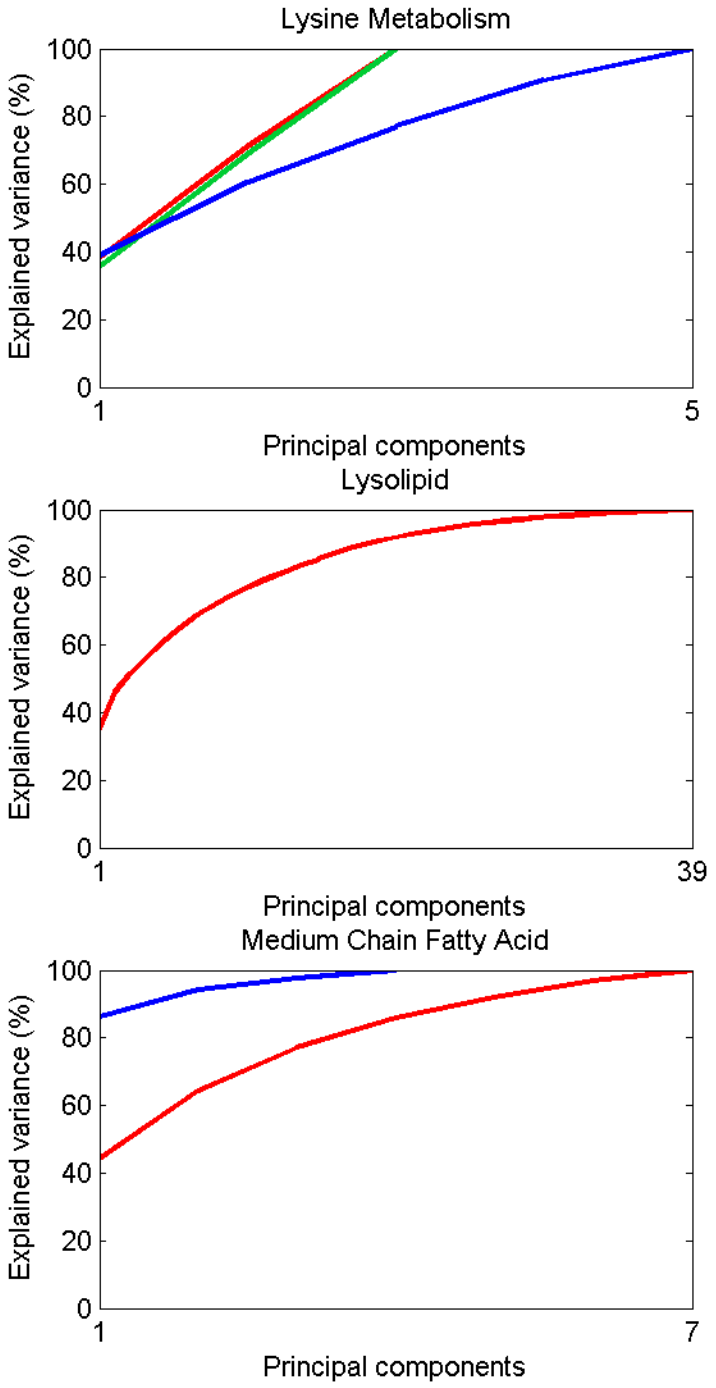
Lysine Metabolism
Explained variance (%)
100
80
60
40
20
0
1
5
Principal components
Lysolipid
Explained variance (%)
100
80
60
40
20
0
1
39
Principal components
Medium Chain Fatty Acid
Explained variance (%)
100
80
60
40
20
0
1
7
Principal components

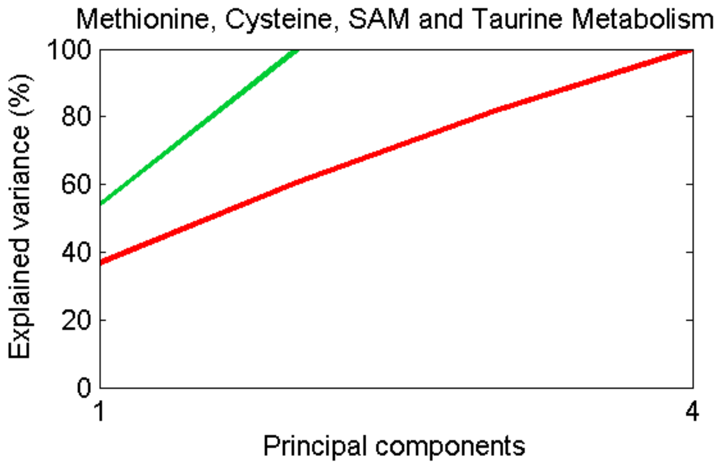
Methionine, Cysteine, SAM and Taurine Metabolism
100
80
60
40
20
0
Explained variance (%)
1
4
Principal components

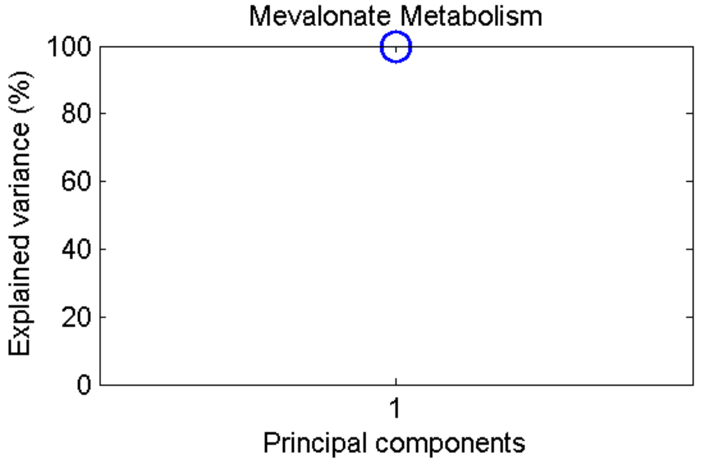
Mevalonate Metabolism
100
80
60
40
20
0
Explained variance (%)
1
Principal components

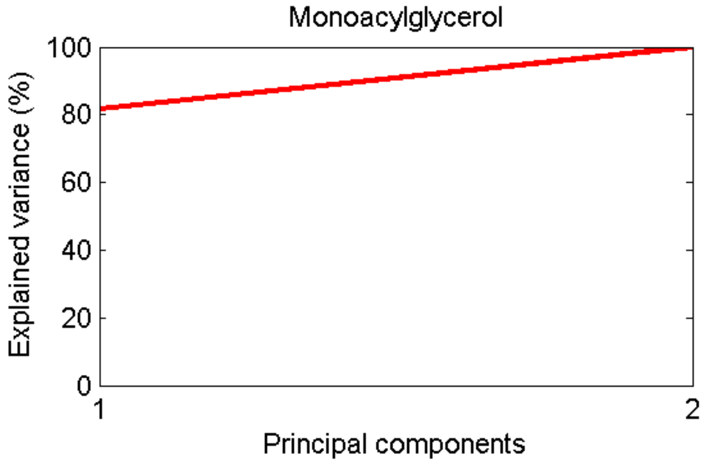
Monoacylglycerol
100
80
60
40
20
0
Explained variance (%)
1
2
Principal components

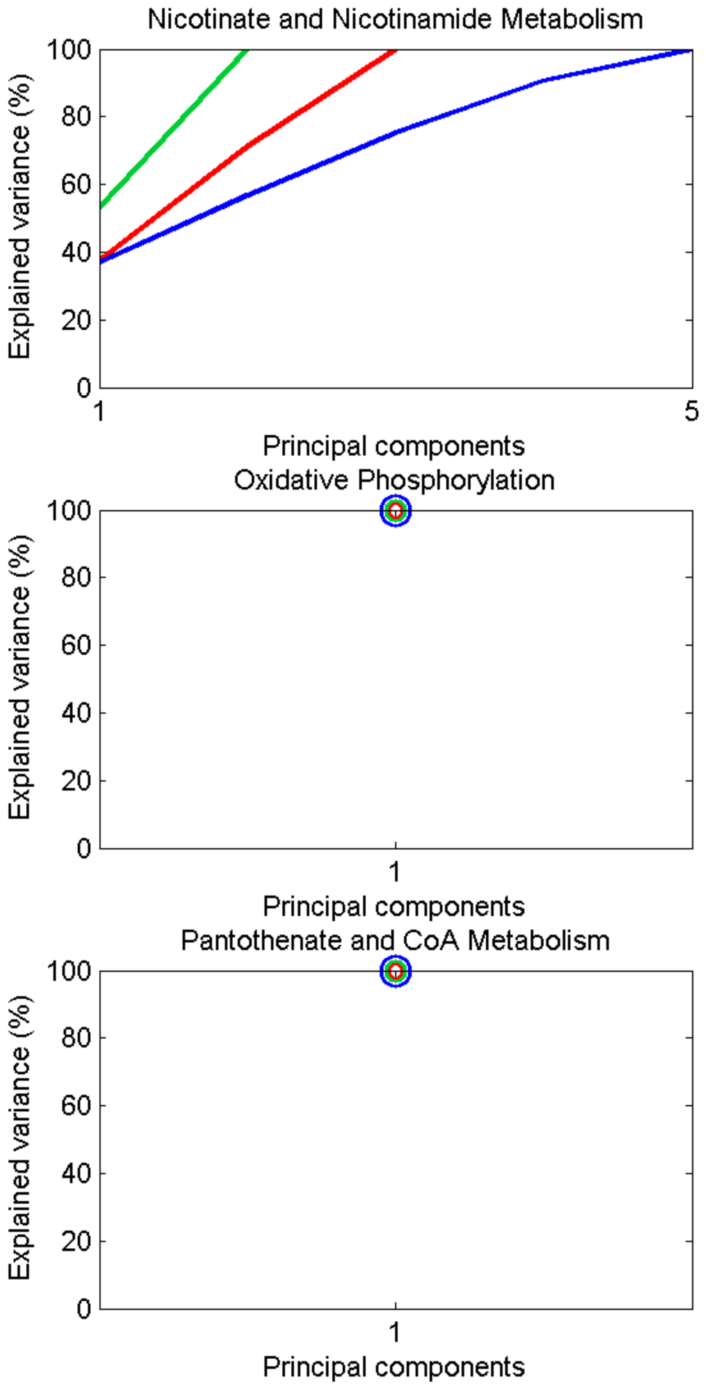
Nicotinate and Nicotinamide Metabolism
Explained variance (%)
100
80
60
40
20
0
1
5
Principal components
Oxidative Phosphorylation
Explained variance (%)
100
80
60
40
20
0
1
Principal components
Pantothenate and CoA Metabolism
Explained variance (%)
100
80
60
40
20
0
1
Principal components

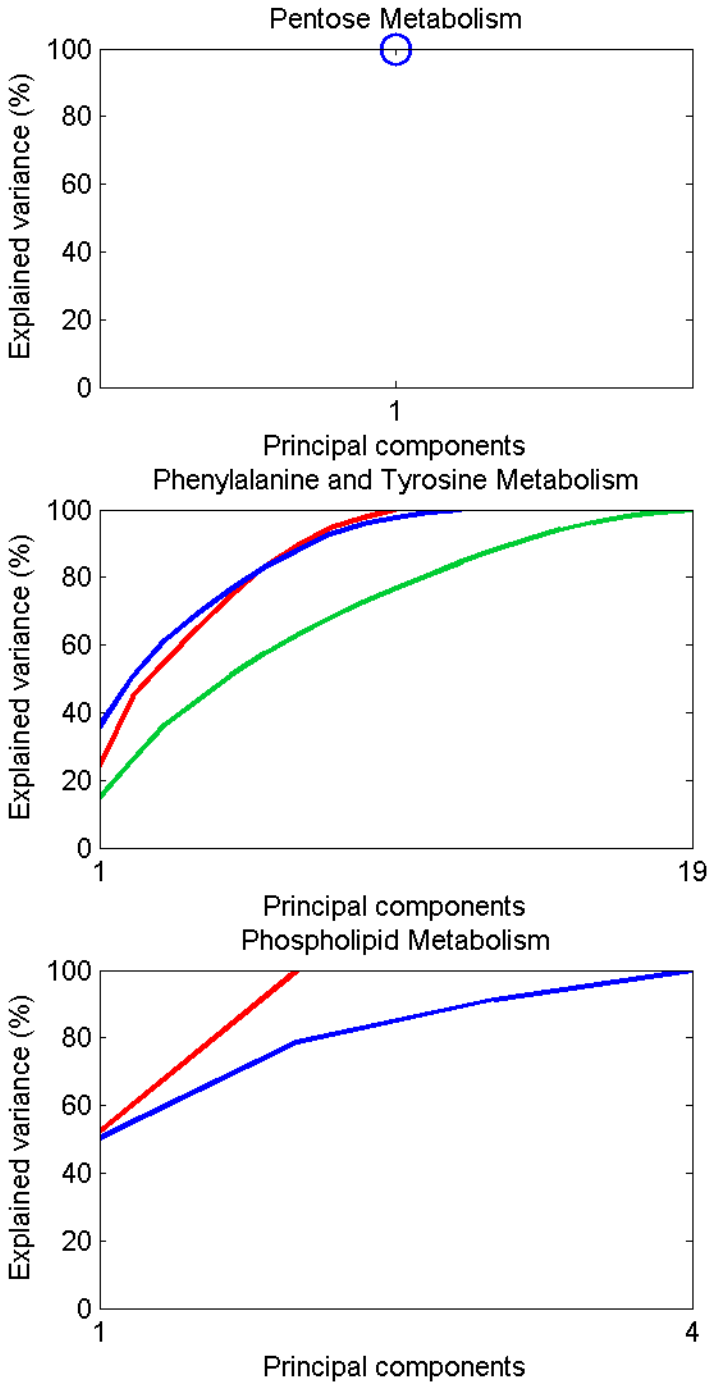
Pentose Metabolism
Explained variance (%)
100
80
60
40
20
0
1
Principal components
Phenylalanine and Tyrosine Metabolism
Explained variance (%)
100
80
60
40
20
0
1
19
Principal components
Phospholipid Metabolism
Explained variance (%)
100
80
60
40
20
0
1
4
Principal components

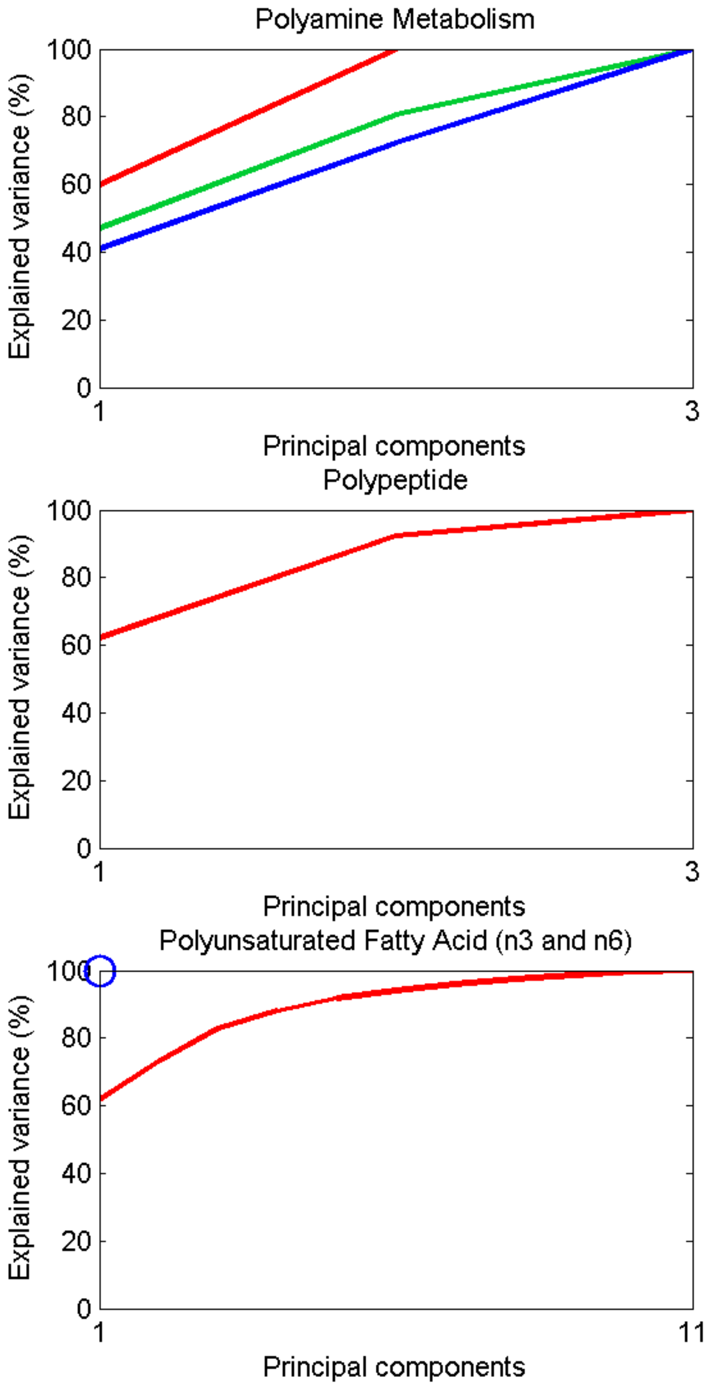
Polyamine Metabolism
Explained variance (%)
100
80
60
40
20
0
1
3
Principal components
Polypeptide
Explained variance (%)
100
80
60
40
20
0
1
3
Principal components
Polyunsaturated Fatty Acid (n3 and n6)
Explained variance (%)
100
80
60
40
20
0
1
11
Principal components

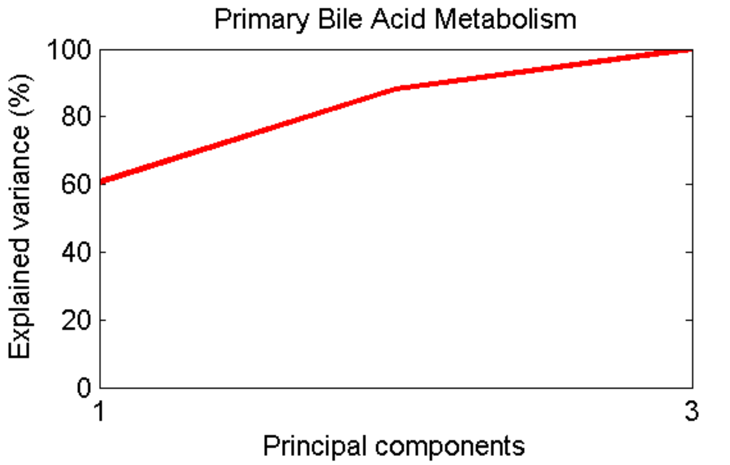
Primary Bile Acid Metabolism
Explained variance (%)
100
80
60
40
20
0
1
3
Principal components

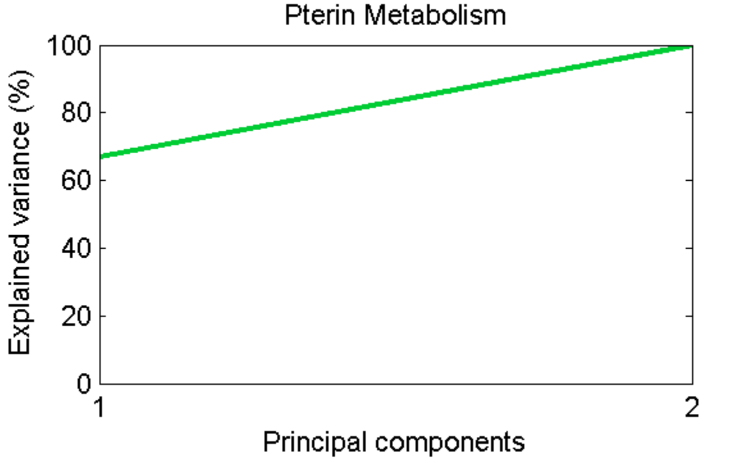
Pterin Metabolism
Explained variance (%)
100
80
60
40
20
0
1
2
Principal components

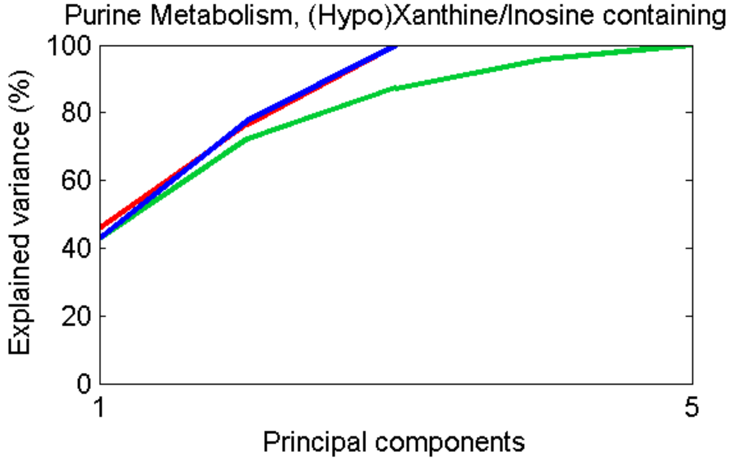
Purine Metabolism, (Hypo)Xanthine/Inosine containing
Explained variance (%)
100
80
60
40
20
0
1
5
Principal components

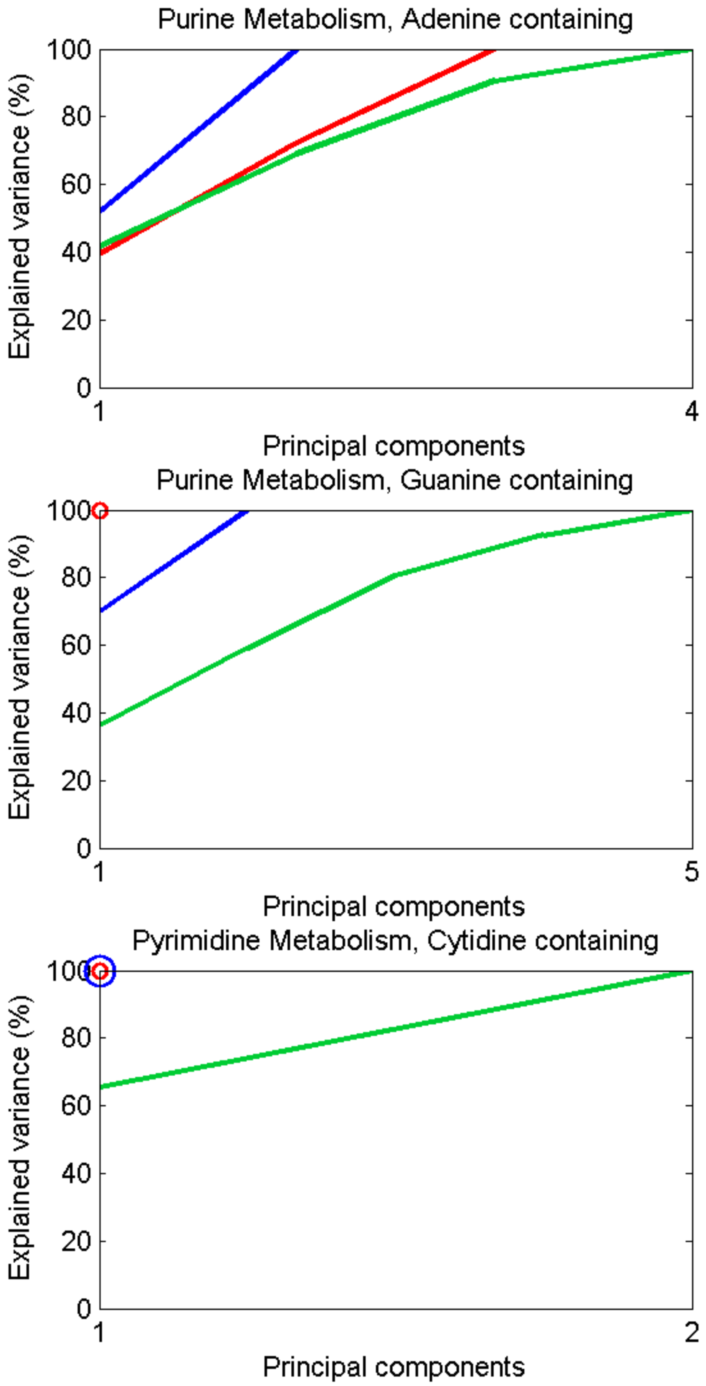
Purine Metabolism, Adenine containing
Explained variance (%)
100
80
60
40
20
0
1
4
Principal components
Purine Metabolism, Guanine containing
Explained variance (%)
100
80
60
40
20
0
1
5
Principal components
Pyrimidine Metabolism, Cytidine containing
Explained variance (%)
100
80
60
40
20
0
1
2
Principal components

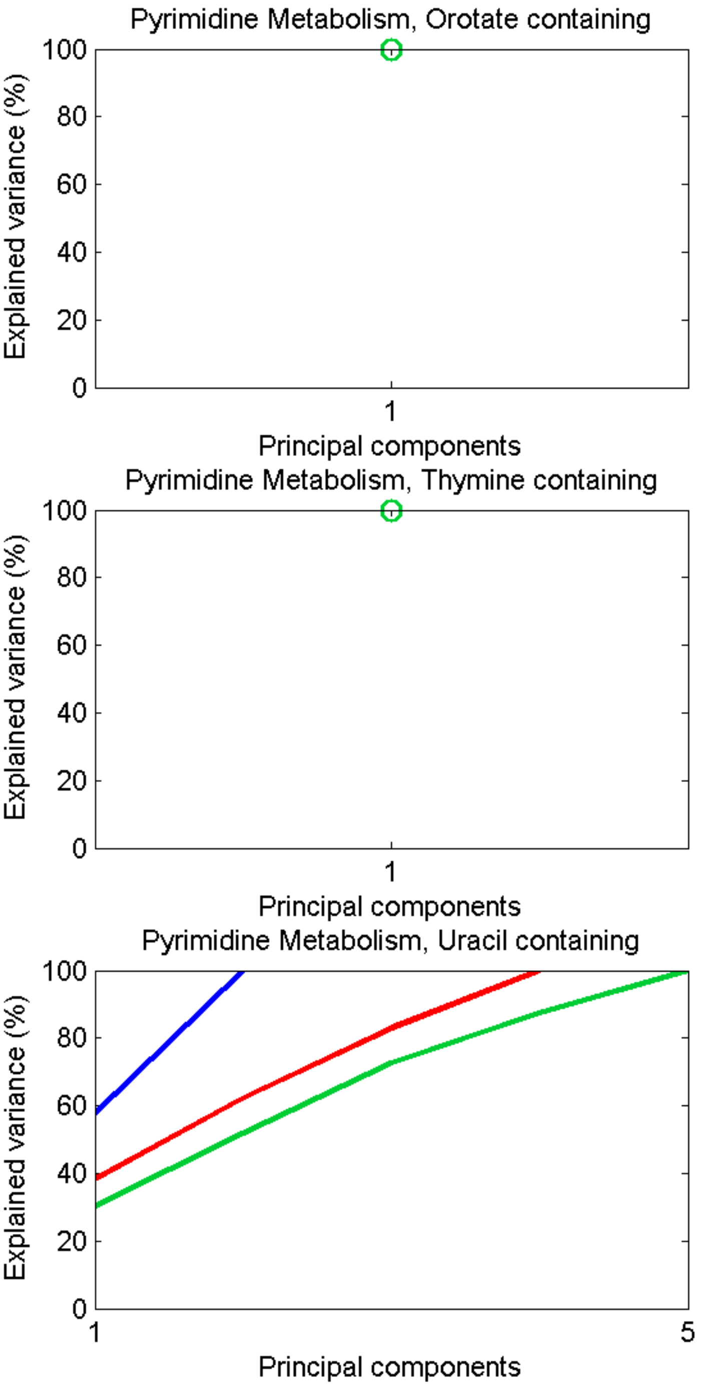
Pyrimidine Metabolism, Orotate containing
Explained variance (%)
100
80
60
40
20
0
1
Principal components
Pyrimidine Metabolism, Thymine containing
Explained variance (%)
100
80
60
40
20
0
1
Principal components
Pyrimidine Metabolism, Uracil containing
Explained variance (%)
100
80
60
40
20
0
1
5
Principal components

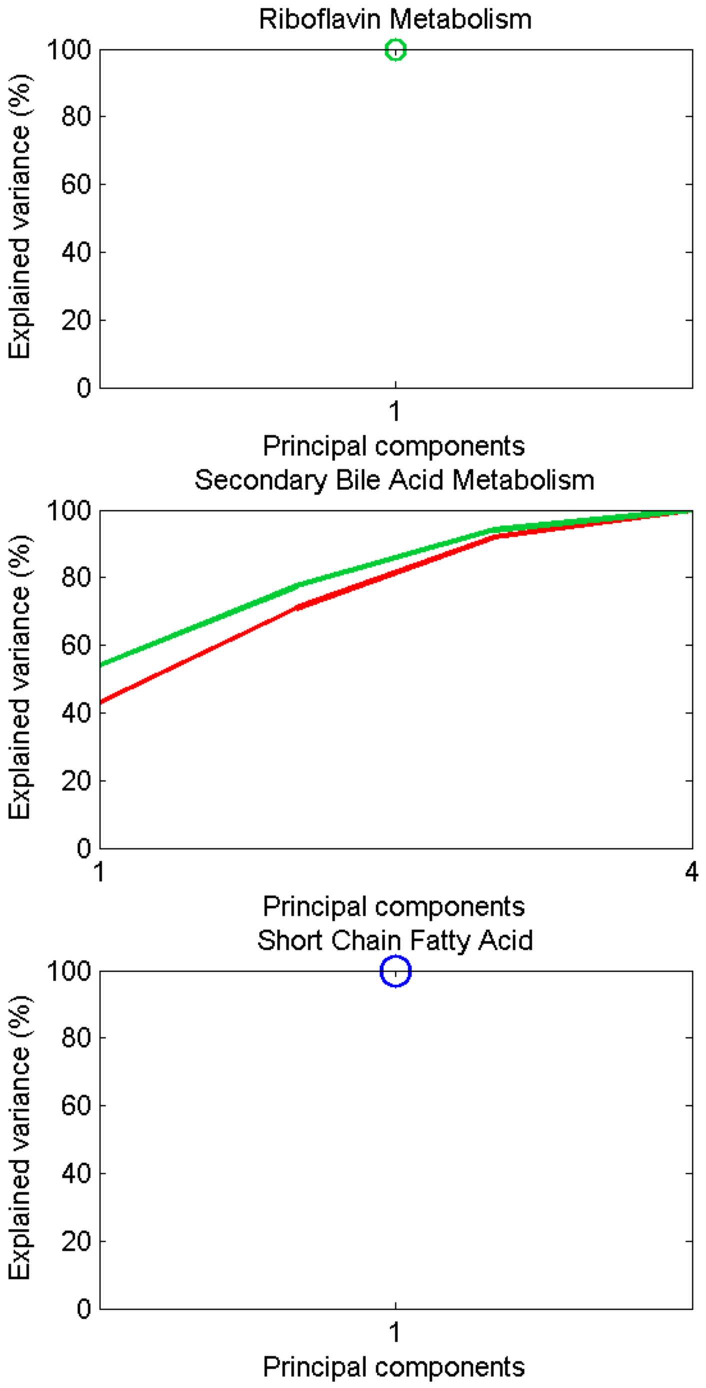

Riboflavin Metabolism
100
80
60
40
20
0
Explained variance (%)
1
Principal components
Secondary Bile Acid Metabolism
100
80
60
40
20
0
Explained variance (%)
1
4
Principal components
Short Chain Fatty Acid
100
80
60
40
20
0
Explained variance (%)
1
Principal components

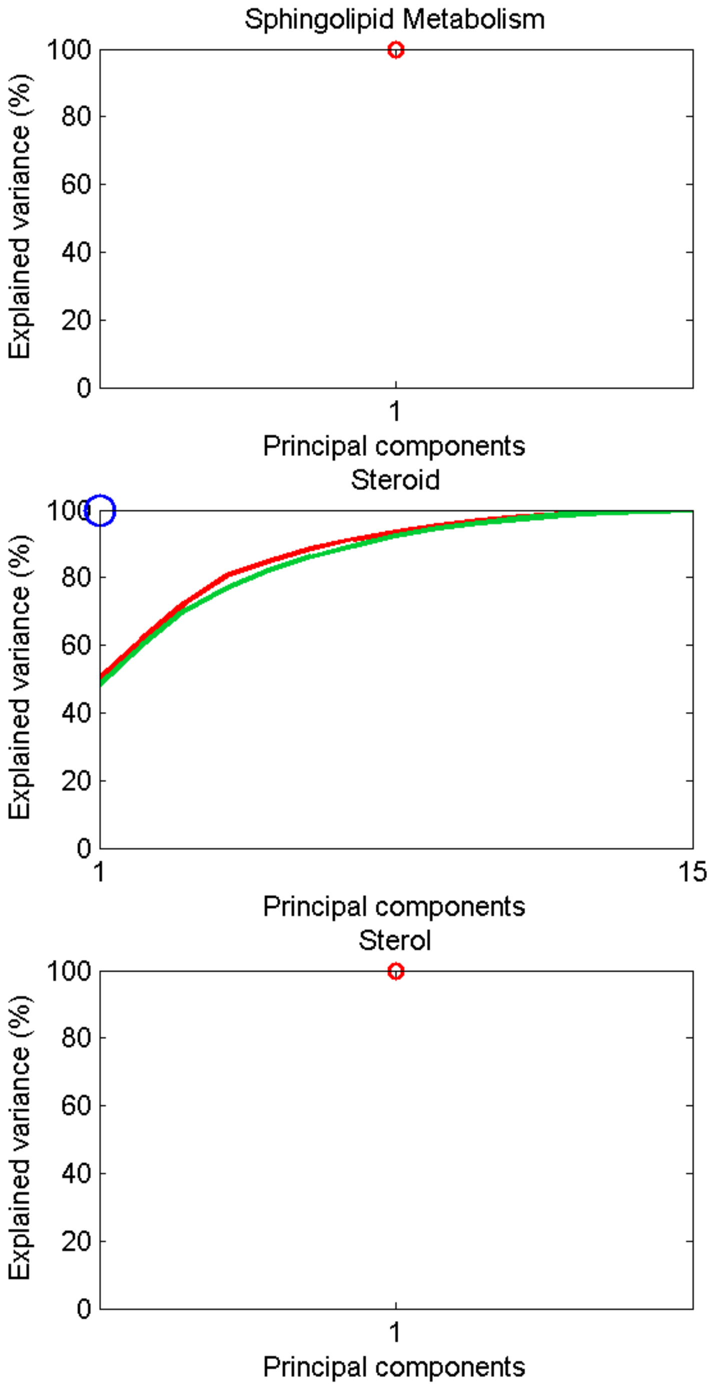
Sphingolipid Metabolism
Explained variance (%)
100
80
60
40
20
0
1
Principal components
Steroid
Explained variance (%)
100
80
60
40
20
0
1
15
Principal components
Sterol
Explained variance (%)
100
80
60
40
20
0
1
Principal components

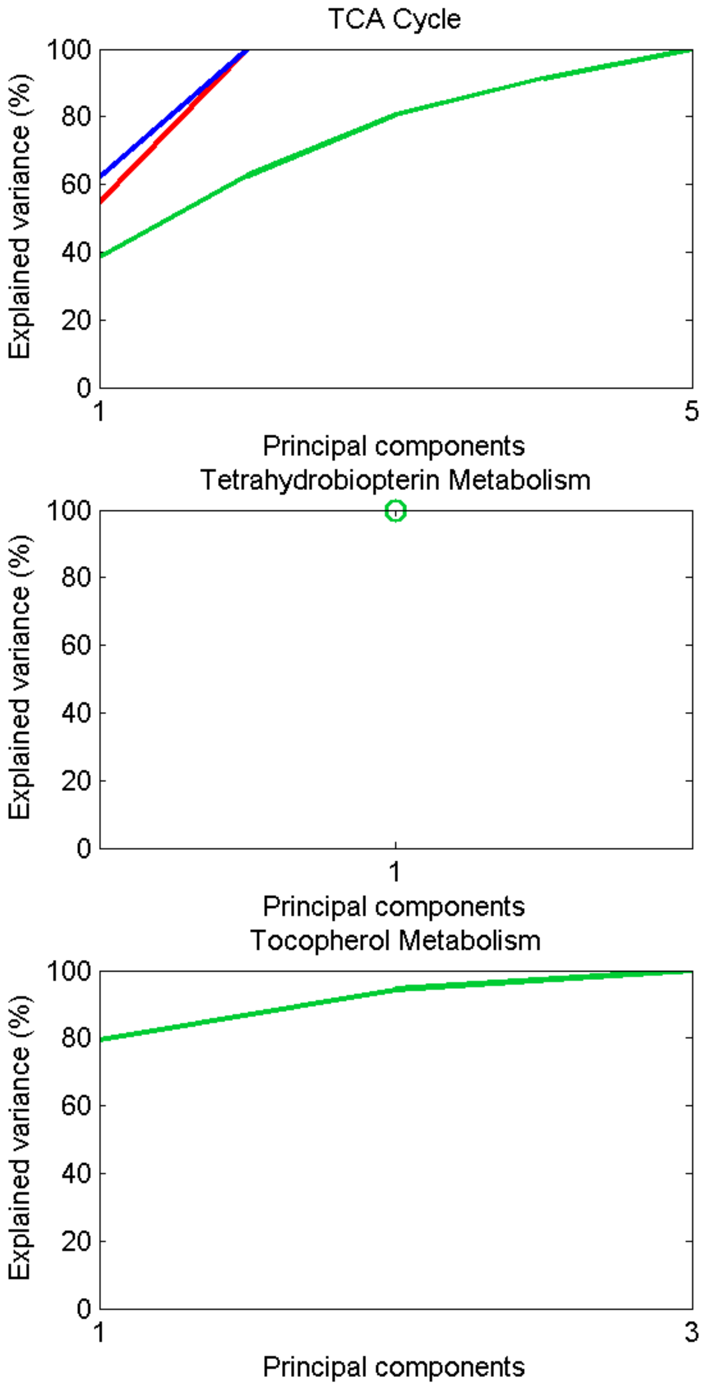
TCA Cycle
Explained variance (%)
100
80
60
40
20
0
1
5
Principal components
Tetrahydrobiopterin Metabolism
Explained variance (%)
100
80
60
40
20
0
1
Principal components
Tocopherol Metabolism
Explained variance (%)
100
80
60
40
20
0
1
3
Principal components

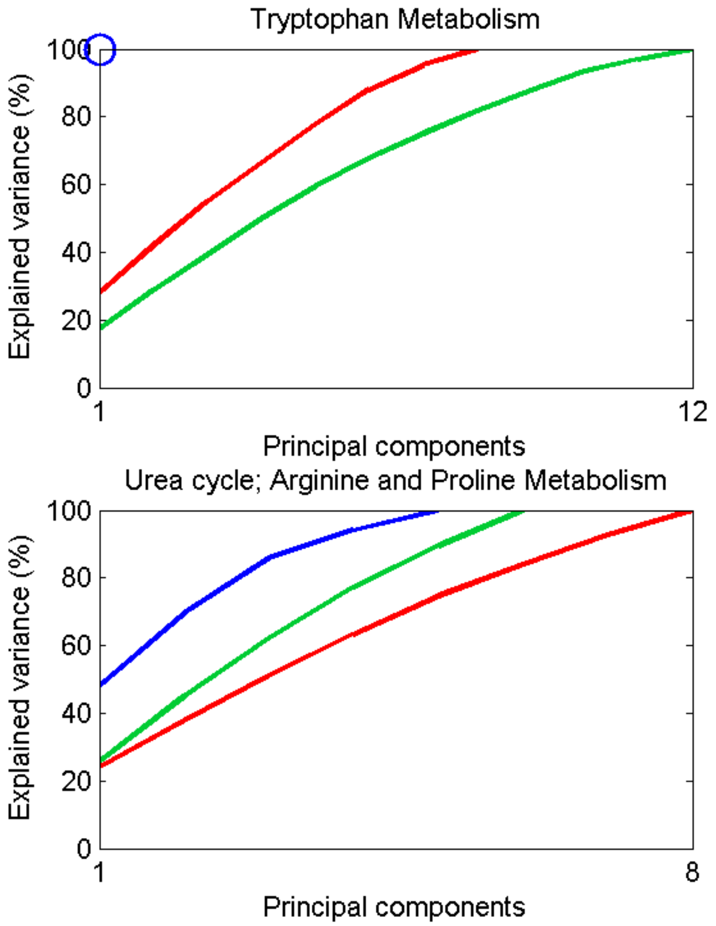

Tryptophan Metabolism
Explained variance (%)
100
80
60
40
20
0
1
12
Principal components
Urea cycle; Arginine and Proline Metabolism
Explained variance (%)
100
80
60
40
20
0
1
8
Principal components

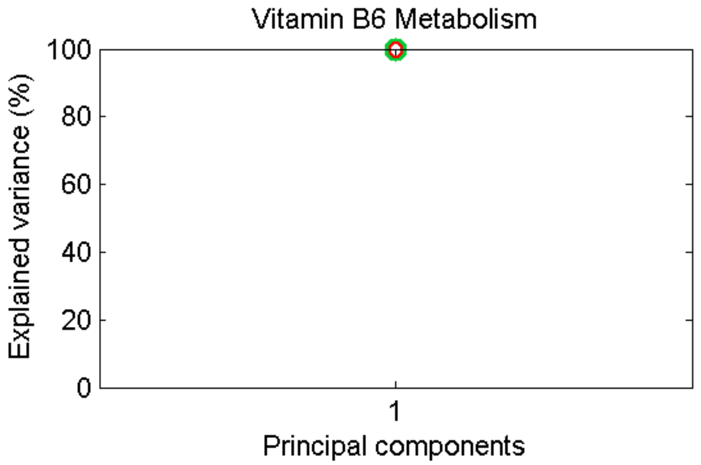
Vitamin B6 Metabolism
Explained variance (%)
100
80
60
40
20
0
1
Principal components

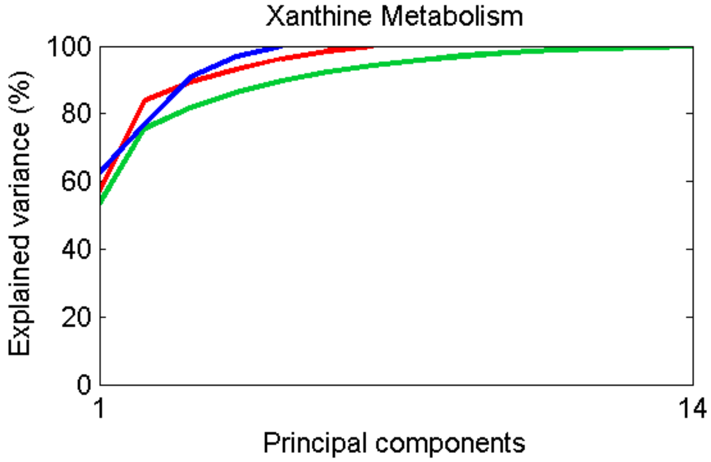
Xanthine Metabolism
Explained variance (%)
100
80
60
40
20
0
1
14
Principal components

Super-pathways

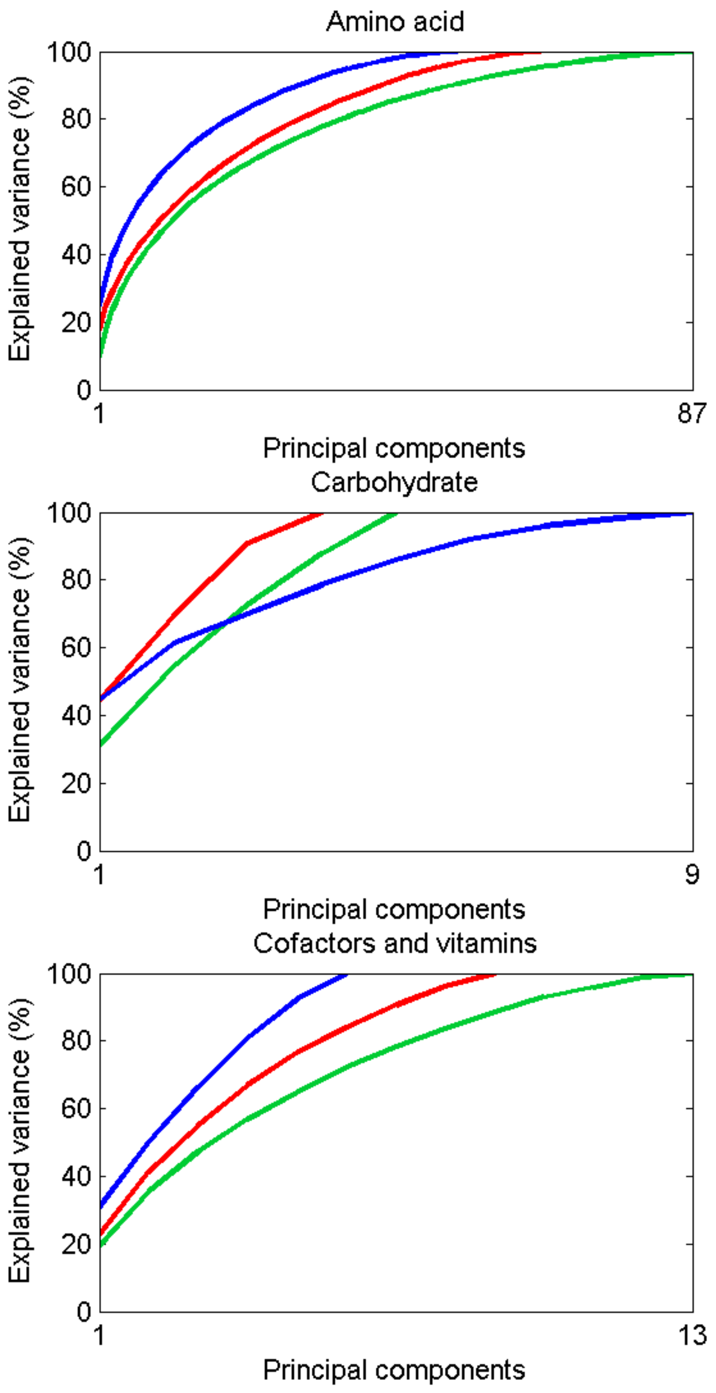

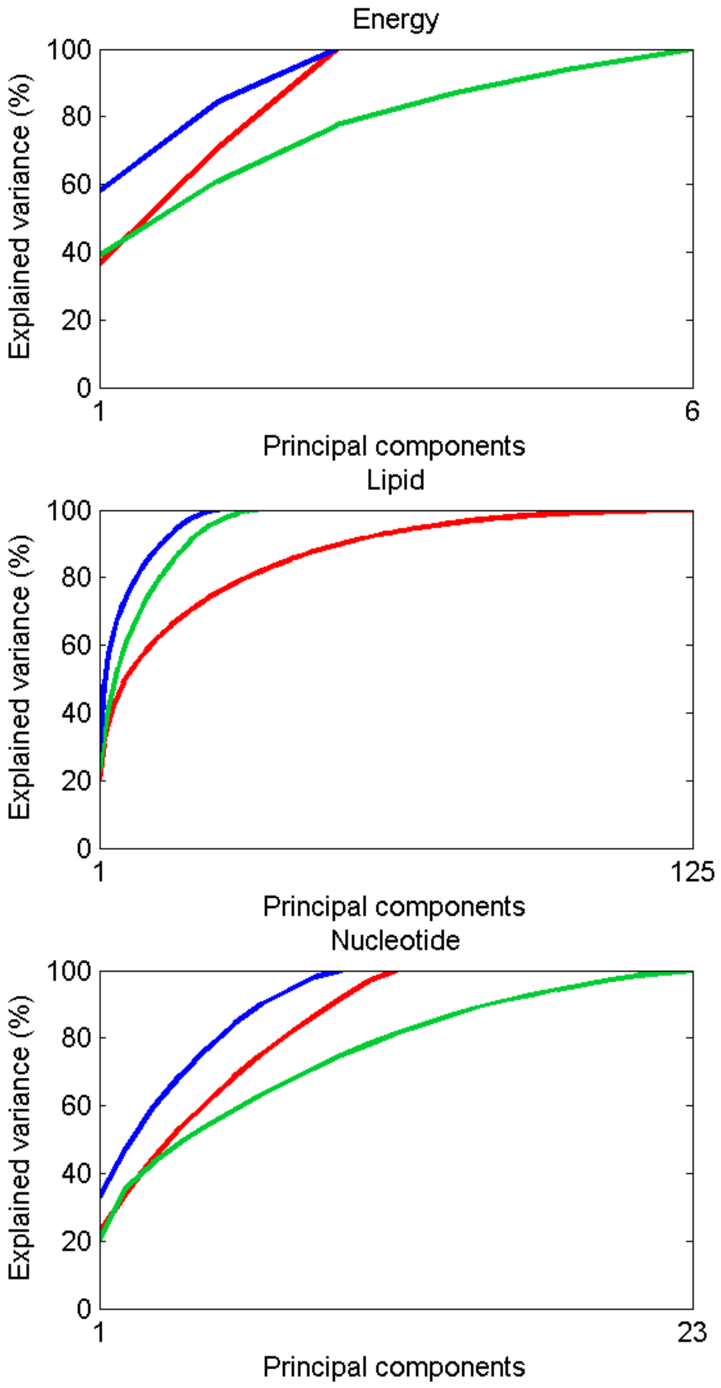

Energy
Explained variance (%)
100
80
60
40
20
0
1
6
Principal components
Lipid
Explained variance (%)
100
80
60
40
20
0
1
125
Principal components
Nucleotide
Explained variance (%)
100
80
60
40
20
0
1
23
Principal components

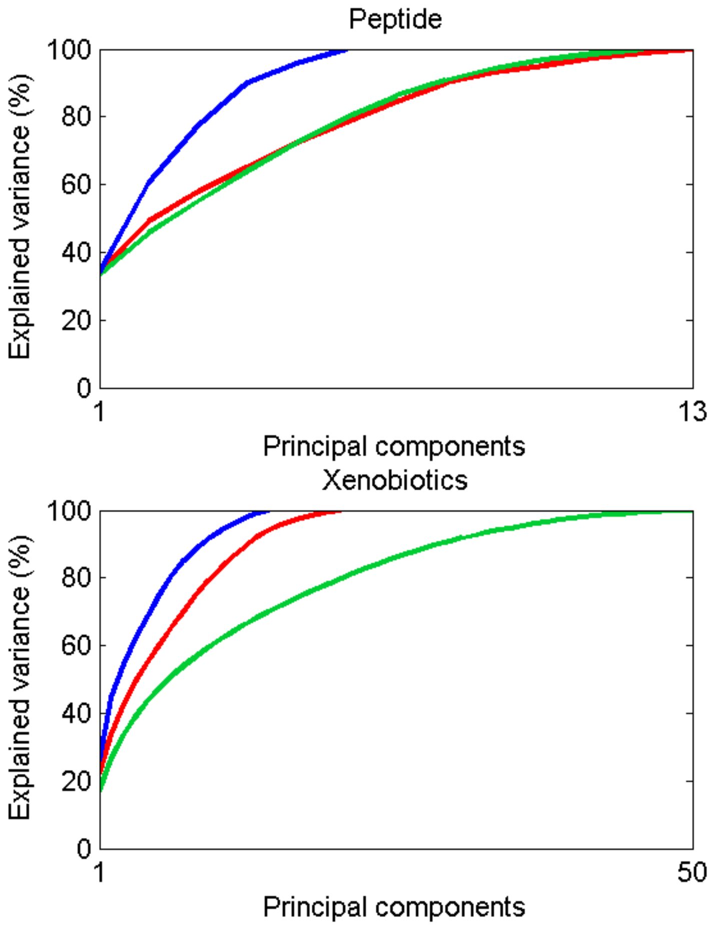
Peptide
Explained variance (%)
100
80
60
40
20
0
1
13
Principal components
Xenobiotics
Explained variance (%)
100
80
60
40
20
0
1
50
Principal components
