# Supplementary material for: Phenotype-driven identification of modules in a hierarchical map of multifluid metabolic correlations
Source: NPJ Syst Biol Appl. 2017 Sep 21;3:28. doi: 10.1038/s41540-017-0029-9 (PMC5608949; doi:10.1038/s41540-017-0029-9)
Supplement: Supplementary file 1 — Supplementary Material [file 41540_2017_29_MOESM1_ESM.zip › Supplement_onlineVersion/SupportingInformation_S5_Effects of oral hygiene.docx]

Phenotype-driven identification of modules in a hierarchical map of multifluid metabolic correlations

*Kieu Trinh Do, Maik Pietzner, David Rasp, Nele Friedrich, Matthias Nauck, Thomas Kocher, Karsten Suhre, Dennis O. Mook-Kanamori, Gabi Kastenmüller, Jan Krumsiek*

**Supporting Information S5: Influence of oral hygiene on the metabolic correlations and modules**

Since salivary measurements are particularly dependent on the oral microbiome, we investigated whether oral hygiene has any effects on the estimated correlation structures and the metabolic changes associated with IGF-I and gender.

In SHIP-TREND the majority of study participants brushed their teeth two times daily. Several people brushed their teeth at least three times and several people once per day. Few cleaned their teeth only several times per week or even (less than) once a week (Figure S5A).


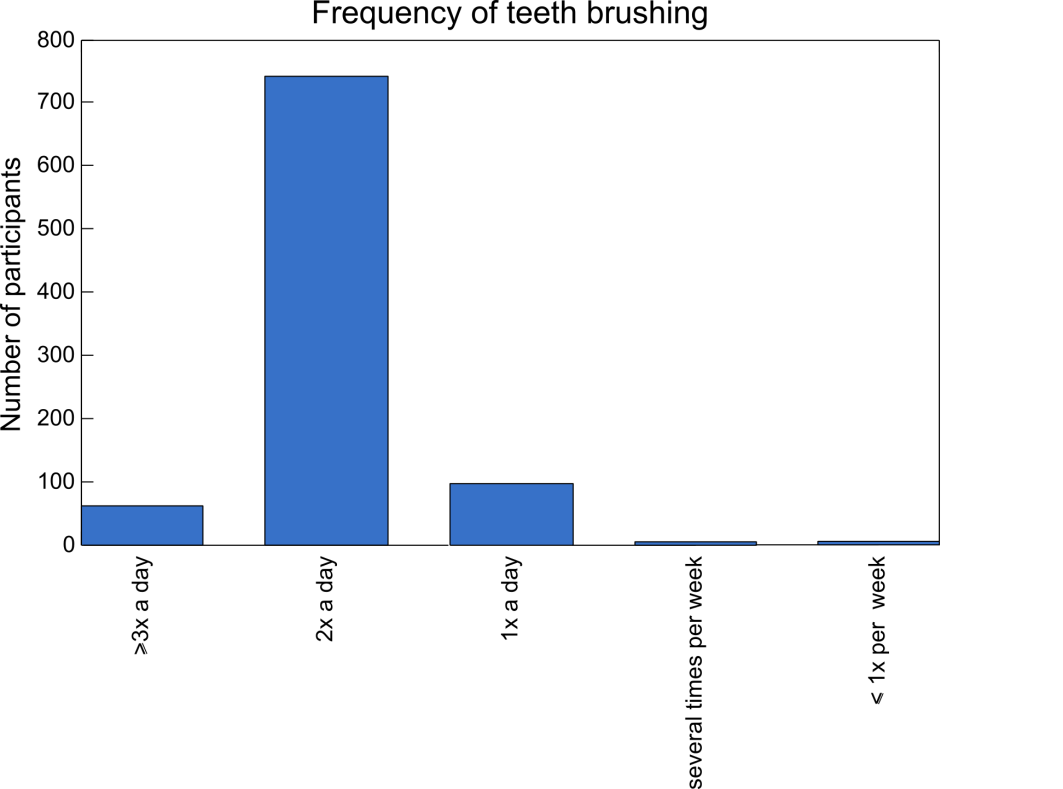


Figure S5A. Frequency of teeth brushing of participants of the SHIP-TREND cohort.

To analyze whether oral hygiene changes the metabolic correlation structures we recalculated the hierarchical map and included the frequency of teeth brushing (Figure S5A) as a covariate, that is, we eliminated its effects from the hierarchical map. Subsequently, we compared the recalculated hierarchical map with the original hierarchical map (Figure S5B). Only marginal differences in partial correlations were observed, most likely due to statistical variance than biologically driven by oral hygiene.


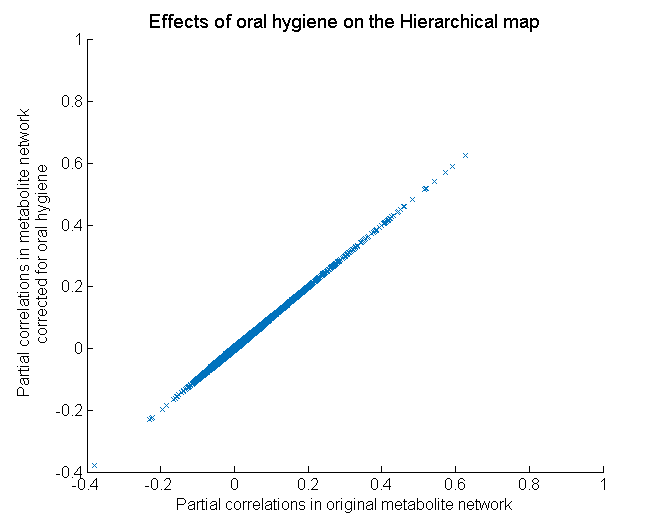

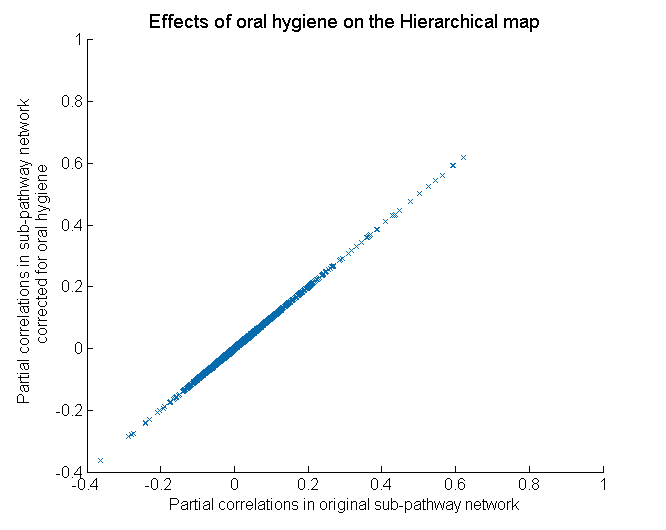


Figure S5B. Effects of oral hygiene on the hierarchical map.

We observed no association of teeth brushing behavior with IGF-I levels (Figure S5C). However, males tend to brush their teeth significantly less than women (Fisher’s exact test p-value with discretized teeth brushing behavior is $6.17e-07$, Table S5A).


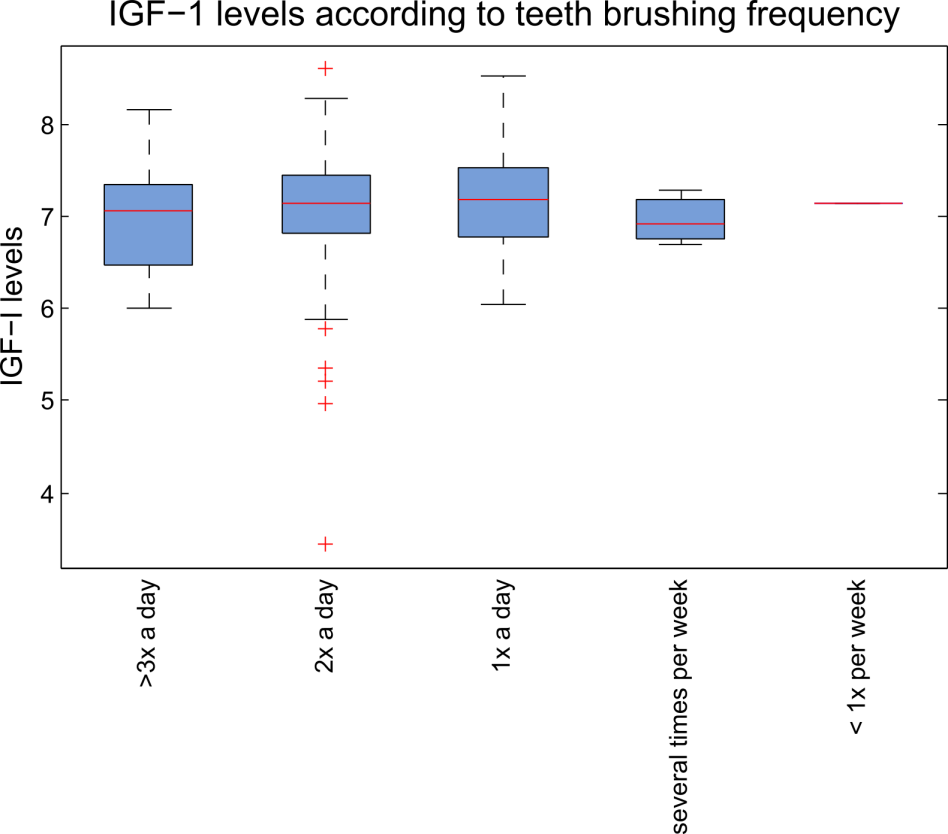


Figure S5C. IGF-I levels according to teeth brushing behavior.

|  | frequent | | not frequent | | |
| --- | --- | --- | --- | --- | --- |
|  | ≥3x a day | 2x a day | 1x a day | several times per week | ≤1x per week |
| male | 1 | 3 | 63 | 306 | 21 |
| female | 0 | 0 | 33 | 437 | 40 |

Table S5A. Frequency of teeth brushing in females and males.

To analyze whether the modules identified for IGF-I and gender were affected by oral hygiene, we performed the module identification approach as described in the main manuscript, but included teeth brushing frequency as a covariate in the module scoring function to eliminate effects of this variable. For IGF-I the same modules were identified (Table S5B). Despite the significant association of teeth brushing frequency with gender (Table S5A), correcting for teeth brushing behavior led to nearly the same modules (Table S5C). Only module E in the main manuscript was not detected.

| **Module_effectSize** | **Module_pval** | **Component** | **Fluid** | **Component_pval** | **Component_effectSize** |
| --- | --- | --- | --- | --- | --- |
| -0.3442699 | 2.29E-05 | '2-ethylhexanoate (isobar with 2-propylpentanoate)' | P | 2.29E-05 | -0.3442699 |
| -0.278967989 | 9.56E-06 | 'caprylate (8:0)' | P | 0.008359474 | -0.213666078 |
| 0.353274864 | 9.88E-06 | '5-methyluridine (ribothymidine)' | P | 9.88E-06 | 0.353274864 |
| 0.335199678 | 1.76E-07 | 'uridine' | P | 8.74E-05 | 0.317124491 |
| 0.31020266 | 1.31E-08 | 'uracil' | U | 0.001200499 | 0.260208624 |
| 0.296912254 | 9.19E-05 | 'androsterone sulfate' | P | 9.19E-05 | 0.296912254 |
| 0.279806271 | 2.18E-05 | 'androsterone sulfate' | U | 0.000290504 | 0.262700289 |
| 0.284081894 | 1.49E-05 | 'epiandrosterone sulfate' | P | 8.77E-05 | 0.29263314 |
| 0.239312496 | 0.001576095 | 'X - 12846' | P | 0.001576095 | 0.239312496 |
| 0.236509173 | 0.000301703 | 'X - 12846' | U | 0.002095962 | 0.233705851 |
| 0.283670277 | 1.20E-06 | '11-ketoetiocholanolone glucuronide' | U | 2.58E-06 | 0.377992486 |
| 0.282547276 | 2.84E-07 | 'X - 11444' | U | 0.000249414 | 0.279178272 |
| 0.299308199 | 4.43E-08 | 'X - 12844' | U | 2.93E-06 | 0.366351892 |
| 0.325949596 | 1.88E-09 | 'X - 12844' | P | 3.66E-09 | 0.459156582 |
| 0.319567537 | 1.62E-09 | 'X - 17357' | P | 0.000198844 | 0.281275178 |
| 0.280294933 | 0.000107402 | 'X - 17340' | P | 0.000107402 | 0.280294933 |
| 0.279412634 | 1.31E-05 | 'tetrahydrocortisone' | U | 0.000266674 | 0.278530334 |
| 0.243264855 | 3.66E-06 | 'X - 11945' | P | 0.032346646 | 0.170969296 |
| 0.244552068 | 1.30E-06 | 'X - 17359' | P | 0.000684906 | 0.24841371 |
| 0.242027117 | 7.59E-07 | 'X - 17341' | U | 0.002886541 | 0.231927313 |
| 0.191112113 | 0.012483359 | 'X - 17339' | U | 0.012483359 | 0.191112113 |
| 0.219762911 | 0.000408082 | 'X - 17359' | P | 0.000684906 | 0.24841371 |
| 0.239940252 | 2.26E-05 | 'X - 17340' | P | 0.000107402 | 0.280294933 |
| 0.222697513 | 5.99E-06 | 'X - 11945' | P | 0.032346646 | 0.170969296 |
| 0.233864077 | 1.59E-06 | 'tetrahydrocortisone' | U | 0.000266674 | 0.278530334 |
| 0.233541283 | 1.34E-06 | 'X - 17341' | U | 0.002886541 | 0.231927313 |
| 0.257704376 | 0.000565199 | 'X - 17340' | U | 0.000565199 | 0.257704376 |
| 0.268999654 | 3.12E-05 | 'X - 17340' | P | 0.000107402 | 0.280294933 |
| 0.262137673 | 9.13E-06 | 'X - 17359' | P | 0.000684906 | 0.24841371 |
| 0.239345579 | 2.98E-06 | 'X - 11945' | P | 0.032346646 | 0.170969296 |
| 0.24718253 | 1.34E-06 | 'tetrahydrocortisone' | U | 0.000266674 | 0.278530334 |
| 0.244639994 | 1.25E-06 | 'X - 17341' | U | 0.002886541 | 0.231927313 |
| 0.232630413 | 0.004372327 | '2-hydroxyglutarate' | S | 0.004372327 | 0.232630413 |
| 0.245495777 | 0.000536132 | 'phenyllactate (PLA)' | S | 0.001446483 | 0.258361141 |
| 0.241770588 | 0.000531965 | '3-(4-hydroxyphenyl)lactate' | S | 0.003620613 | 0.23432021 |
| 0.252358717 | 0.000114994 | '5-aminovalerate' | S | 0.000431217 | 0.284123104 |
| 0.201753554 | 4.33E-05 | 'lauryl sulfate' | S | 0.993476869 | -0.0006671 |

Table S5B. IGF-I related modules corrected for oral hygiene.

| **Module_effectSize** | **Module_pval** | **Component** | **Fluid** | **Component_pval** | **Component_effectSize** |
| --- | --- | --- | --- | --- | --- |
| -0.223675459 | 1.19E-09 | 'P::Chemical' | P | 1.19E-09 | -0.223675459 |
| -0.237472238 | 3.97E-13 | 'P::Pyrimidine Metabolism, Uracil containing' | P | 5.99E-10 | -0.251269016 |
| -0.255877098 | 2.09E-05 | 'P::Monoacylglycerol' | P | 2.09E-05 | -0.255877098 |
| -0.22681592 | 1.35E-09 | 'P::Lysolipid' | P | 3.06E-09 | -0.225325603 |
| -0.238203974 | 7.24E-12 | 'P::Fatty Acid Metabolism(Acyl Carnitine)' | P | 6.58E-09 | -0.284894998 |
| 0.448061666 | 1.01E-15 | 'P::Long Chain Fatty Acid' | P | 1.01E-15 | 0.448061666 |
| 0.381207703 | 4.03E-16 | 'P::Medium Chain Fatty Acid' | P | 7.81E-10 | 0.266600909 |
| 0.386965327 | 2.96E-16 | 'P::Fatty Acid, Branched' | P | 1.59E-12 | 0.441662756 |
| -0.339689405 | 6.29E-10 | 'P::Fatty Acid, Amino' | P | 6.29E-10 | -0.339689405 |
| -0.33239032 | 3.21E-12 | 'U::Fatty Acid, Amino' | U | 2.38E-06 | -0.31779215 |
| -0.515999567 | 8.48E-39 | 'P::Steroid' | P | 8.48E-39 | -0.515999567 |
| -0.45993183 | 2.74E-43 | 'U::Steroid' | U | 4.44E-30 | -0.403864093 |
| -0.527459293 | 6.70E-23 | 'P::Fatty Acid Metabolism (also BCAA Metabolism)' | P | 6.70E-23 | -0.527459293 |
| -0.583405376 | 1.70E-61 | 'P::Leucine, Isoleucine and Valine Metabolism' | P | 3.52E-60 | -0.589987269 |
| -0.634027288 | 6.66E-73 | 'P::Gamma-glutamyl Amino acid' | P | 1.74E-62 | -0.794330007 |
| -0.630161284 | 2.75E-84 | 'P::Glutamate Metabolism' | P | 1.92E-52 | -0.597944584 |
| -1.138098496 | 3.97E-77 | 'P::Dipeptide Derivative' | P | 3.97E-77 | -1.138098496 |
| -0.785770344 | 3.67E-78 | 'U::Dipeptide Derivative' | U | 5.79E-44 | -0.609606268 |
| 0.251243619 | 2.32E-07 | 'U::Xanthine Metabolism' | U | 2.32E-07 | 0.251243619 |
| 0.245236721 | 6.01E-08 | 'U::Nicotinate and Nicotinamide Metabolism' | U | 9.53E-06 | 0.203188434 |
| 0.245065577 | 2.14E-12 | 'U::Pyrimidine Metabolism, Uracil containing' | U | 2.14E-12 | 0.245065577 |
| 0.236224734 | 2.22E-13 | 'U::Purine Metabolism, (Hypo)Xanthine/Inosine containing' | U | 1.40E-07 | 0.227383892 |
| 0.506796207 | 8.62E-36 | 'U::Glutamate Metabolism' | U | 8.62E-36 | 0.506796207 |
| 0.539720289 | 2.16E-40 | 'U::Alanine and Aspartate Metabolism' | U | 4.71E-35 | 0.622030493 |
| 0.298845462 | 2.01E-09 | 'S::Fatty Acid, Monohydroxy' | S | 2.01E-09 | 0.298845462 |
| 0.271567965 | 1.45E-10 | 'S::Fatty Acid, Dicarboxylate' | S | 2.93E-09 | 0.249745966 |
| -0.141370801 | 1.61E-04 | 'S::Leucine, Isoleucine and Valine Metabolism' |  | 1.61E-04 | -0.141370801 |
| -0.134892993 | 1.90E-05 | 'S::Purine Metabolism, (Hypo)Xanthine/Inosine containing' |  | 6.74E-03 | -0.111141029 |

Table S5C. Gender related modules corrected for oral hygiene.
