# Supplementary material for: Phenotype-driven identification of modules in a hierarchical map of multifluid metabolic correlations
Source: NPJ Syst Biol Appl. 2017 Sep 21;3:28. doi: 10.1038/s41540-017-0029-9 (PMC5608949; doi:10.1038/s41540-017-0029-9)
Supplement: Supplementary file 1 — Supplementary Material [file 41540_2017_29_MOESM1_ESM.zip › Supplement_onlineVersion/SupportingInformation_S6_Module identification algorithm.docx]

Phenotype-driven identification of modules in a hierarchical map of multifluid metabolic correlations

#### *Kieu Trinh Do, Maik Pietzner, David Rasp, Nele Friedrich, Matthias Nauck, Thomas Kocher, Karsten Suhre, Dennis O. Mook-Kanamori, Gabi Kastenmüller, Jan Krumsiek*

#### Supporting Information S6: Module identification algorithm

| 1. | 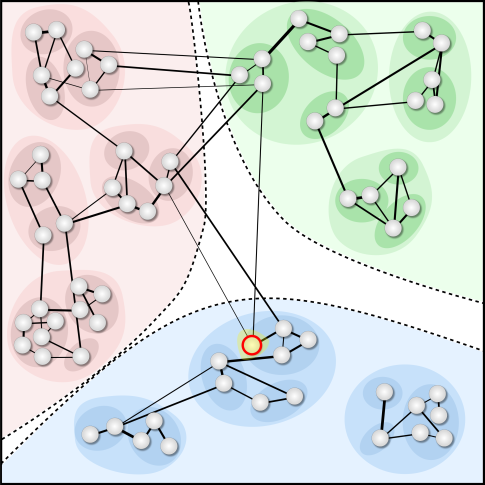 | The algorithm starts with a *seed node* (red circle) as *candidate module* (yellow area). The score of the *candidate module* is the negative logarithmized p-value from a univariate differential analysis with the outcome:  $seed \sim\beta_{seed,0}+ \beta_{seed,1} \cdot P+ \beta_{seed,2}\cdot\mathrm{gender}+ \beta_{seed,3}\cdot\mathrm{age}+ \beta_{seed,4}\cdot\mathrm{BMI}+ \epsilon_{seed}$  Where *seed* is the seed node, $\beta_{seed,0}$ is the intercept, $\beta_{seed,1},\ldots, \beta_{seed,4}$ are the regression coefficients for each independent variable, $P$ is the phenotype of interest and $\epsilon_{seed}$ is a normally distributed error term. |
| --- | --- | --- |
| 2. | 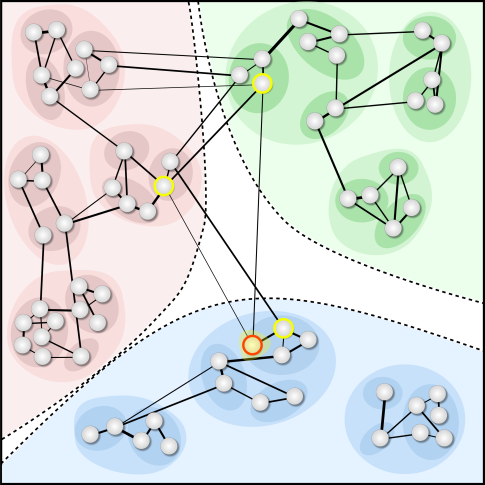 | The neighborhood of the *candidate module* is identified. Each neighbor (yellow circle) is added to the module and the score of the extended module is calculated according to the linear regression model  $R_{M} \sim\beta_{M,0}+ \beta_{M,1} \cdot P+ \beta_{M,2}\cdot\mathrm{gender}+ \beta_{M,3}\cdot\mathrm{age}+ \beta_{M,4}\cdot\mathrm{BMI}+ \epsilon_{M}$  where $M$ is the *candidate module*, $R_{M}$ is the module representative (aggregated z-score), $\beta_{M,0}$ is the intercept, $\beta_{M,1},\ldots, \beta_{M,4}$ are the regression coefficients for each independent variable, $P$ is the phenotype of interest and $\epsilon_{M}$ is a normally distributed error term. |
| 3. | 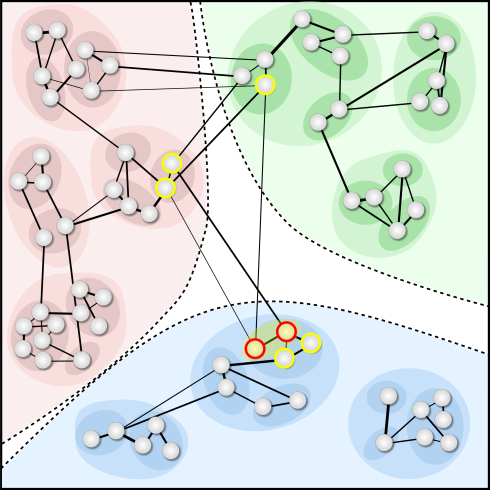 | The neighbor node that improves most the module score is added to the *candidate module* if the module score is higher than the score of each of its single components.  Go to step 2 with the new *candidate module*. |
|  | … |  |
| 4. | 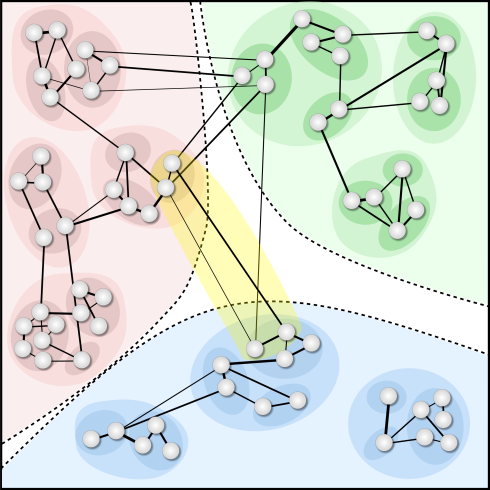 | If no score improvement is possible anymore, the algorithm terminates and an *optimal module* is returned.  Only *optimal modules* with a score higher than the negative logarithmized significance level of 0.05 divided by the number of network nodes (Bonferroni correction for multiple testing) are considered. |
| 5. | 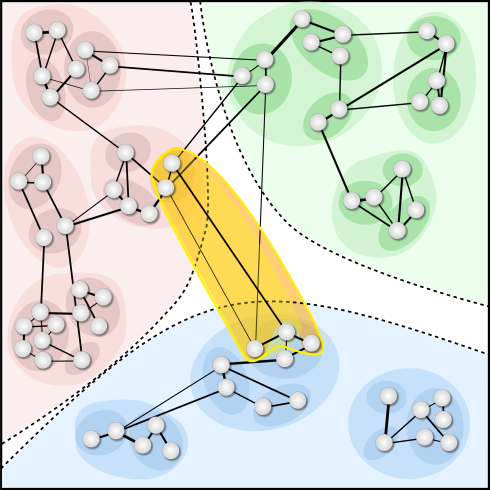 | Overlapping *optimal modules* (e.g., from different seed nodes) are combined into a *maximal module*. |
