# Supplementary material for: Phenotype-driven identification of modules in a hierarchical map of multifluid metabolic correlations
Source: NPJ Syst Biol Appl. 2017 Sep 21;3:28. doi: 10.1038/s41540-017-0029-9 (PMC5608949; doi:10.1038/s41540-017-0029-9)
Supplement: Supplementary file 1 — Supplementary Material [file 41540_2017_29_MOESM1_ESM.zip › Supplement_onlineVersion/SupportingInformation_S6_Module identification algorithm.pdf]

Phenotype-driven identification of modules in a hierarchical map of multifluid metabolic correlations

Kieu Trinh Do, Maik Pietzner, David Rasp, Nele Friedrich, Matthias Nauck, Thomas Kocher, Karsten Suhre, Dennis O. Mook-Kanamori, Gabi Kastenmüller, Jan Krumsiek

Supporting Information S6: Module identification algorithm

1.

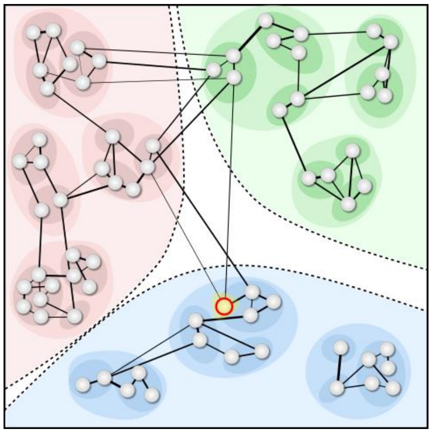

The algorithm starts with a *seed node* (red circle) as *candidate module* (yellow area). The score of the *candidate module* is the negative logarithmized p-value from a univariate differential analysis with the outcome:

$$seed \sim \beta_{seed,0} + \beta_{seed,1} \cdot \mathrm{P} + \beta_{seed,2} \cdot \mathrm{gender} + \beta_{seed,3} \cdot \mathrm{age} + \beta_{seed,4} \cdot \mathrm{BMI} + \epsilon_{seed}$$

Where *seed* is the seed node, $\beta_{seed,0}$ is the intercept, $\beta_{seed,1}, \ldots, \beta_{seed,4}$ are the regression coefficients for each independent variable, P is the phenotype of interest and ϵ_{seed} is a normally distributed error term.

2.

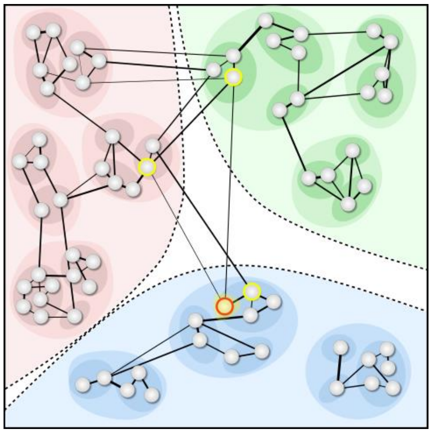

The neighborhood of the *candidate module* is identified. Each neighbor (yellow circle) is added to the module and the score of the extended module is calculated according to the linear regression model

$$R_M \sim \beta_{M,0} + \beta_{M,1} \cdot \mathrm{P} + \beta_{M,2} \cdot \mathrm{gender} + \beta_{M,3} \cdot \mathrm{age} + \beta_{M,4} \cdot \mathrm{BMI} + \epsilon_M$$

where M is the *candidate module*, R_M is the module representative (aggregated z-score), $\beta_{M,0}$ is the intercept, $\beta_{M,1}, \ldots, \beta_{M,4}$ are the regression coefficients for each independent variable, P is the phenotype of interest and ϵ_M is a normally distributed error term.

3.

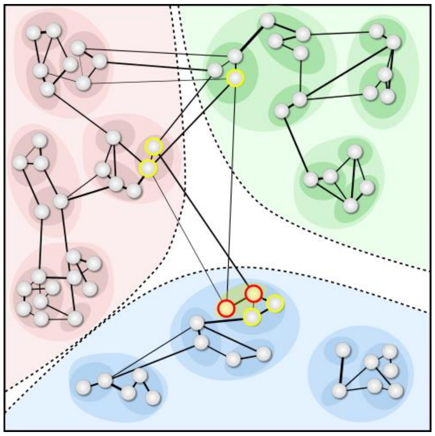

The neighbor node that improves most the module score is added to the *candidate module* if the module score is higher than the score of each of its single components.

Go to step 2 with the new *candidate module*.

…

4.

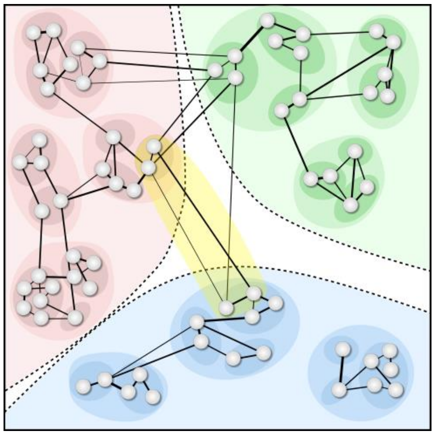

If no score improvement is possible anymore, the algorithm terminates and an *optimal module* is returned.

Only *optimal modules* with a score higher than the negative logarithmized significance level of 0.05 divided by the number of network nodes (Bonferroni correction for multiple testing) are considered.

5.

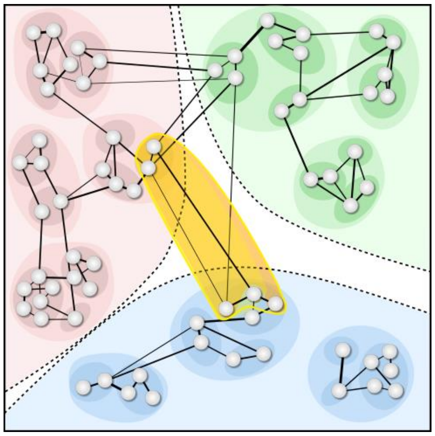

Overlapping *optimal modules* (e.g., from different seed nodes) are combined into a *maximal module*.
