# Supplementary material for: Phenotype-driven identification of modules in a hierarchical map of multifluid metabolic correlations
Source: NPJ Syst Biol Appl. 2017 Sep 21;3:28. doi: 10.1038/s41540-017-0029-9 (PMC5608949; doi:10.1038/s41540-017-0029-9)
Supplement: Supplementary file 1 — Supplementary Material [file 41540_2017_29_MOESM1_ESM.zip › Supplement_onlineVersion/SupportingInformation_S9_QMDiab replication.pdf]

Phenotype-driven identification of modules in a hierarchical map of multifluid metabolic correlations

Kieu Trinh Do, Maik Pietzner, David Rasp, Nele Friedrich, Matthias Nauck, Thomas Kocher, Karsten Suhre, Dennis O. Mook-Kanamori, Gabi Kastenmüller, Jan Krumsiek

Supporting Information S9: Replication of gender associated modules in the Qatar Metabolomics Study on Diabetes (QMDiab)

Metabolites in SHIP-TREND were measured with LC/MS, while the measurements in QMDiab were performed with both LC/MS and GC/MS. This results in considerable variation in the number of metabolites quantified, and therefore in variation in heterogeneity between the same pathways of the two cohorts. Thus, we only considered metabolites measured in both cohorts for an appropriate comparison and generated a new hierarchical map based on the reduced SHIP-TREND dataset comprising 752 metabolites in total (490 known and 262 unknown), which are grouped into 134 different sub-pathways (unknowns excluded).

The module search algorithm was run on this newly generated hierarchical map for both SHIP-TREND and QMDiab. Specifically, we performed the module identification for QMDiab by using differential statistics form QMDiab, but the network from SHIP (i.e. we did not recalculate the hierarchical map on QMDiab data).

In the reduced SHIP-TREND dataset, nine modules at sub-pathway level were found to be significantly associated with gender, while in QMDiab four modules were identified in total. We defined a SHIP-TREND module as replicated if the majority (>50%) of it was also captured in a QMDiab module. Three SHIP-TREND modules were replicated in QMDiab. The majority of SHIP-TREND module 5 comprising plasma amino acid and peptide pathways was found in QMDiab module 1 (Table S). Four out of seven pathways in SHIP-TREND module 6, consisting of urinary nucleotide, energy and amino acid pathways, formed a gender associated module (module 2) in QMDiab. Finally, module 7, containing salivary fatty acids in SHIP-TREND, was completely captured in module 3 of QMDiab, which additionally contains salivary 'Food Component/Plant'. While in SHIP-TREND we identified several cross-fluid modules, all modules in QMDiab consisted of pathways from the same bodyfluid. In addition, we observed

substantially lower p-values of sub-pathway gender associations in SHIP-TREND compared to in QMDiab probably due to higher sample sizes in SHIP-TREND. This resulted in less identified modules in QMDiab.

Cohort	Module	Sub-pathway	Sub-pathway p-value	Module score
SHIP-TREND	module 1	P::Monoacylglycerol	7.65×10^{-6}	1.16×10^{-13}
		P::Lysolipid	1.62×10^{-10}	
		P::Fatty Acid Metabolism(Acyl Carnitine)	2.49×10^{-10}	
	module 2	P::Long Chain Fatty Acid	2.27×10^{-16}	1.61×10^{-16}
		P::Fatty Acid, Branched	8.71×10^{-13}	
		P::Medium Chain Fatty Acid	1.91×10^{-9}	
	module 3	P::Steroid	4.73×10^{-41}	5.34×10^{-47}
		U::Steroid	3.45×10^{-34}	
	module 4	P::Tryptophan Metabolism	5.66×10^{-8}	1.35×10^{-11}
		P::Phenylalanine and Tyrosine Metabolism	6.06×10^{-11}	
	module 5	P::Fatty Acid Metabolism (also BCAA Metabolism)	2.55×10^{-24}	1.45×10^{-84}
		P::Leucine, Isoleucine and Valine Metabolism	4.00×10^{-62}	
		P::Gamma-glutamyl Amino acid	3.28×10^{-62}	
		P::Glutathione Metabolism	1.98×10^{-15}	
		P::Glutamate Metabolism	2.20×10^{-41}	
	module 6	U::Pyrimidine Metabolism, Uracil containing	6.51×10^{-15}	1.37×10^{-50}
		U::TCA Cycle	1.38×10^{-25}	
		U::Oxidative Phosphorylation	3.61×10^{-17}	
		U::Purine Metabolism, Adenine containing	2.74×10^{-1}	
		U::Glutamate Metabolism	1.17×10^{-25}	
		U::Alanine and Aspartate Metabolism	3.52×10^{-36}	
		P::TCA Cycle	2.30×10^{-4}	
	module 7	S::Fatty Acid, Monohydroxy	3.16×10^{-8}	6.35×10^{-10}
		S::Fatty Acid, Dicarboxylate	1.30×10^{-9}	
	module 8	S::Leucine, Isoleucine and Valine Metabolism	3.98×10^{-5}	3.50×10^{-6}
		S::Purine Metabolism, (Hypo)Xanthine/Inosine containing	6.57×10^{-3}	
	module 9	S::Glutathione Metabolism	2.90×10^{-2}	2.32×10^{-7}
		S::Histidine Metabolism	6.15×10^{-3}	
		S::Disaccharides and Oligosaccharides	0.837156322	
		S::TCA Cycle	4.39×10^{-1}	
		S::Urea cycle; Arginine and Proline Metabolism	0.011072867	
		S::Glutamate Metabolism	8.29×10^{-7}	
		S::Chemical	0.322909755	
QMDiab	module 1	P::Leucine, Isoleucine and Valine Metabolism	1.26×10^{-12}	5.48×10^{-16}

		P::Gamma-glutamyl Amino acid	1.20×10^{-8}	
		P::Glutathione Metabolism	0.000760109	
		P::Glutamate Metabolism	3.70×10^{-13}	
	module 2	U::Purine Metabolism, Adenine containing	7.46×10^{-3}	2.17×10^{-6}
		U::Glutamate Metabolism	1.09×10^{-3}	
		U::Alanine and Aspartate Metabolism	0.000384823	
		U::Oxidative Phosphorylation	2.68×10^{-1}	
	module 3	S::Fatty Acid, Dicarboxylate	1.93×10^{-5}	2.10×10^{-7}
		S::Food Component/Plant	1.76×10^{-5}	
		S::Fatty Acid, Monohydroxy	3.60×10^{-6}	
	module 4	S::Carnitine Metabolism	0.013919067	7.27×10^{-9}09
		S::Creatine Metabolism	4.58×10^{-6}	
		S::Fatty Acid Metabolism(Acyl Carnitine)	0.013110993	
		S::Fatty Acid Metabolism (also BCAA Metabolism)	9.12×10^{-8}	

Table S9. Modules identified for SHIP-TREND and QMDiab. Modules replicated in QMDiab were marked as red, blue, and green.

One further factor accounting for the difference between SHIP-TREND and QMDian was the differing number of samples, that is, the power of the cohorts. SHIP included 906 individuals with metabolomics measurement of all three bodyfluids, whereas QMDiab comprised a total of 372 participants. Finally, SHIP-TREND and QMDiab also differed in the study design. While in SHIP-TREND samples of fasting individuals were collected, in QMDiab the participants were non-fasting. The SHIP-TREND was conducted in West Pomerania, Germany, while the individuals in the QMDiab cohort were mainly of Arab and Asian ethnicity. Moreover, in contrast to SHIP-TREND which was designed as a healthy cohort, QMDiab is a case-control study for type 2 diabetes.
