# Supplementary figures and images for: Phenotype-driven identification of modules in a hierarchical map of multifluid metabolic correlations
Source: NPJ Syst Biol Appl. 2017 Sep 21;3:28. doi: 10.1038/s41540-017-0029-9 (PMC5608949; doi:10.1038/s41540-017-0029-9)

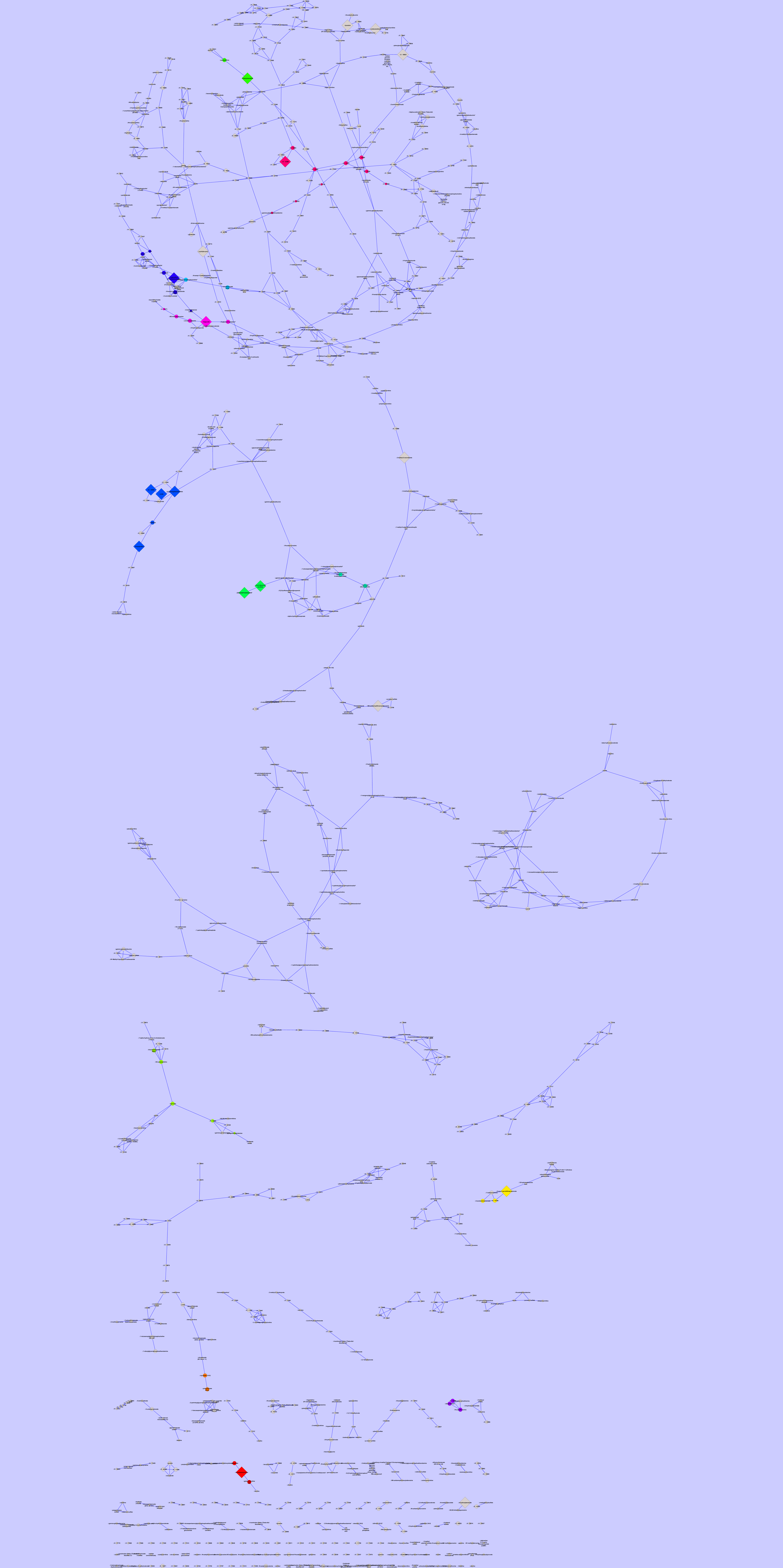

Supplement: Supplementary file 1 — Supplementary Material [file 41540_2017_29_MOESM1_ESM.zip › Supplement_onlineVersion/SupportingInformation_S10_Module identification code/ModuleIdentification/Results/levelMet/Simulated_modules_levelMet.png]

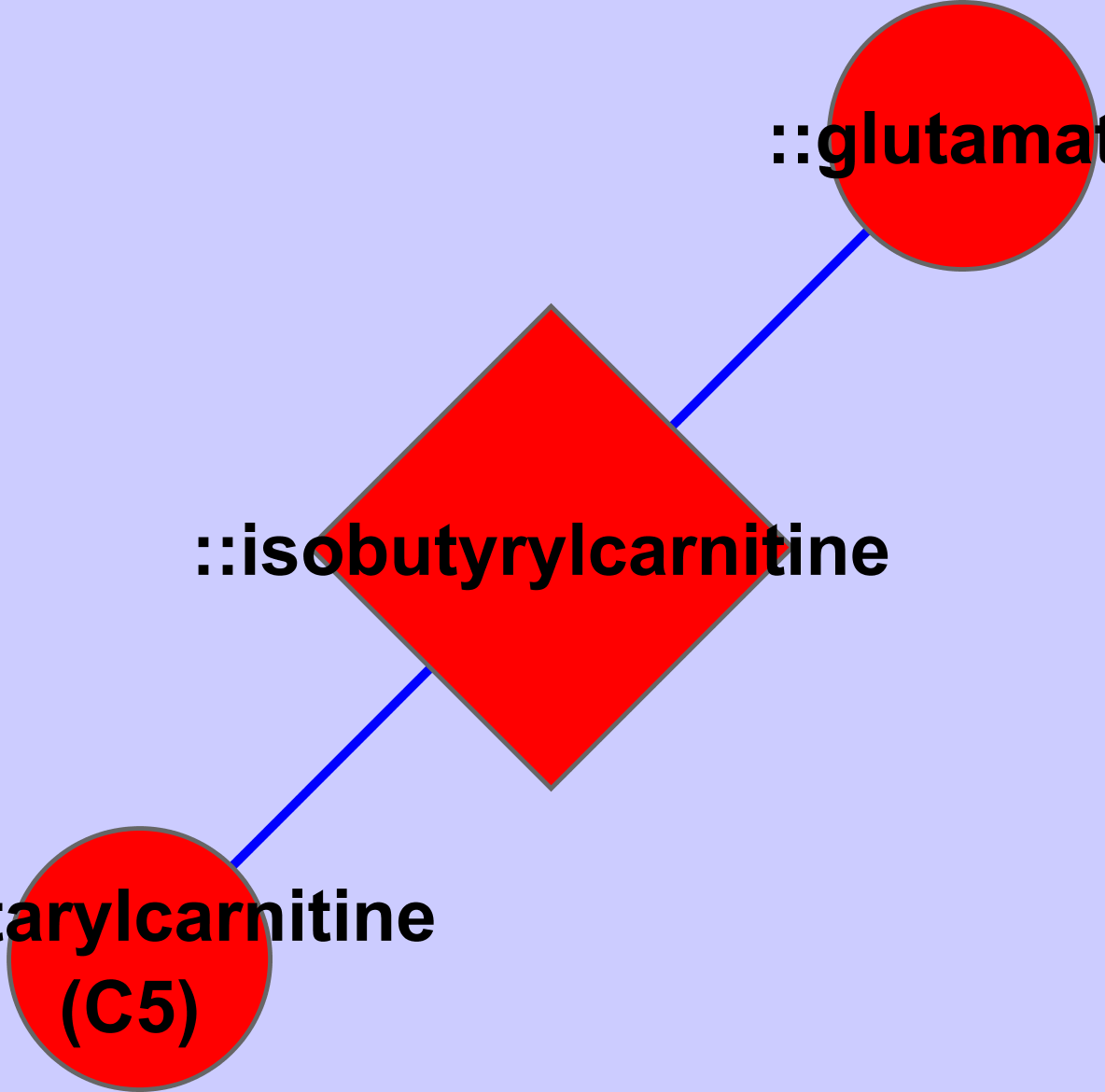

Supplement: Supplementary file 1 — Supplementary Material [file 41540_2017_29_MOESM1_ESM.zip › Supplement_onlineVersion/SupportingInformation_S10_Module identification code/ModuleIdentification/Results/levelMet/Simulated_modules_levelMet_module1.png]

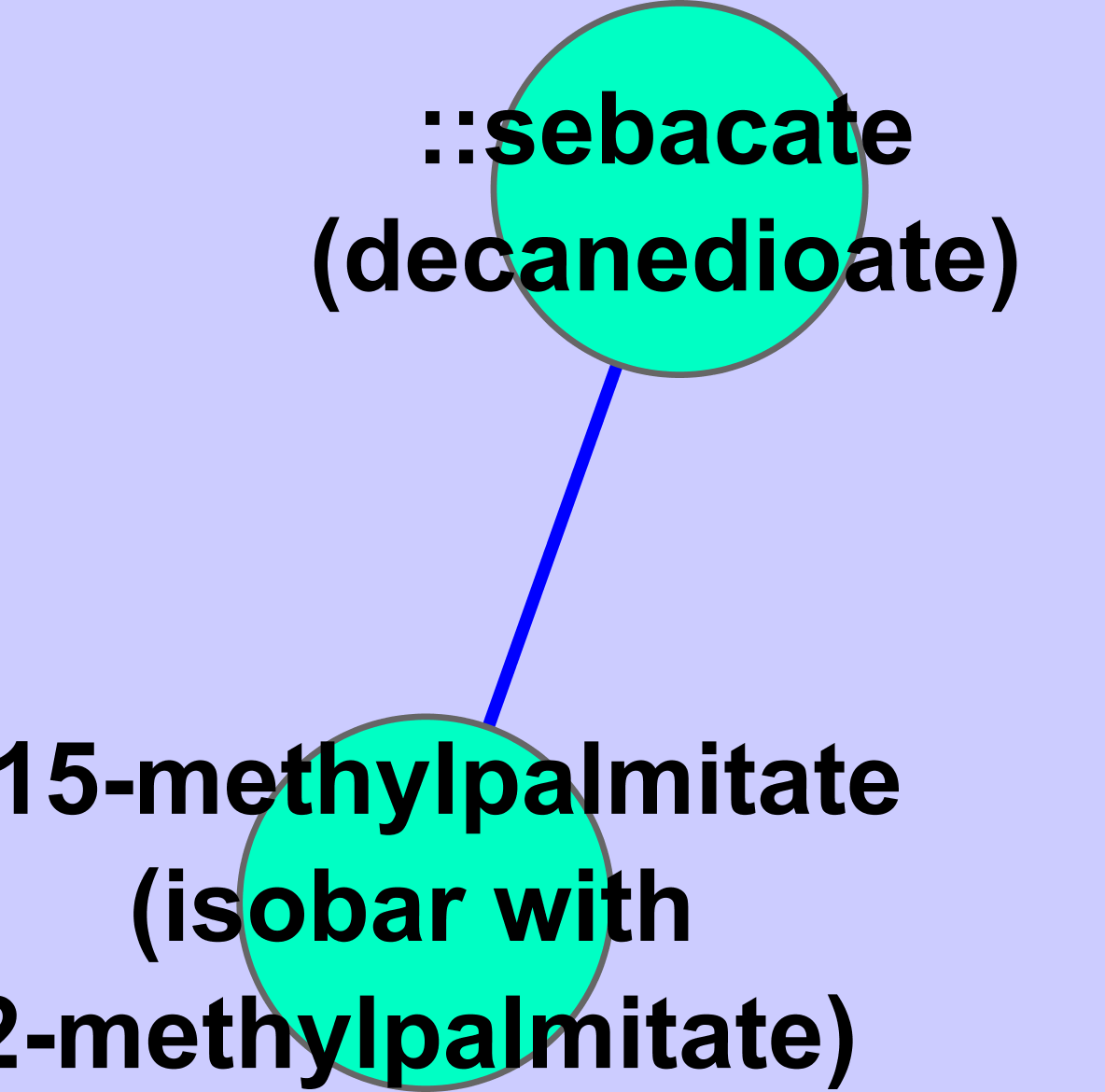

Supplement: Supplementary file 1 — Supplementary Material [file 41540_2017_29_MOESM1_ESM.zip › Supplement_onlineVersion/SupportingInformation_S10_Module identification code/ModuleIdentification/Results/levelMet/Simulated_modules_levelMet_module10.png]

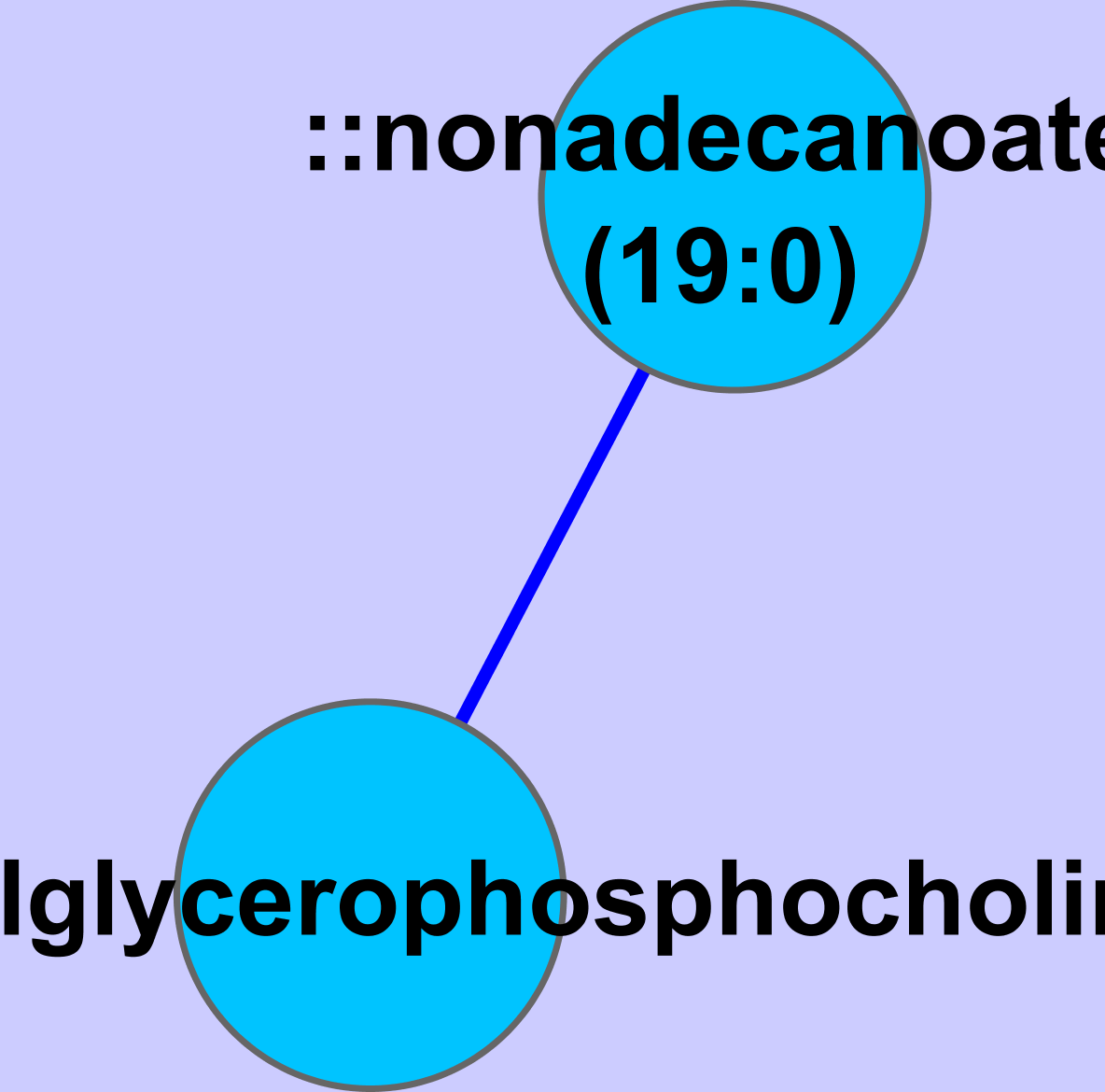

Supplement: Supplementary file 1 — Supplementary Material [file 41540_2017_29_MOESM1_ESM.zip › Supplement_onlineVersion/SupportingInformation_S10_Module identification code/ModuleIdentification/Results/levelMet/Simulated_modules_levelMet_module12.png]

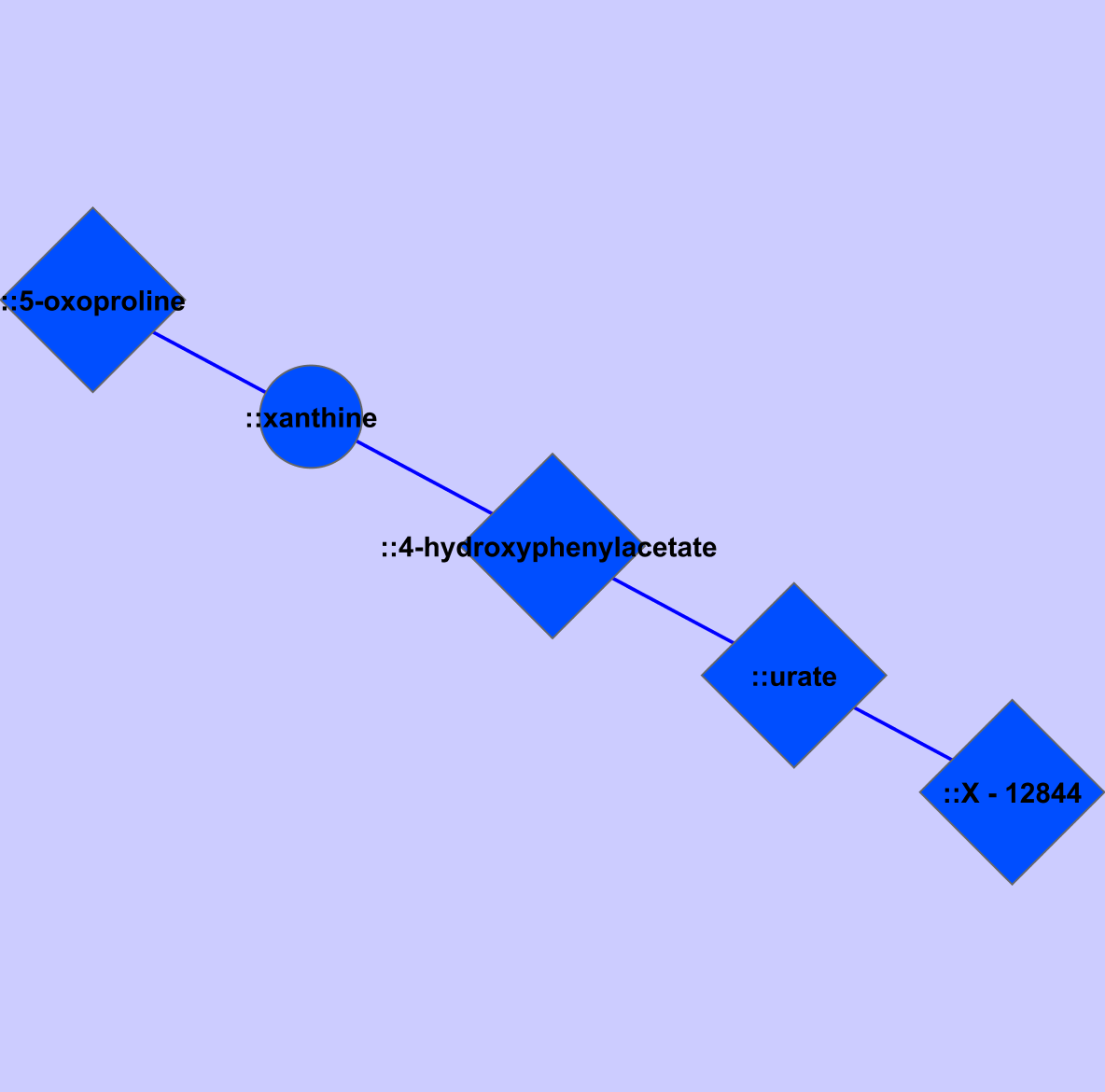

Supplement: Supplementary file 1 — Supplementary Material [file 41540_2017_29_MOESM1_ESM.zip › Supplement_onlineVersion/SupportingInformation_S10_Module identification code/ModuleIdentification/Results/levelMet/Simulated_modules_levelMet_module17.png]

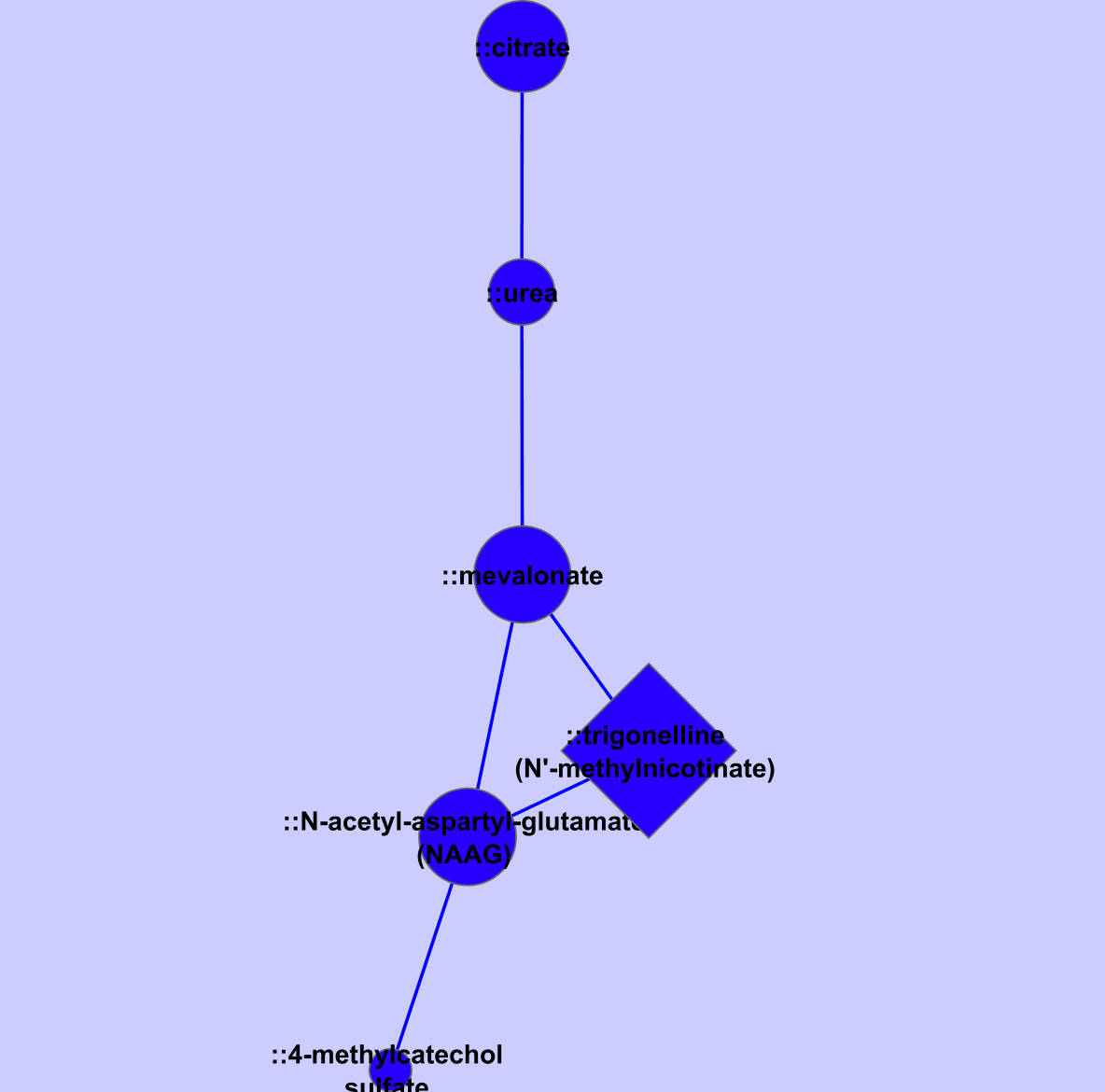

Supplement: Supplementary file 1 — Supplementary Material [file 41540_2017_29_MOESM1_ESM.zip › Supplement_onlineVersion/SupportingInformation_S10_Module identification code/ModuleIdentification/Results/levelMet/Simulated_modules_levelMet_module19.png]

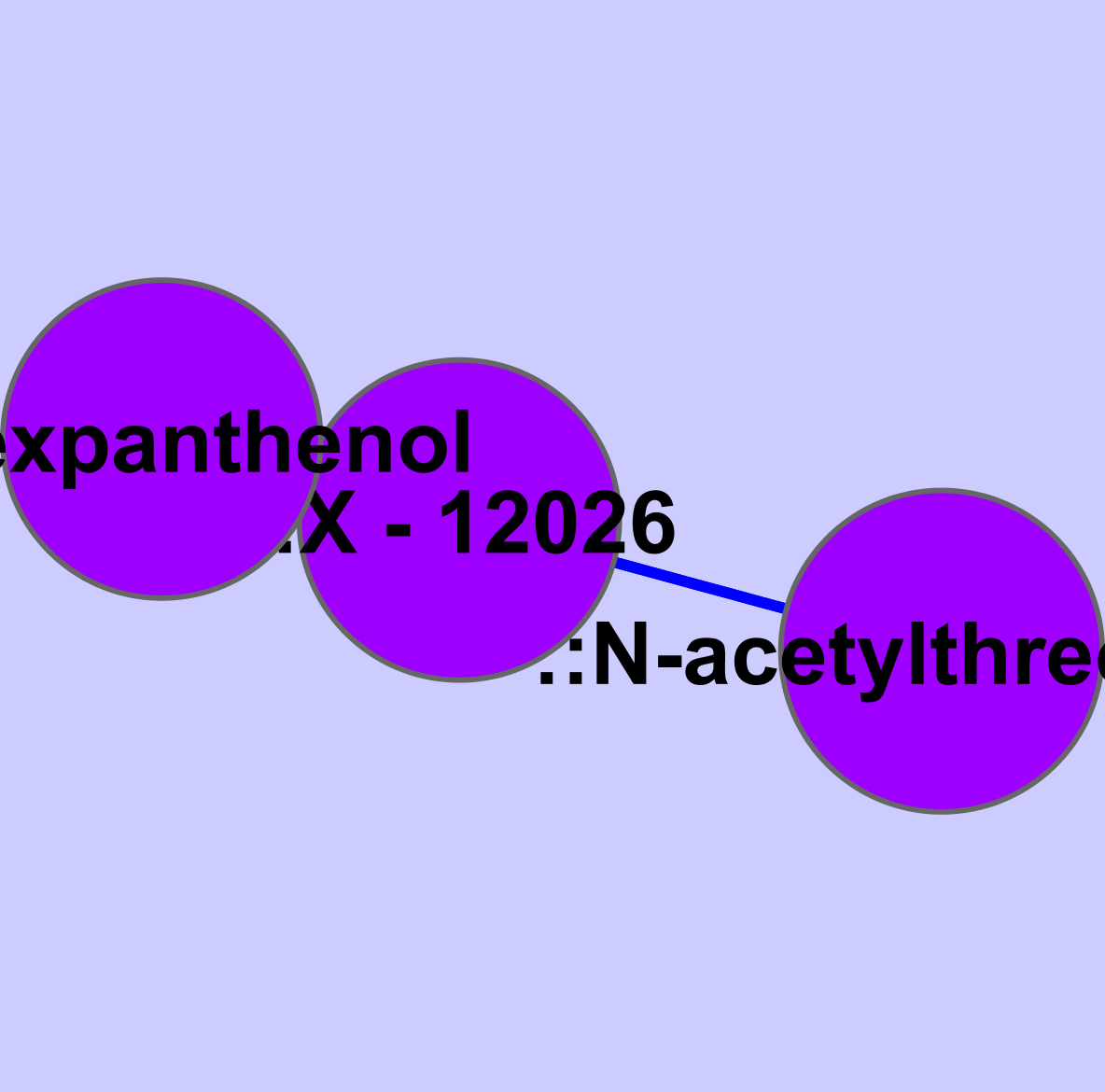

Supplement: Supplementary file 1 — Supplementary Material [file 41540_2017_29_MOESM1_ESM.zip › Supplement_onlineVersion/SupportingInformation_S10_Module identification code/ModuleIdentification/Results/levelMet/Simulated_modules_levelMet_module22.png]

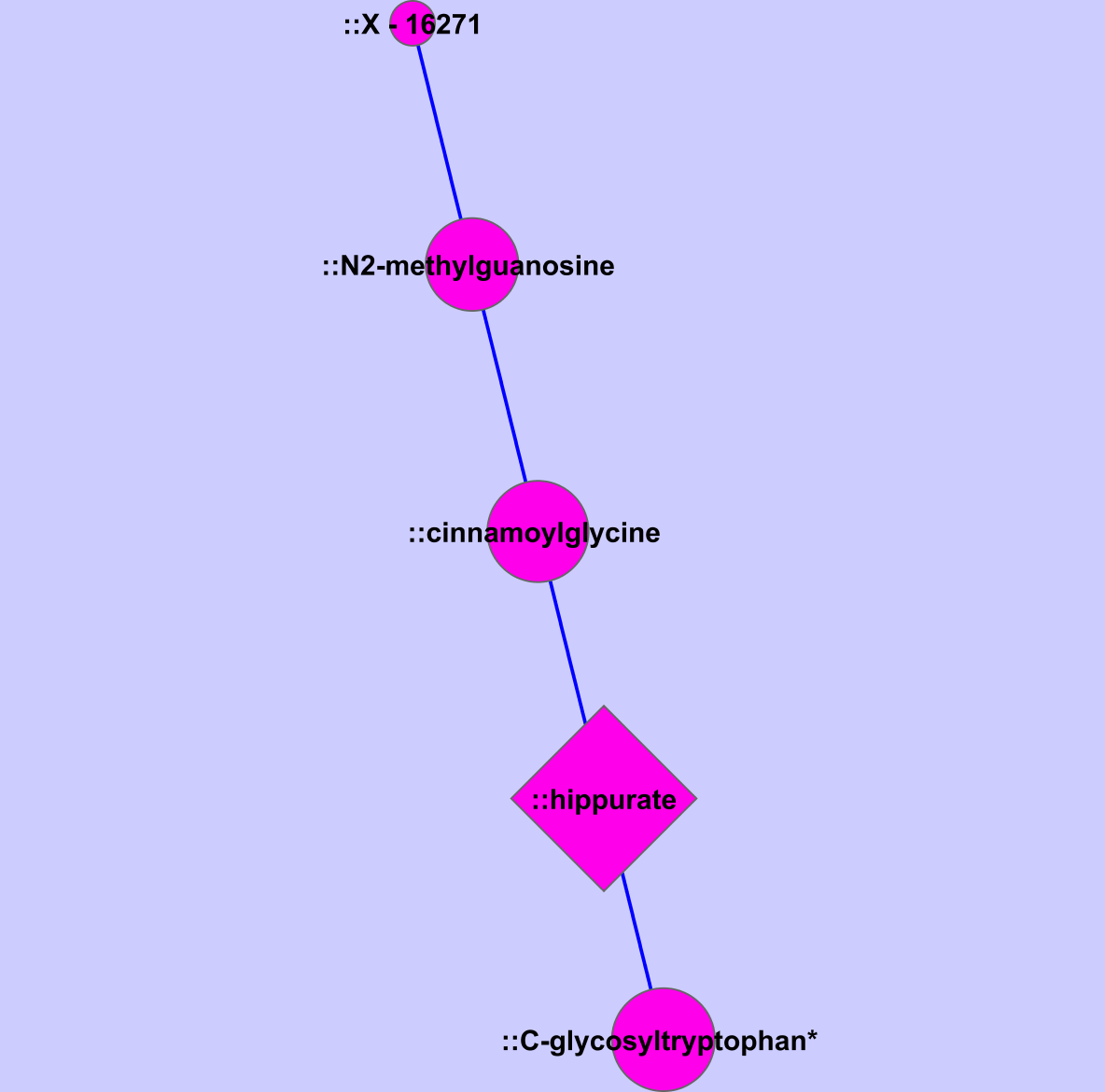

Supplement: Supplementary file 1 — Supplementary Material [file 41540_2017_29_MOESM1_ESM.zip › Supplement_onlineVersion/SupportingInformation_S10_Module identification code/ModuleIdentification/Results/levelMet/Simulated_modules_levelMet_module24.png]

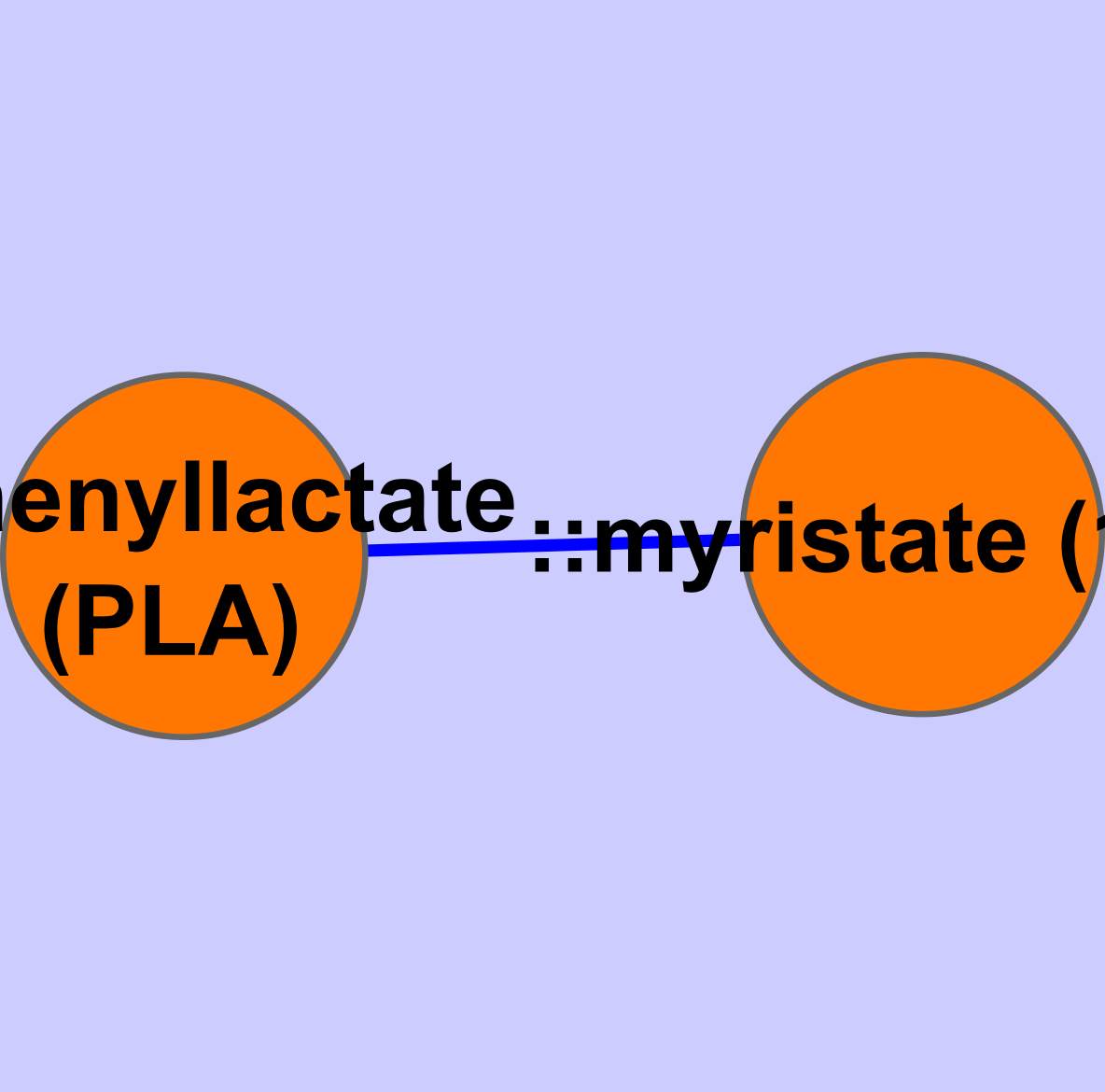

Supplement: Supplementary file 1 — Supplementary Material [file 41540_2017_29_MOESM1_ESM.zip › Supplement_onlineVersion/SupportingInformation_S10_Module identification code/ModuleIdentification/Results/levelMet/Simulated_modules_levelMet_module4.png]

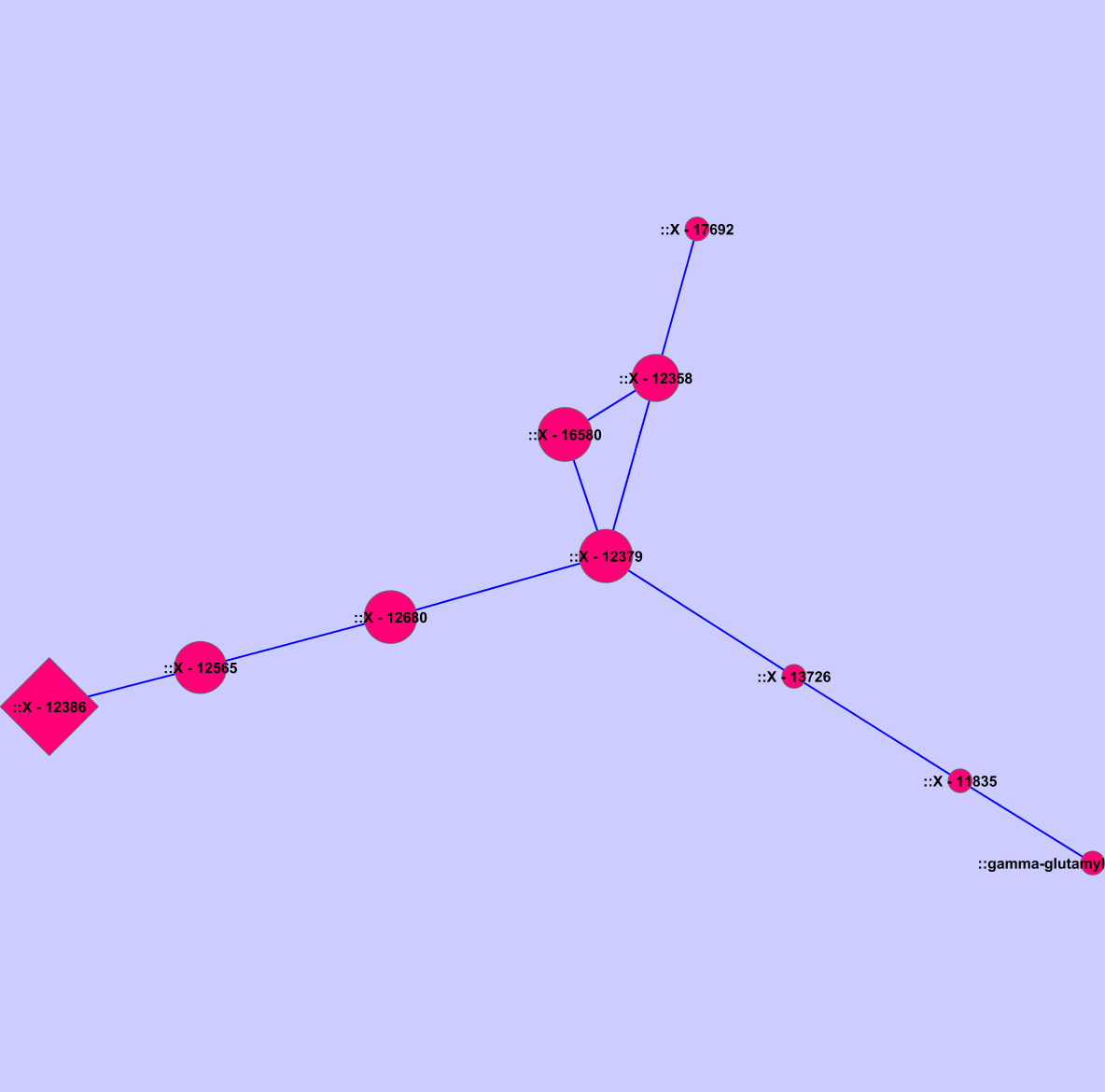

Supplement: Supplementary file 1 — Supplementary Material [file 41540_2017_29_MOESM1_ESM.zip › Supplement_onlineVersion/SupportingInformation_S10_Module identification code/ModuleIdentification/Results/levelMet/Simulated_modules_levelMet_module40.png]

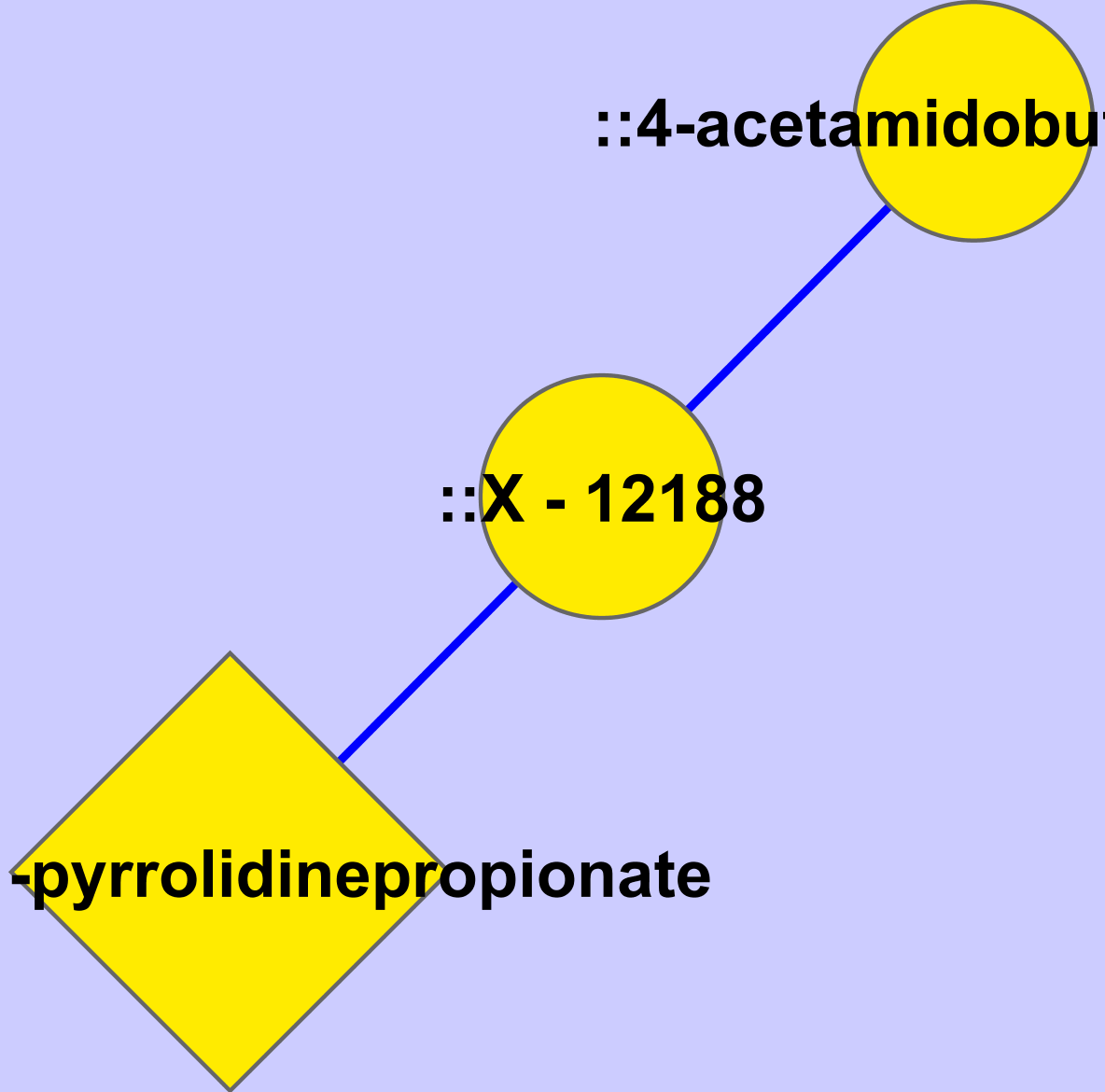

Supplement: Supplementary file 1 — Supplementary Material [file 41540_2017_29_MOESM1_ESM.zip › Supplement_onlineVersion/SupportingInformation_S10_Module identification code/ModuleIdentification/Results/levelMet/Simulated_modules_levelMet_module5.png]

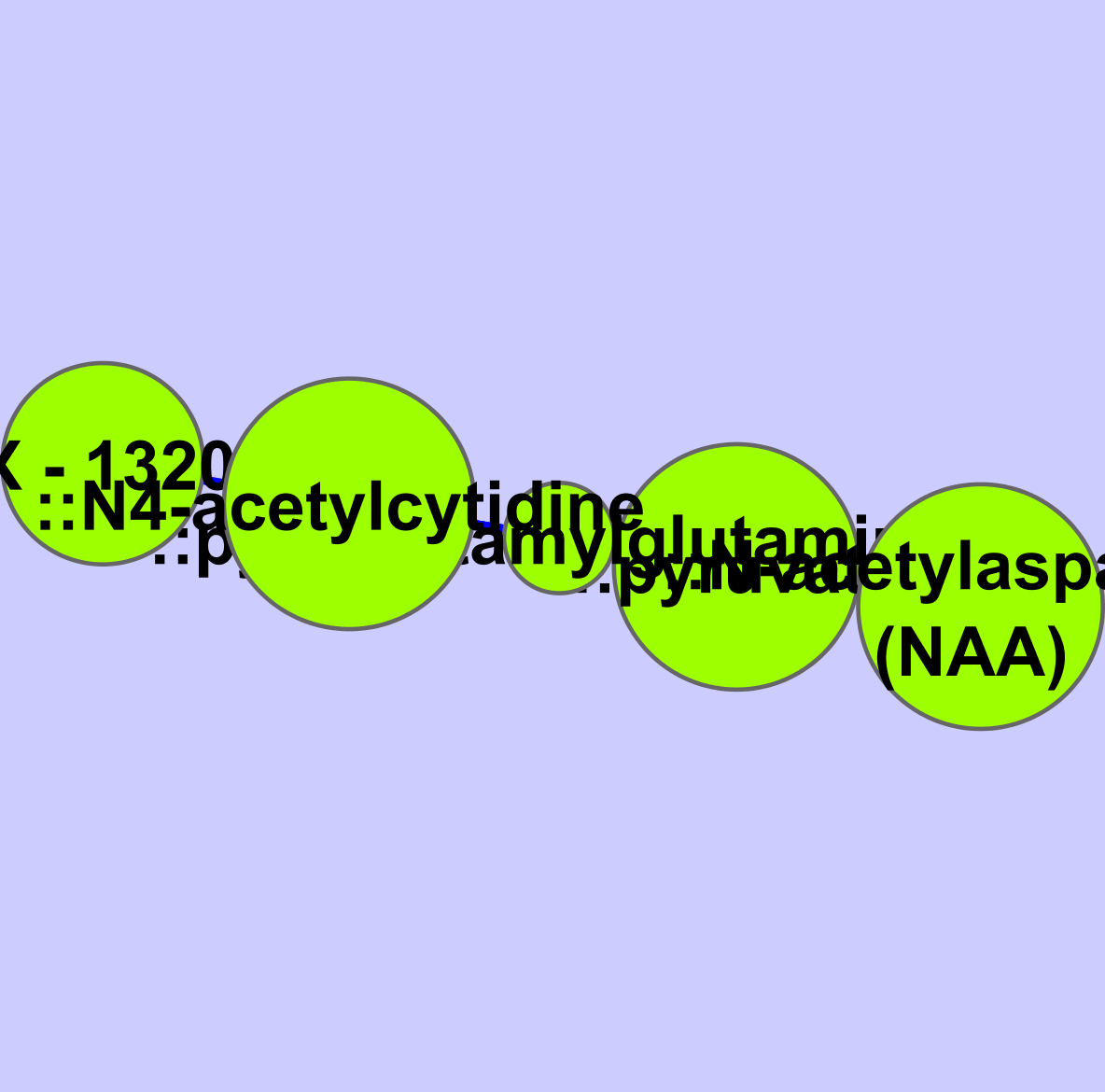

Supplement: Supplementary file 1 — Supplementary Material [file 41540_2017_29_MOESM1_ESM.zip › Supplement_onlineVersion/SupportingInformation_S10_Module identification code/ModuleIdentification/Results/levelMet/Simulated_modules_levelMet_module6.png]

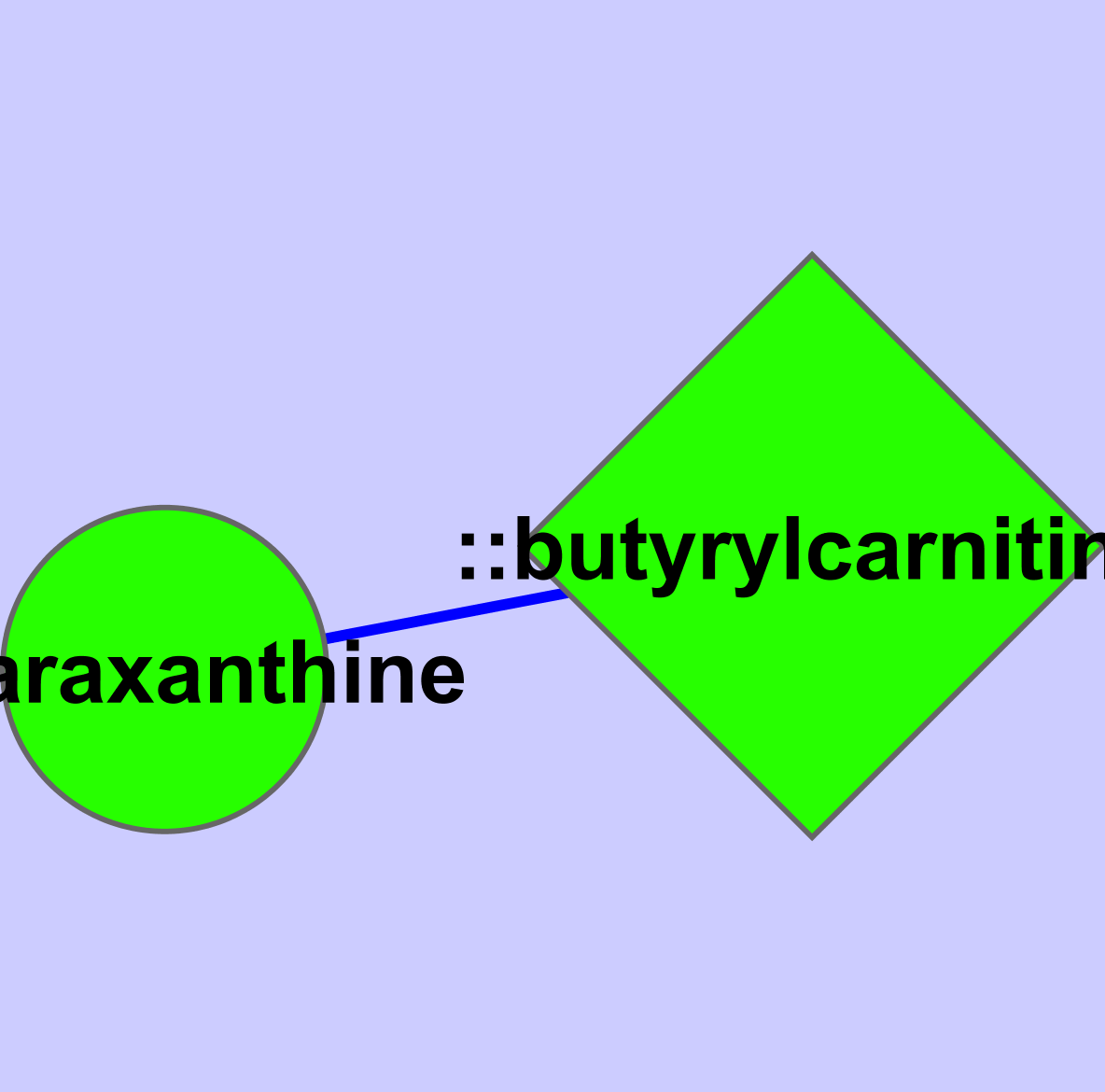

Supplement: Supplementary file 1 — Supplementary Material [file 41540_2017_29_MOESM1_ESM.zip › Supplement_onlineVersion/SupportingInformation_S10_Module identification code/ModuleIdentification/Results/levelMet/Simulated_modules_levelMet_module8.png]

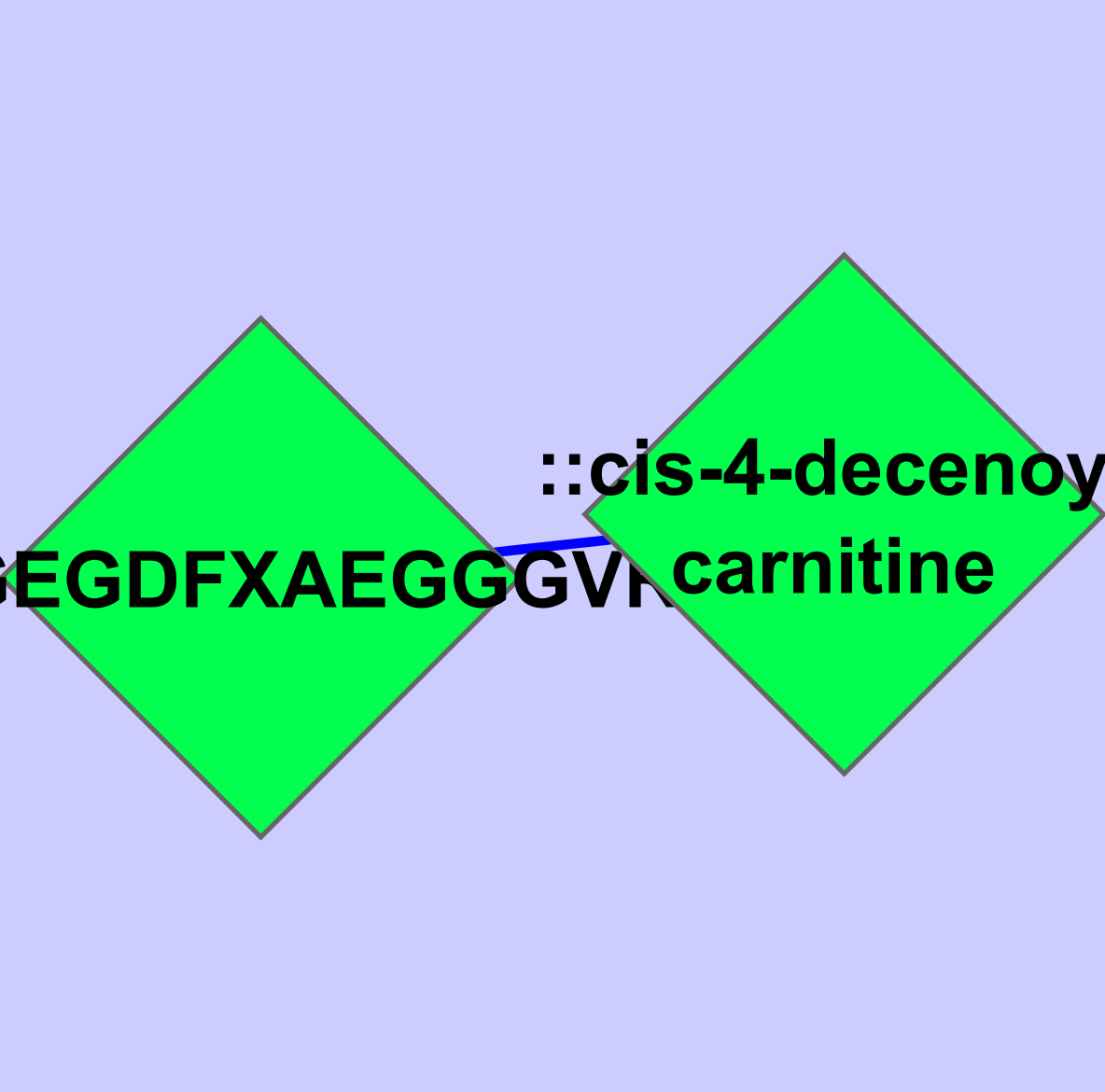

Supplement: Supplementary file 1 — Supplementary Material [file 41540_2017_29_MOESM1_ESM.zip › Supplement_onlineVersion/SupportingInformation_S10_Module identification code/ModuleIdentification/Results/levelMet/Simulated_modules_levelMet_module9.png]

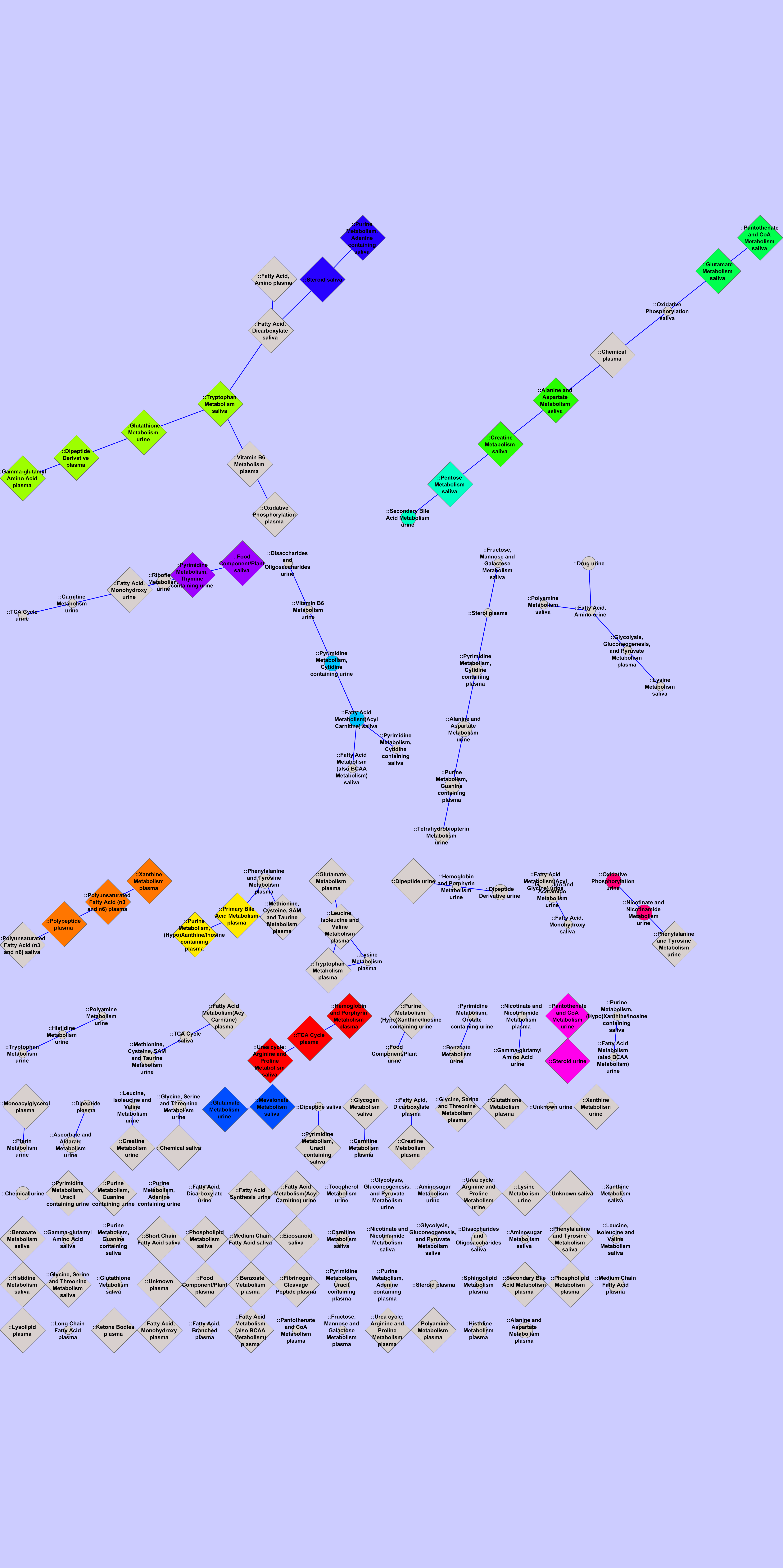

Supplement: Supplementary file 1 — Supplementary Material [file 41540_2017_29_MOESM1_ESM.zip › Supplement_onlineVersion/SupportingInformation_S10_Module identification code/ModuleIdentification/Results/levelSub/Simulated_modules_levelSub.png]

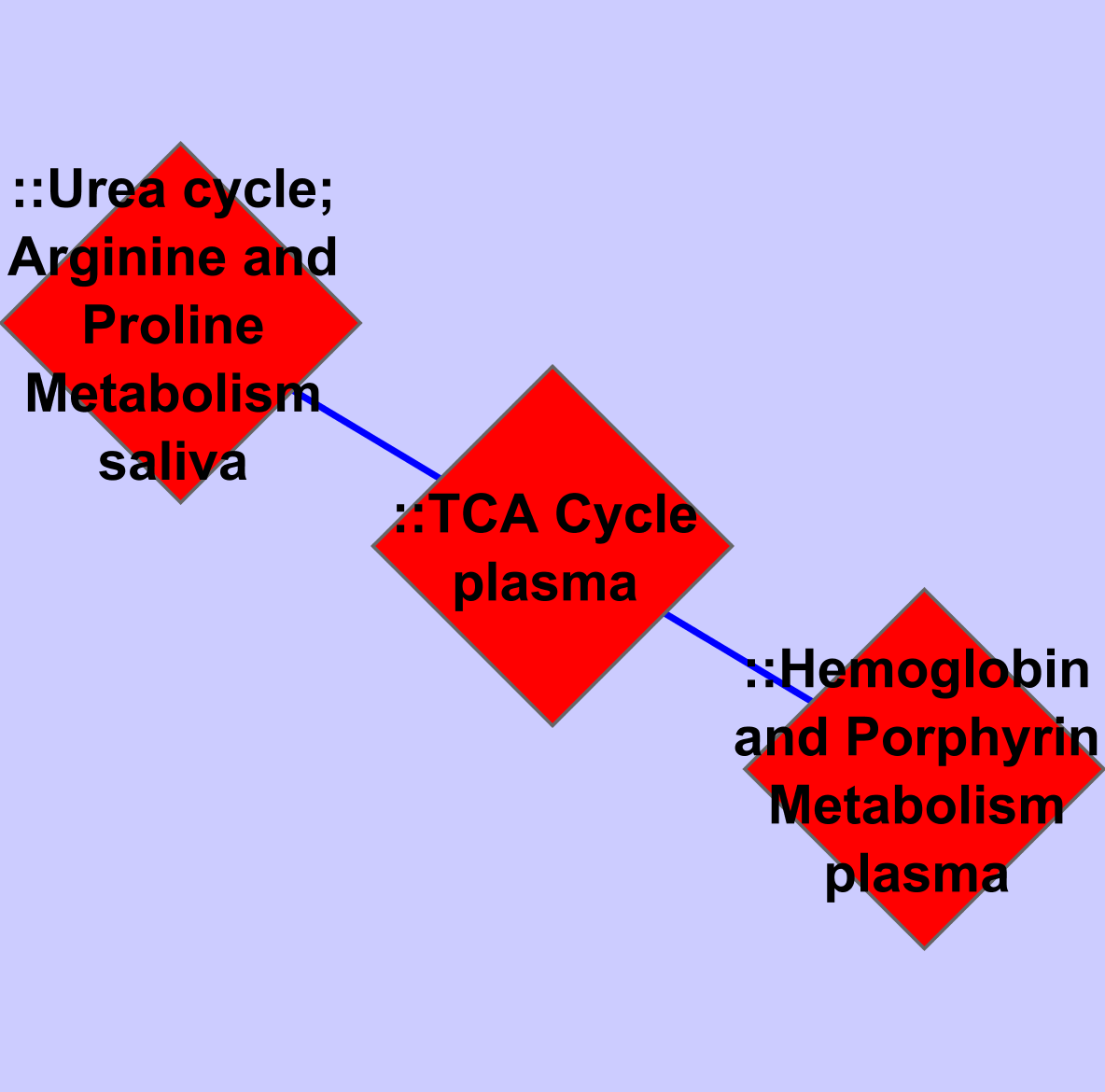

Supplement: Supplementary file 1 — Supplementary Material [file 41540_2017_29_MOESM1_ESM.zip › Supplement_onlineVersion/SupportingInformation_S10_Module identification code/ModuleIdentification/Results/levelSub/Simulated_modules_levelSub_module1.png]

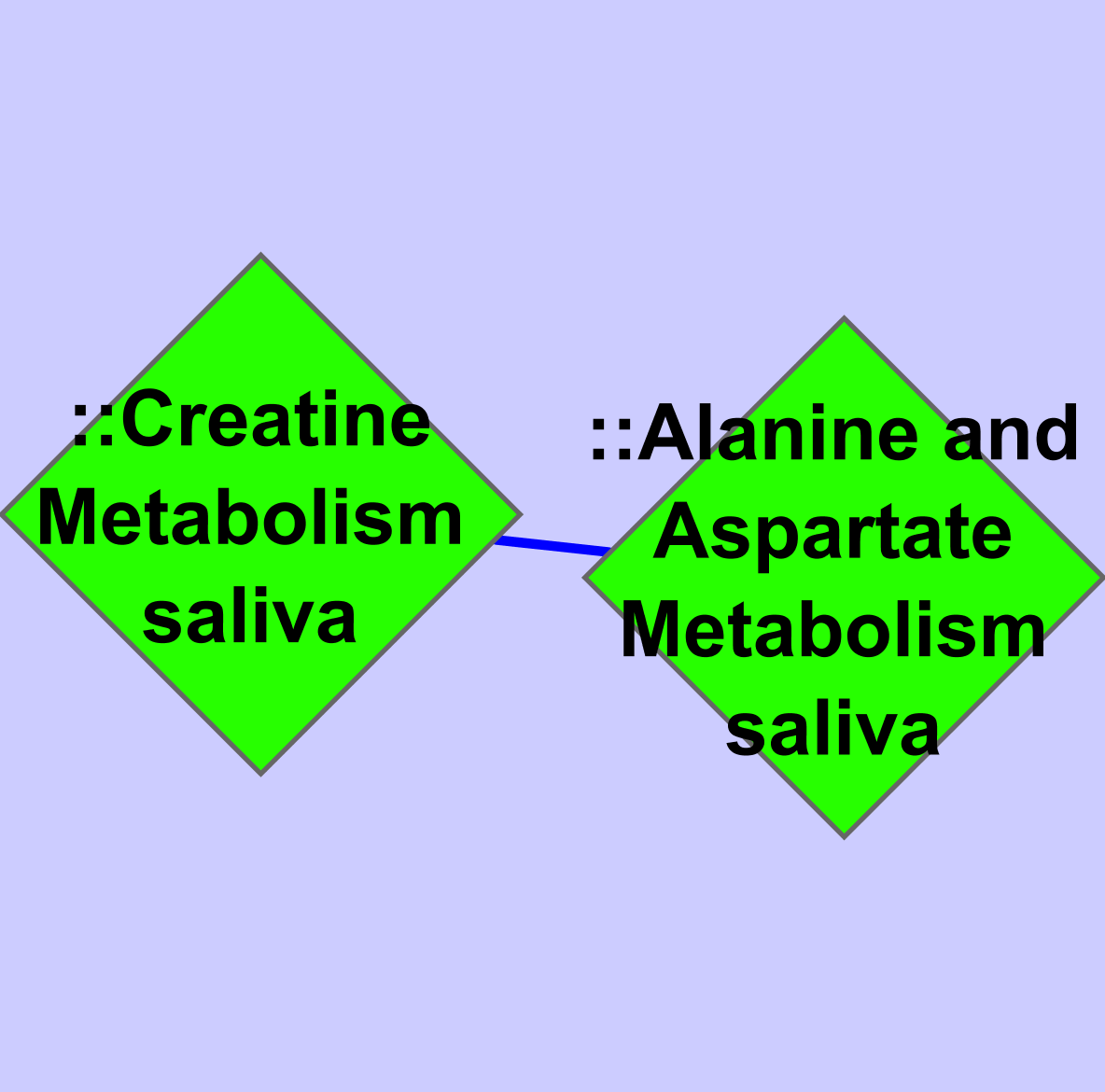

Supplement: Supplementary file 1 — Supplementary Material [file 41540_2017_29_MOESM1_ESM.zip › Supplement_onlineVersion/SupportingInformation_S10_Module identification code/ModuleIdentification/Results/levelSub/Simulated_modules_levelSub_module11.png]

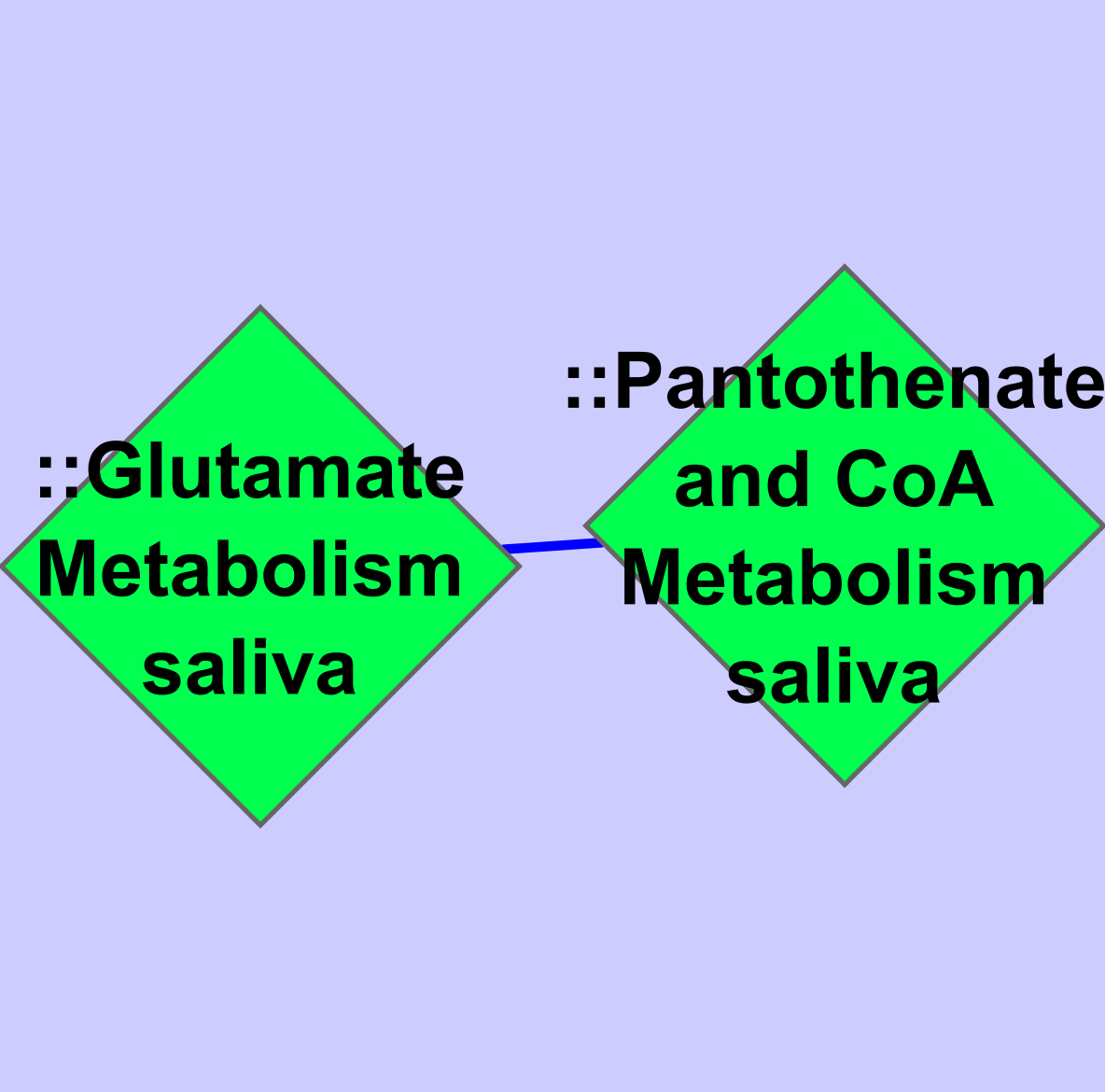

Supplement: Supplementary file 1 — Supplementary Material [file 41540_2017_29_MOESM1_ESM.zip › Supplement_onlineVersion/SupportingInformation_S10_Module identification code/ModuleIdentification/Results/levelSub/Simulated_modules_levelSub_module12.png]

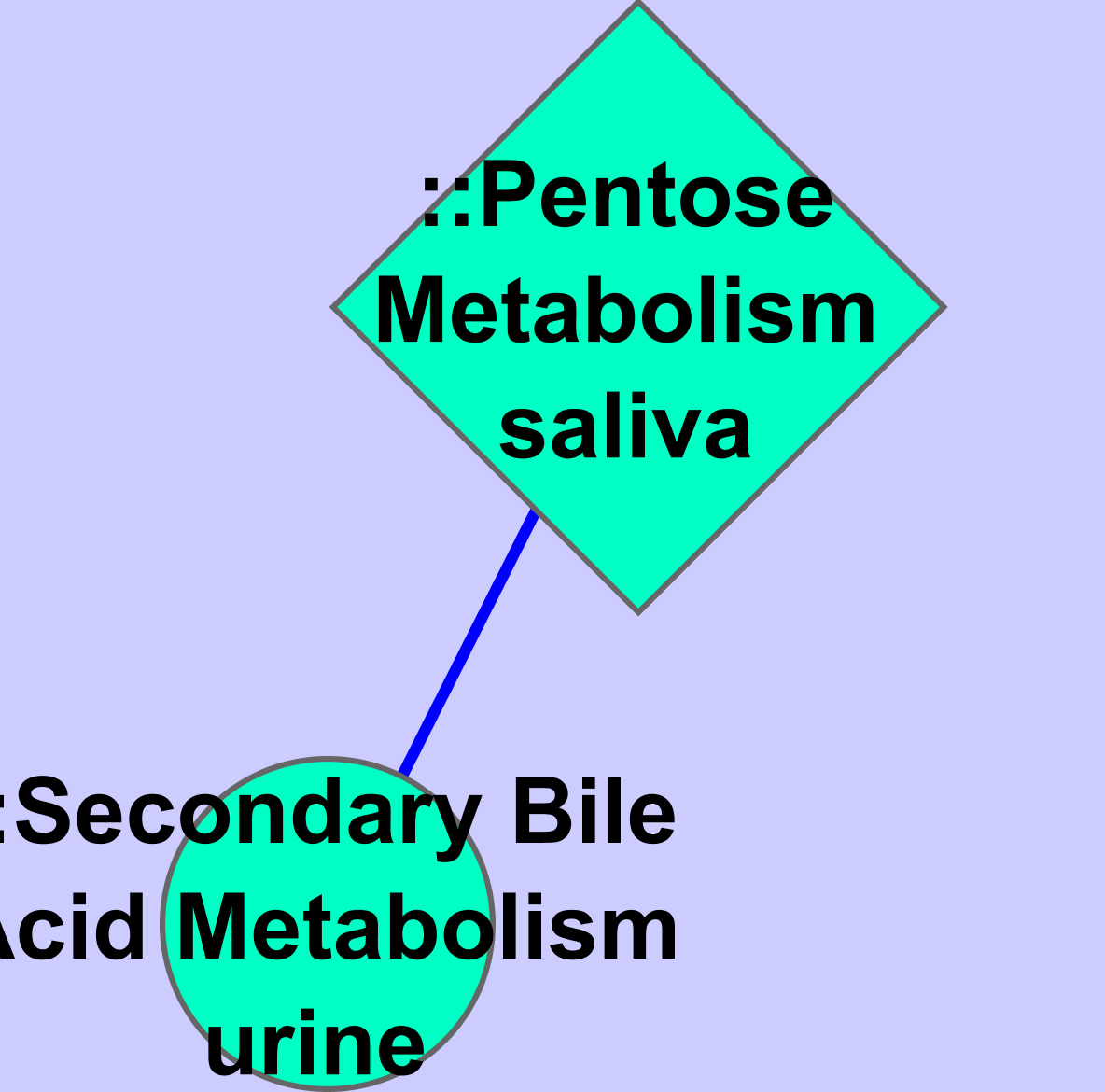

Supplement: Supplementary file 1 — Supplementary Material [file 41540_2017_29_MOESM1_ESM.zip › Supplement_onlineVersion/SupportingInformation_S10_Module identification code/ModuleIdentification/Results/levelSub/Simulated_modules_levelSub_module14.png]

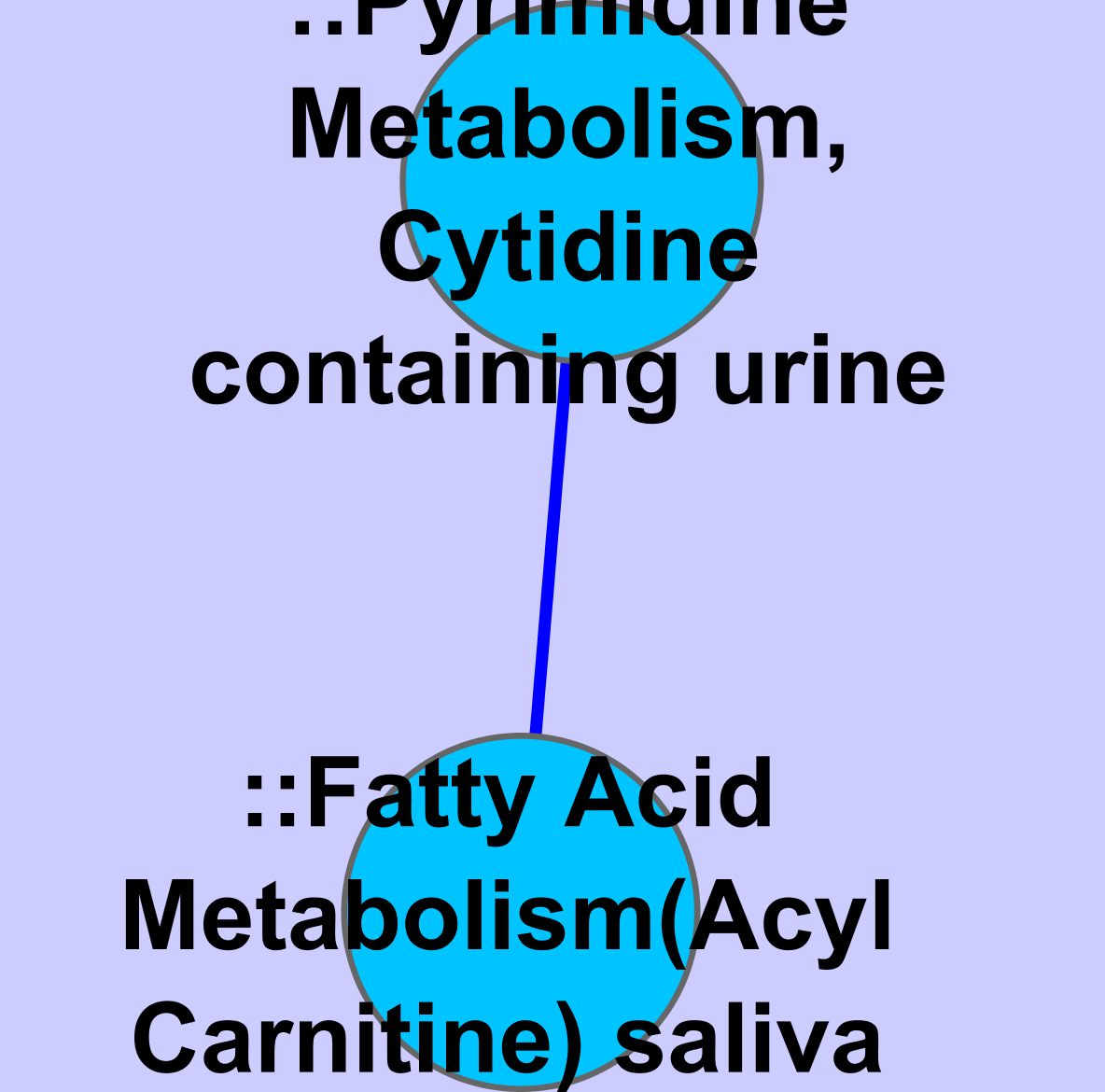

Supplement: Supplementary file 1 — Supplementary Material [file 41540_2017_29_MOESM1_ESM.zip › Supplement_onlineVersion/SupportingInformation_S10_Module identification code/ModuleIdentification/Results/levelSub/Simulated_modules_levelSub_module16.png]

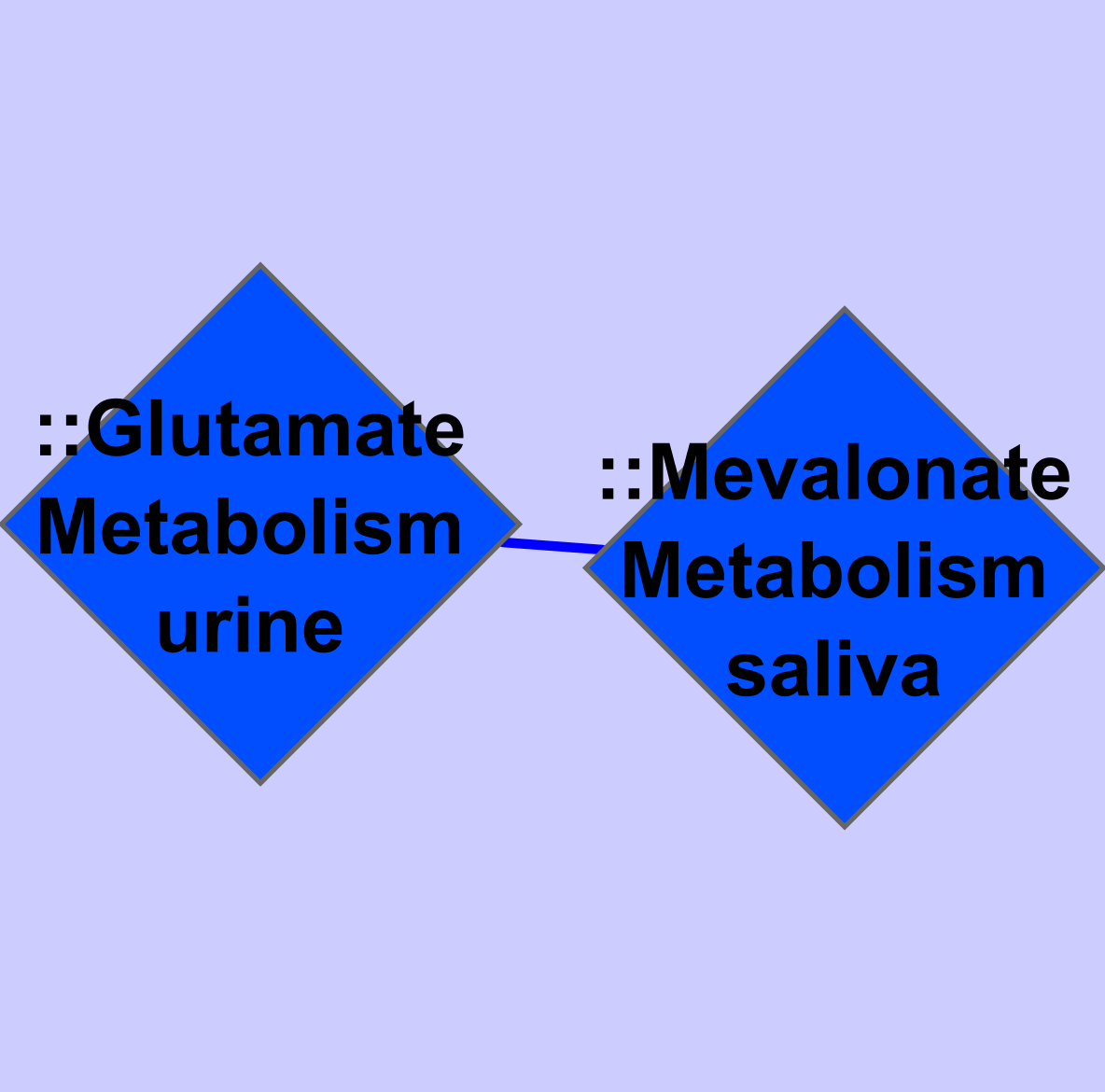

Supplement: Supplementary file 1 — Supplementary Material [file 41540_2017_29_MOESM1_ESM.zip › Supplement_onlineVersion/SupportingInformation_S10_Module identification code/ModuleIdentification/Results/levelSub/Simulated_modules_levelSub_module17.png]

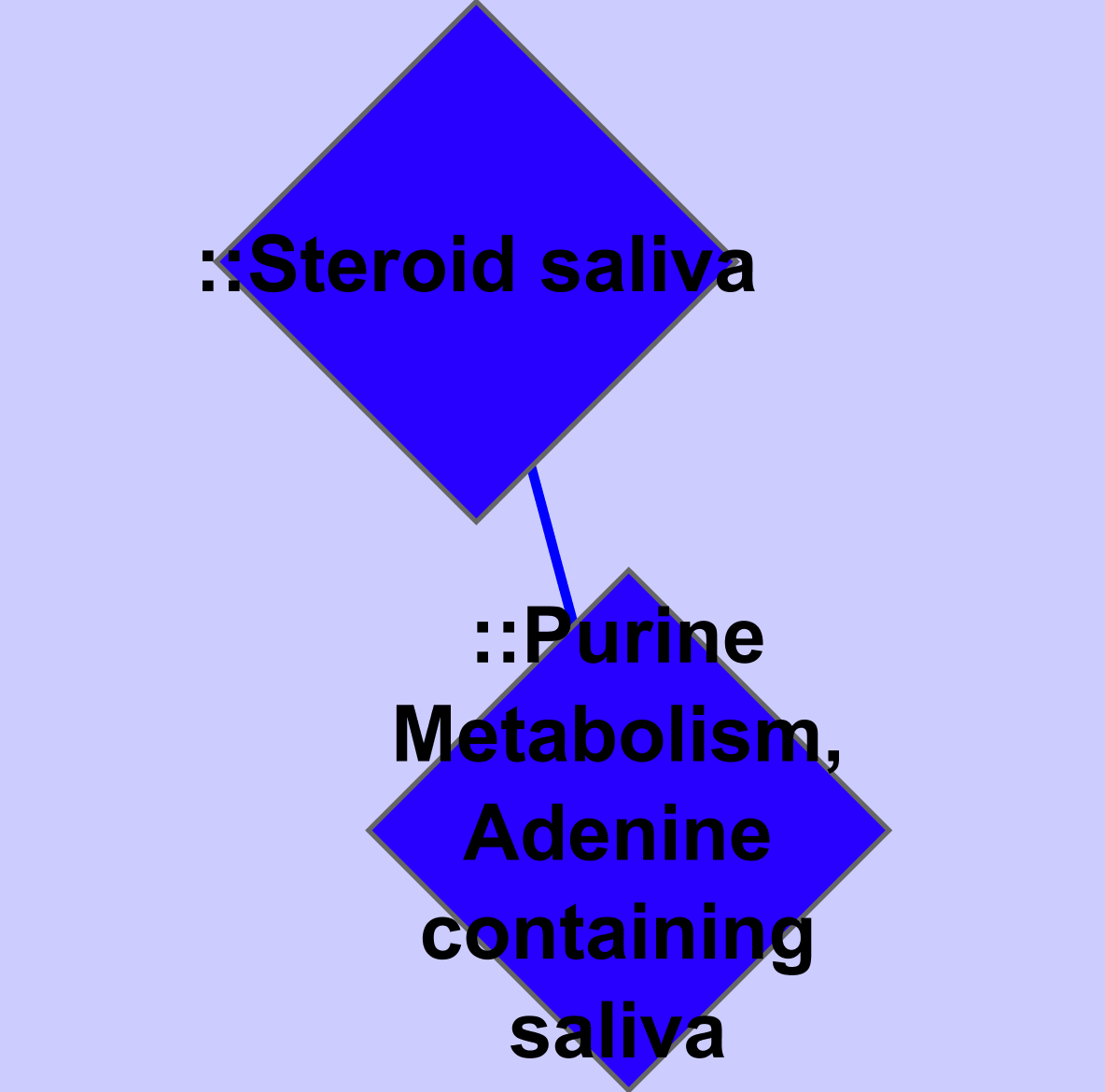

Supplement: Supplementary file 1 — Supplementary Material [file 41540_2017_29_MOESM1_ESM.zip › Supplement_onlineVersion/SupportingInformation_S10_Module identification code/ModuleIdentification/Results/levelSub/Simulated_modules_levelSub_module18.png]

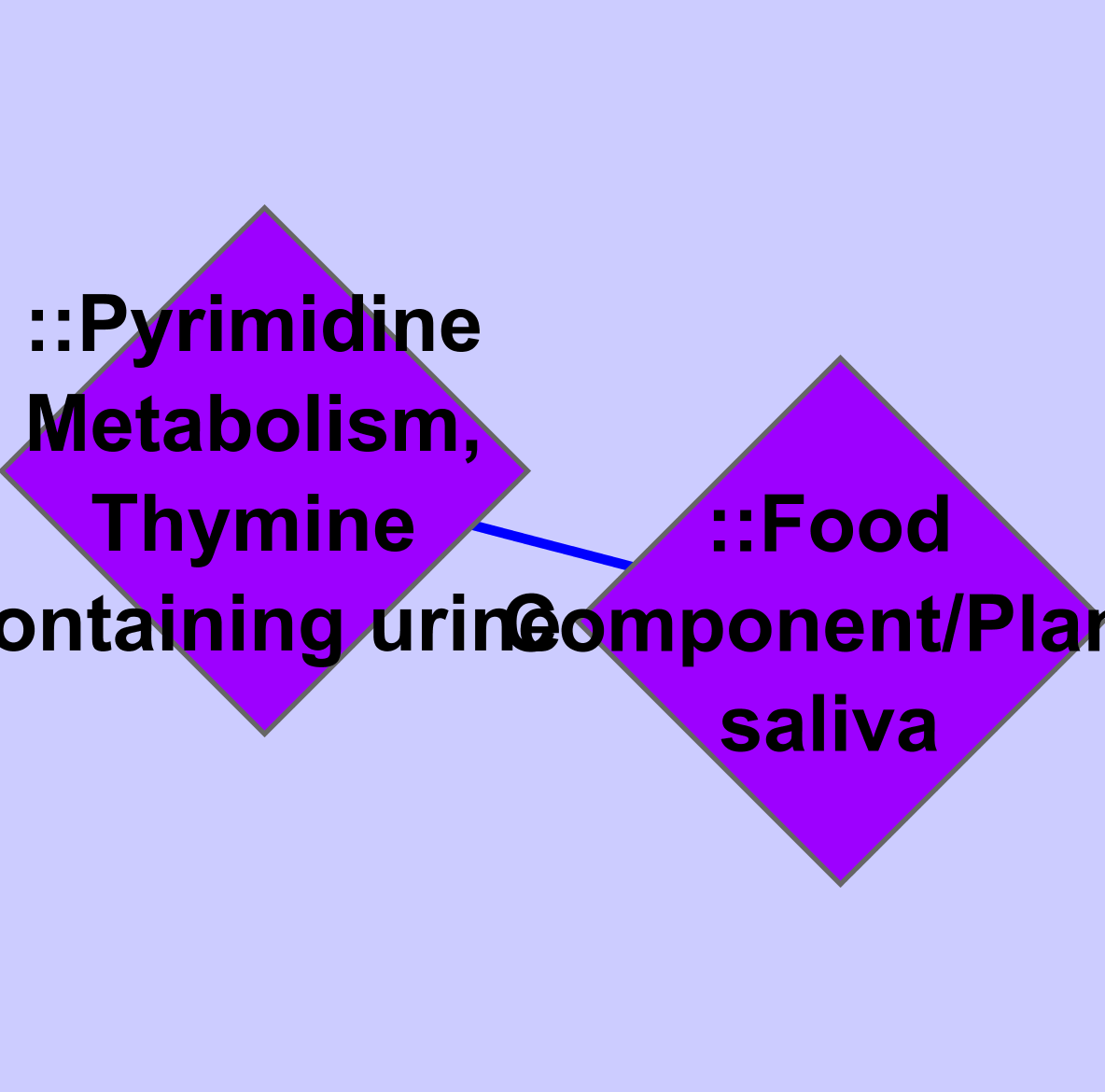

Supplement: Supplementary file 1 — Supplementary Material [file 41540_2017_29_MOESM1_ESM.zip › Supplement_onlineVersion/SupportingInformation_S10_Module identification code/ModuleIdentification/Results/levelSub/Simulated_modules_levelSub_module20.png]

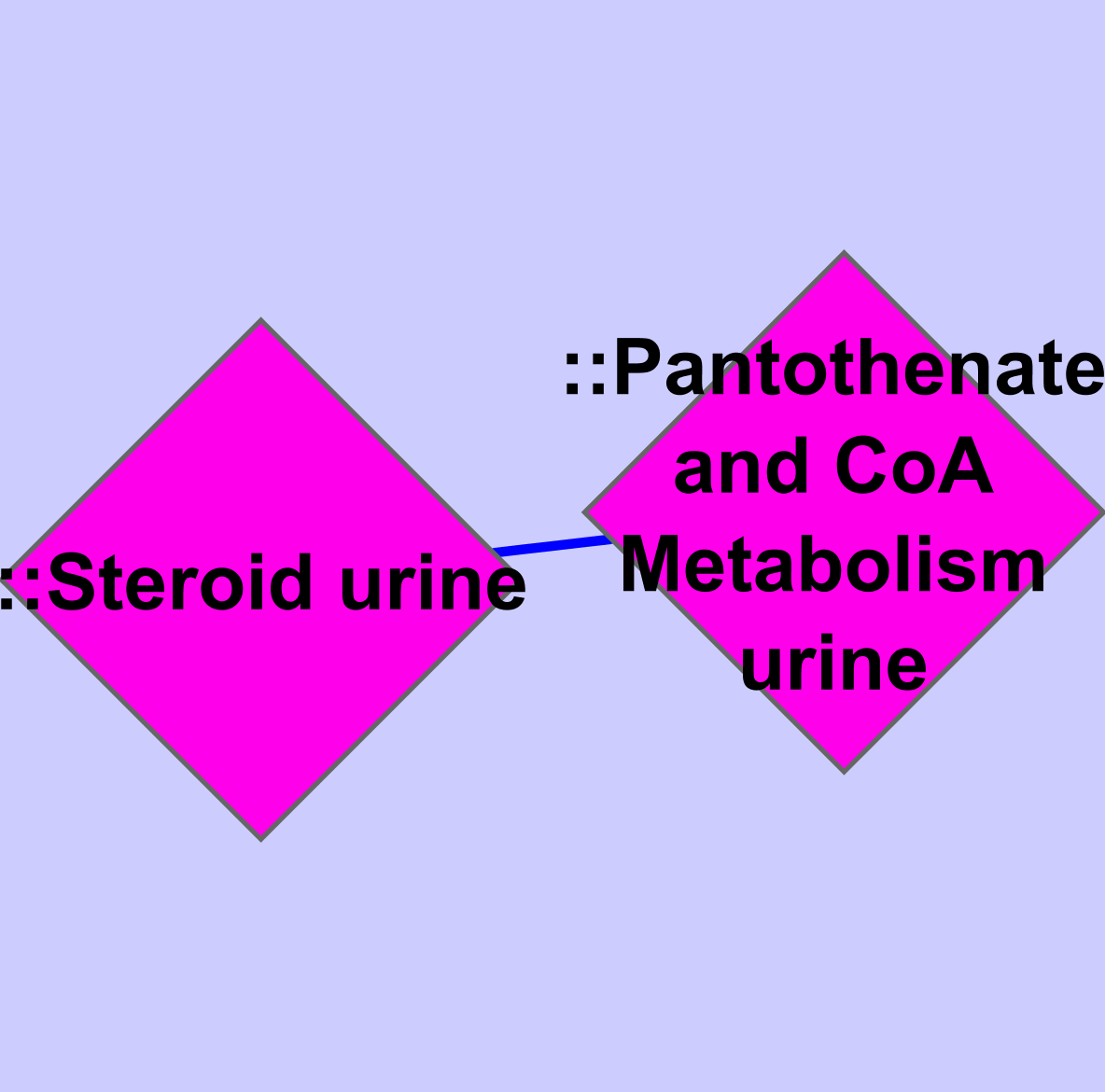

Supplement: Supplementary file 1 — Supplementary Material [file 41540_2017_29_MOESM1_ESM.zip › Supplement_onlineVersion/SupportingInformation_S10_Module identification code/ModuleIdentification/Results/levelSub/Simulated_modules_levelSub_module23.png]

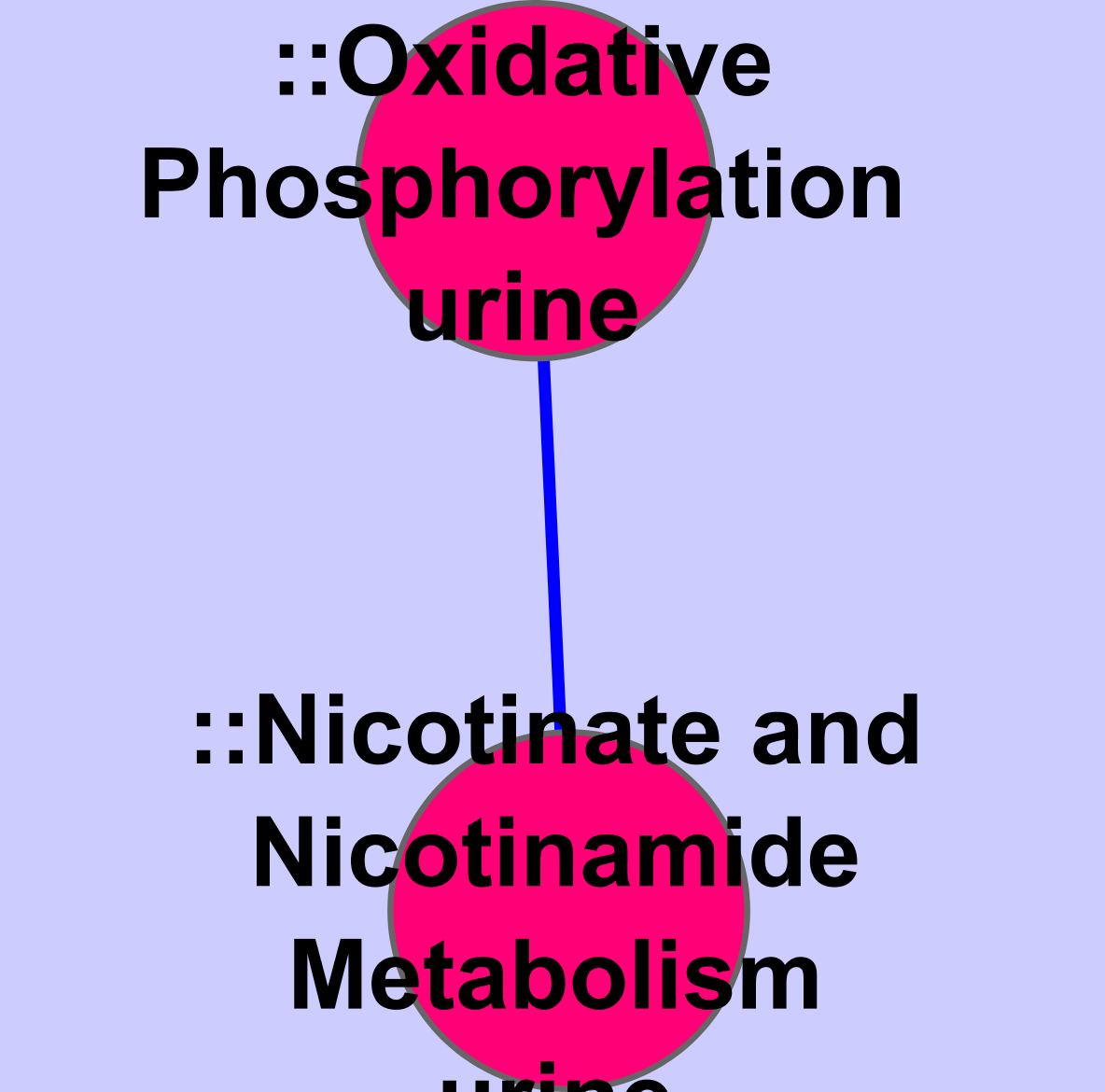

Supplement: Supplementary file 1 — Supplementary Material [file 41540_2017_29_MOESM1_ESM.zip › Supplement_onlineVersion/SupportingInformation_S10_Module identification code/ModuleIdentification/Results/levelSub/Simulated_modules_levelSub_module24.png]

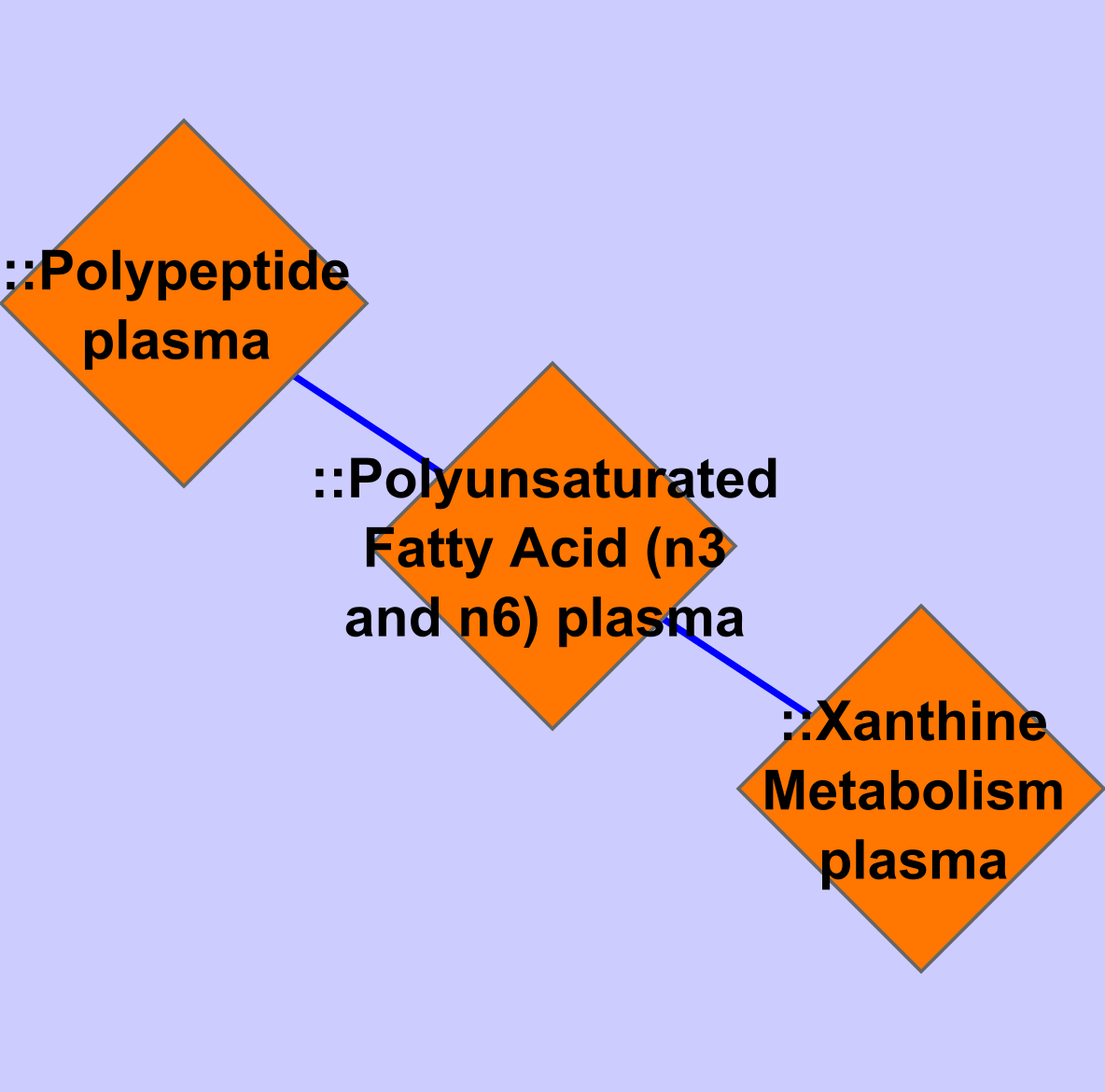

Supplement: Supplementary file 1 — Supplementary Material [file 41540_2017_29_MOESM1_ESM.zip › Supplement_onlineVersion/SupportingInformation_S10_Module identification code/ModuleIdentification/Results/levelSub/Simulated_modules_levelSub_module5.png]

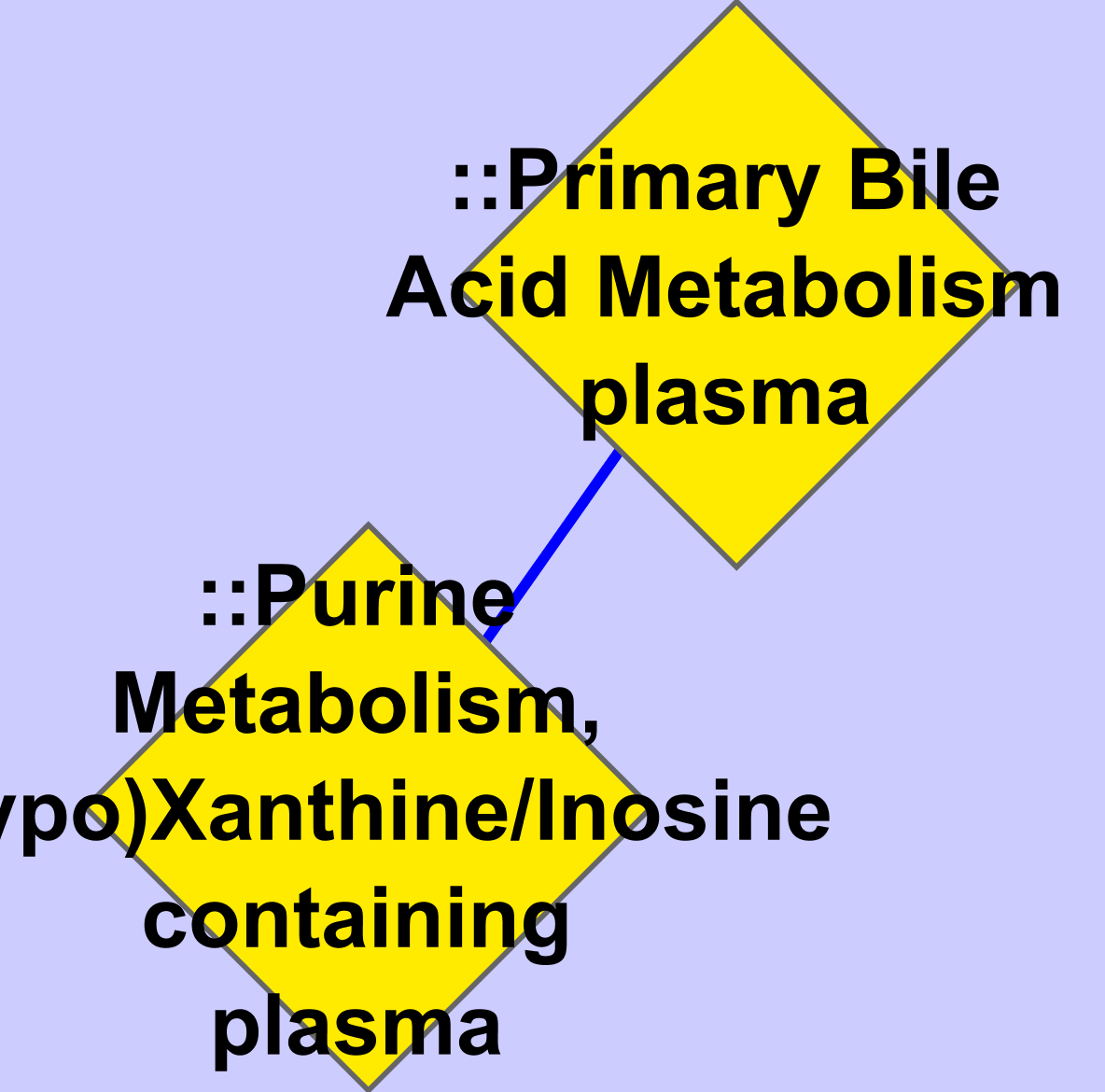

Supplement: Supplementary file 1 — Supplementary Material [file 41540_2017_29_MOESM1_ESM.zip › Supplement_onlineVersion/SupportingInformation_S10_Module identification code/ModuleIdentification/Results/levelSub/Simulated_modules_levelSub_module6.png]

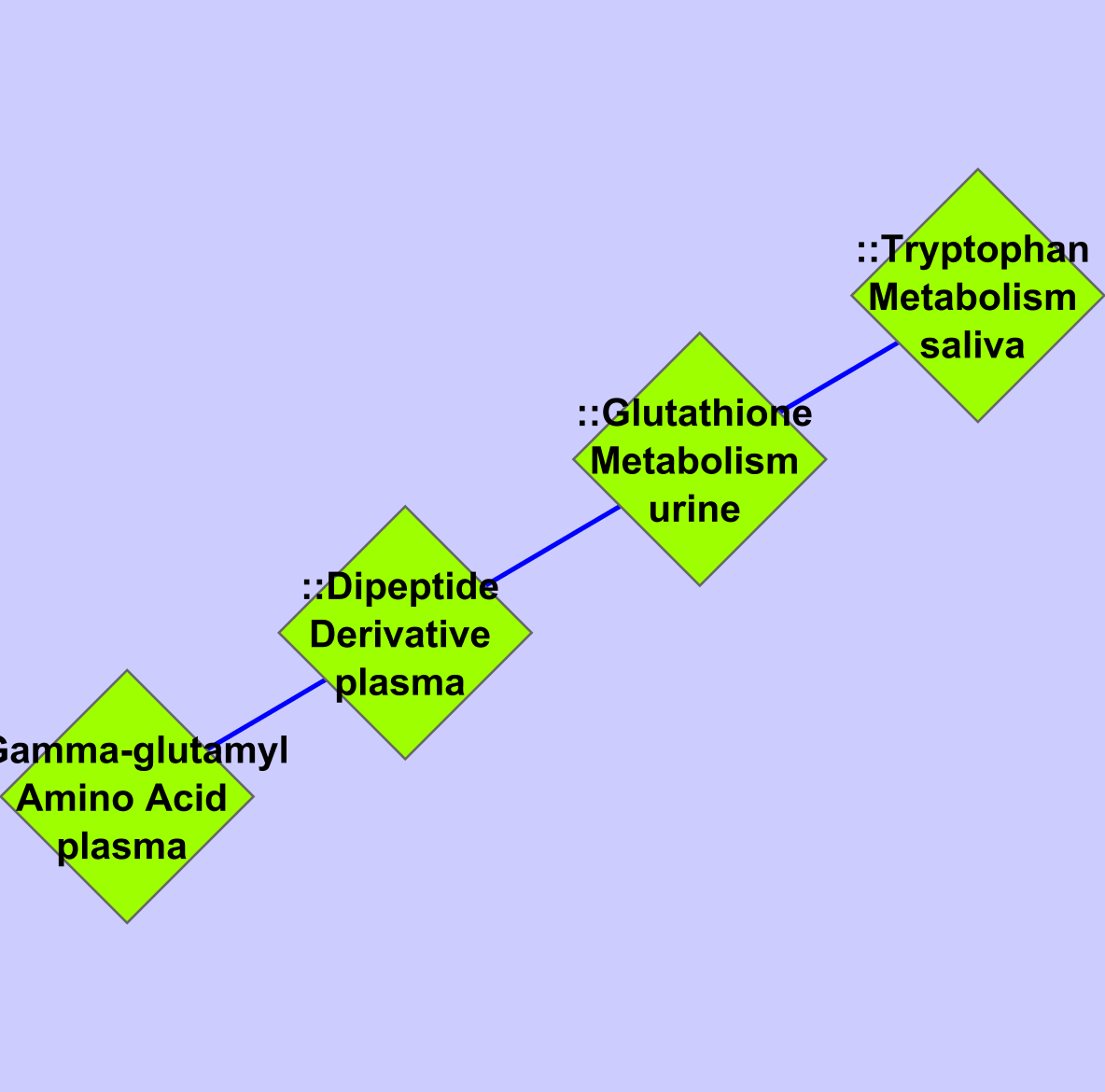

Supplement: Supplementary file 1 — Supplementary Material [file 41540_2017_29_MOESM1_ESM.zip › Supplement_onlineVersion/SupportingInformation_S10_Module identification code/ModuleIdentification/Results/levelSub/Simulated_modules_levelSub_module7.png]
